# Oligoethylene Phosphoramidate‐Based Kinase Inhibitor Prodrugs – Solubility, Enzyme Inhibition, and Hydrolysis

**DOI:** 10.1002/chem.202404618

**Published:** 2025-01-29

**Authors:** Sarah Spiewok, Maximilian Schaefer, Markus Lamla, Yannick Jaritz, Alexander J. C. Kuehne

**Affiliations:** ^1^ Institute of Organic and Macromolecular Chemistry Ulm University Albert-Einstein-Allee 11 89081 Ulm Germany

**Keywords:** PEG-drug conjugates, pH trigger, Water solubility, Drug functionalization

## Abstract

The efficiency of kinase inhibiting cancer therapeutics is often limited by their poor solubility in water. PEGylation is one possible strategy to improve the solubility of the drug, however, means to cleave off the PEG after reaching the target is important to make use of the therapeutic effects of the native drug. Moreover, the length of the PEG chains will have an effect on the solubility and binding. In this study, we want to extend our understanding of solubilizing oligo ethylene glycol (OEG) chains connected to kinase inhibitors using a pH labile phosphoramidate linker. We synthesize a library of drug‐OEG conjugates for Ceritinib, Crizotinib, Palbociclib, and Ribocilib kinase inhibitors with n=2, 3, 4 and 8 OEG repeat units. We study the influence on water solubility, enzyme inhibition, and pH induced hydrolysis. A maximum in solubility is obtained for n=3 or 4. We show that small differences in chain length can strongly influence the water solubility, while all other properties remain relatively comparable.

## Graphical abstract

**Kinase inhibitors** are functionalized with oligoethylene glycol chains via a pH‐labile linker, improving water solubility (optimal at n=3 – 4) while retaining enzyme inhibition and hydrolysis properties. This study highlights the impact of chain length on solubility, providing a versatile strategy for designing kinase inhibitor prodrugs.

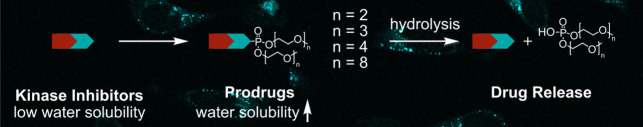

## 1 Introduction

Targeting kinases has become a pivotal strategy in modern cancer therapy, especially when tumor‐specific mutations can be exploited. Compared to traditional chemotherapy, such anti‐cancer kinase inhibitors offer improved efficacy, due to their specificity towards individual kinases in tumors.^[1]^ Examples include Ceritinib and Crizotinib, both of which are used in treating anaplastic lymphoma kinase (ALK)‐positive non‐small cell lung cancer. ALK dysregulation drives tumor growth and progression, so inhibiting ALK, alongside other growth‐promoting kinases, has yielded strong response rates.[^2^, ^3^, ^4^, ^5^, ^6^] Another class of growth‐targeting kinase inhibitors includes cyclin‐dependent kinase (CDK) inhibitors, such as Palbociclib and Ribociclib, which target CDK4/6, a key regulator of the cell cycle. These kinase inhibitors are primarily used for the treatment of breast cancer.[^7^, ^8^, ^9^, ^10^] However, despite their therapeutic benefits and convenient oral administration, kinase inhibitors often suffer from limited bioavailability, due to their poor solubility in aqueous media, restricting their effectiveness during oral as well as intravenous administration.[^1^, ^11^] The solubility in aqueous media can be improved by functionalization with water soluble polymers like polyethylene glycol (PEG). Besides improving the water solubility, PEGylation offers a promising strategy to also reduce immunogenicity, and prolong the circulation time in the body.[^12^, ^13^, ^14^, ^15^] Historically, PEGylation has focused on macromolecular drugs, such as proteins and enzymes, which becomes evident by the higher fraction of approved PEGylated protein‐based drugs compared to small‐molecular drugs.[^12^, ^14^] Improving the solubility of hydrophobic small‐molecule drugs could significantly enhance their pharmaceutical potential.^[16]^ The type of PEGylation (reversible or irreversible) and the PEG chain length will have substantial impact on the absorption rates and mechanisms, as observed in fluorescently labeled PEG conjugates with molar masses between 550 to 20.000 Da.[^17^, ^18^] On the contrary, polydispersity – inherent to every polymer – can complicate purification, formulation, and dosing of PEGylated drugs, leading to ill‐defined drug‐conjugate mixtures and undesired side reactions. To overcome these detrimental effects, oligoethylene (OEG) chains with a precisely defined number of repeat units can be employed to improve the solubility of hydrophobic drugs. As such, OEGylation allows for better reproducibility, as well as for reduced immunogenicity and antigenicity compared to PEGylated drugs.^[19]^ For the OEGylation of drugs, the commercial availability and cost of the discrete chain lengths are important. Defined OEG chains with less than eight repeat units (n ≤8) are generally less costly and available in high purity, making these chain lengths favorable for experimental exploration.[^19^, ^20^] For example, naloxegol and oxycodegol are two OEGylated opioid derivatives for the treatment of pain. OEGylation with n=6 (oxycodegol) and n=7 (naloxegol) improves bioavailability and enhances the water solubility of these drugs.[^12^, ^19^]

**Figure 1:**
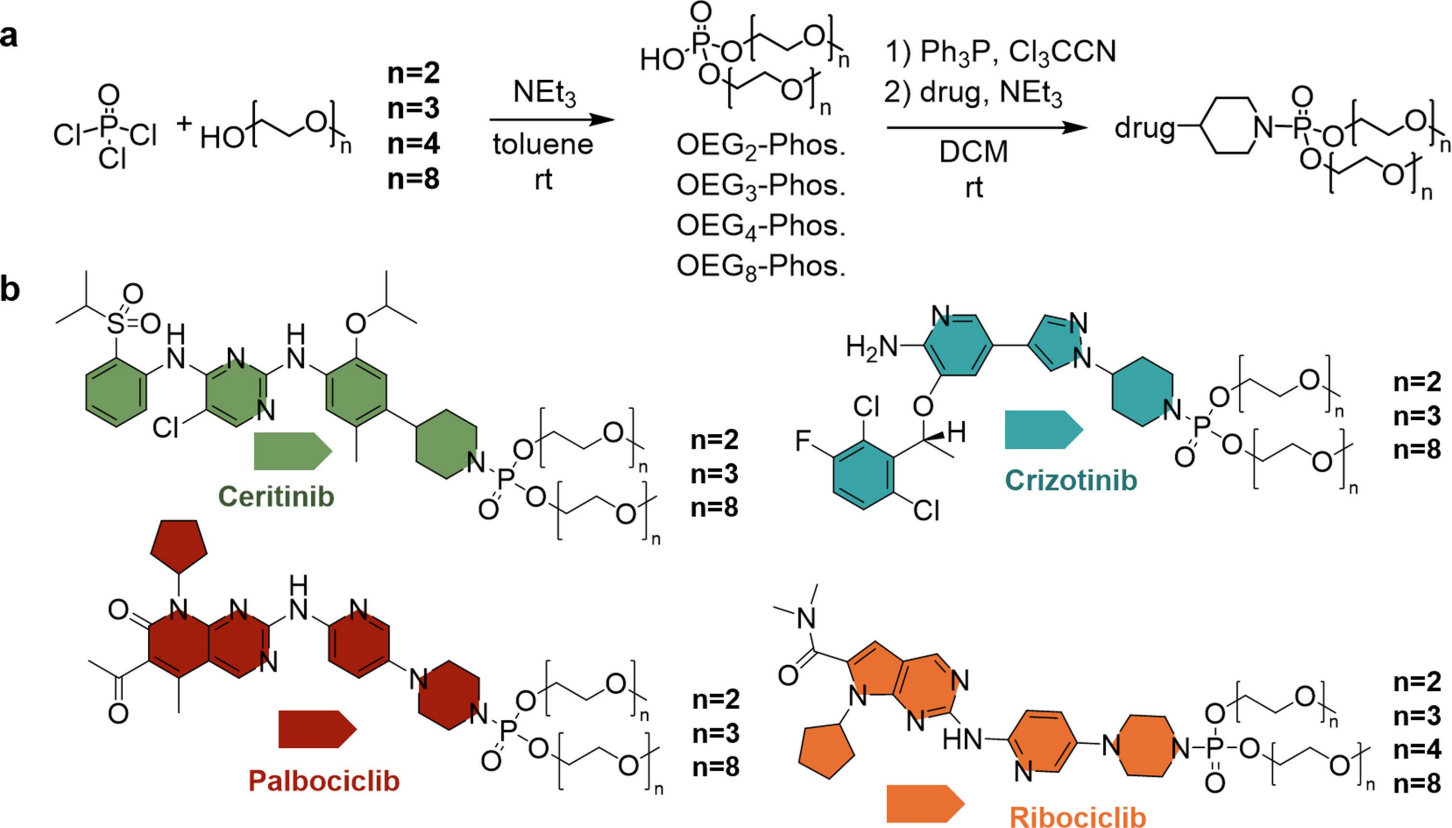
(**a**) Synthesis of OEG‐phosphates and functionalization of the kinase inhibitors. (**b**) Molecular structures of the synthesized prodrugs: Ceritinib (Cer) drug‐conjugates (green), Crizotinib (Cri) drug‐conjugates (blue), Palbociclib (Pal) drug‐conjugates (red) and Ribociclib (Rib) drug‐conjugates (orange). OEG chain lengths vary for each drug‐conjugate with n=2, 3 and 8 repeat units. For Ribociclib the OEG chain length varies from n=2, 3, 4, to 8 repeat units.

In our previous work, we have developed three kinase inhibitor prodrugs (from Ceritinib, Crizotinib, and Palbociclib).^[21]^ To obtain these prodrugs, we phosphoramidated the amine functionality of the native kinase inhibitors using phosphoric acid OEG diesters (see Figure 1a). The resulting prodrugs exhibit improved water solubility and cellular uptake, as well as reduced binding affinity to kinases. Interestingly, the phosphoramidate group can be cleaved in acidic medium, releasing the intact kinase inhibitor and reinstating the activity of the native drug. However, our previous investigation only considered a single OEG chain length and the influence of the OEG length variation on the prodrug performance remains unknown. Knowledge of the effect of OEG chain length on the prodrug solubility, uptake and release kinetics would allow to transfer the generalized prodrug concept to other kinase inhibitors. However, to date, there is a lack of prodrug concepts for the highly potent class of kinase inhibitor cancer drugs and the effect of OEGylation on their properties.

In this work, we investigate the effect of different OEG chain lengths for a set of four kinase inhibitors. We functionalize Ceritinib, Crizotinib, and Palbociclib with OEG chains of n=2, 3, and 8, and we show that our concept is transferrable to a fourth kinase inhibitor, Ribociclib (see Figure 1a). The study shows that OEG chain lengths of n=3 or 4 yield the best combined properties in all four kinase inhibitors.

## 2 Results and Discussion

### 2.1 Synthesis and Characterization of OEGylated Prodrugs

We first synthesize the bisOEGylated phosphate in accordance with our previously reported method.^[21]^ We vary the number of ethylene glycol repeat units from n=2 (OEG_2_‐Phos.), and n=3 (OEG_3_‐Phos.) to n=8 (OEG_8_‐Phos., see Figure 1a, and S16–21 for characterization). The reaction yields vary between 6 % and 42 %, for the different OEG functionalization approaches. As observed previously, the reaction between the OEG and the phosphoryl chloride leads to a statistical mix of mono‐, bis‐ and tris‐OEGylated phosphates. For purification, we perform an extraction of the aqueous solution at different pH.^[21]^ Especially shorter OEG chains with n=2 and n=3, are difficult to separate, decreasing the overall yield. Additional extraction steps can improve the yield but the values for n=2 and n=3 remain significantly lower than for n=4^[21]^ and n=8. The best yields are obtained for OEG_8_‐phosphate.

We synthesize a drug conjugate for each kinase inhibitor and each OEG chain length, resulting in a library of 13 drug conjugates (**Cer‐OEG_2_**, **Cer‐OEG_3_**, **Cer‐OEG_8_**, **Cri‐OEG_2_**, **Cri‐OEG_3_**, **Cri‐OEG_8_**, **Pal‐OEG_2_**, **Pal‐OEG_3_**, **Pal‐OEG_8_**, **Rib‐OEG_2_**, **Rib‐OEG_3_**, **Rib‐OEG_4_**, **Rib‐OEG_8_**) (see **Figure** 1 **b,** and **S22–47** for characterization).[^21^, ^22^] Since **Cer‐OEG_4_**, **Cri‐OEG_4_**, and **Pal‐OEG_4_** have been reported before, we rely on our previously published data for these drug conjugates.

### 2.2 Solubility Limit in Water and Self‐Assembly

**Figure 2:**
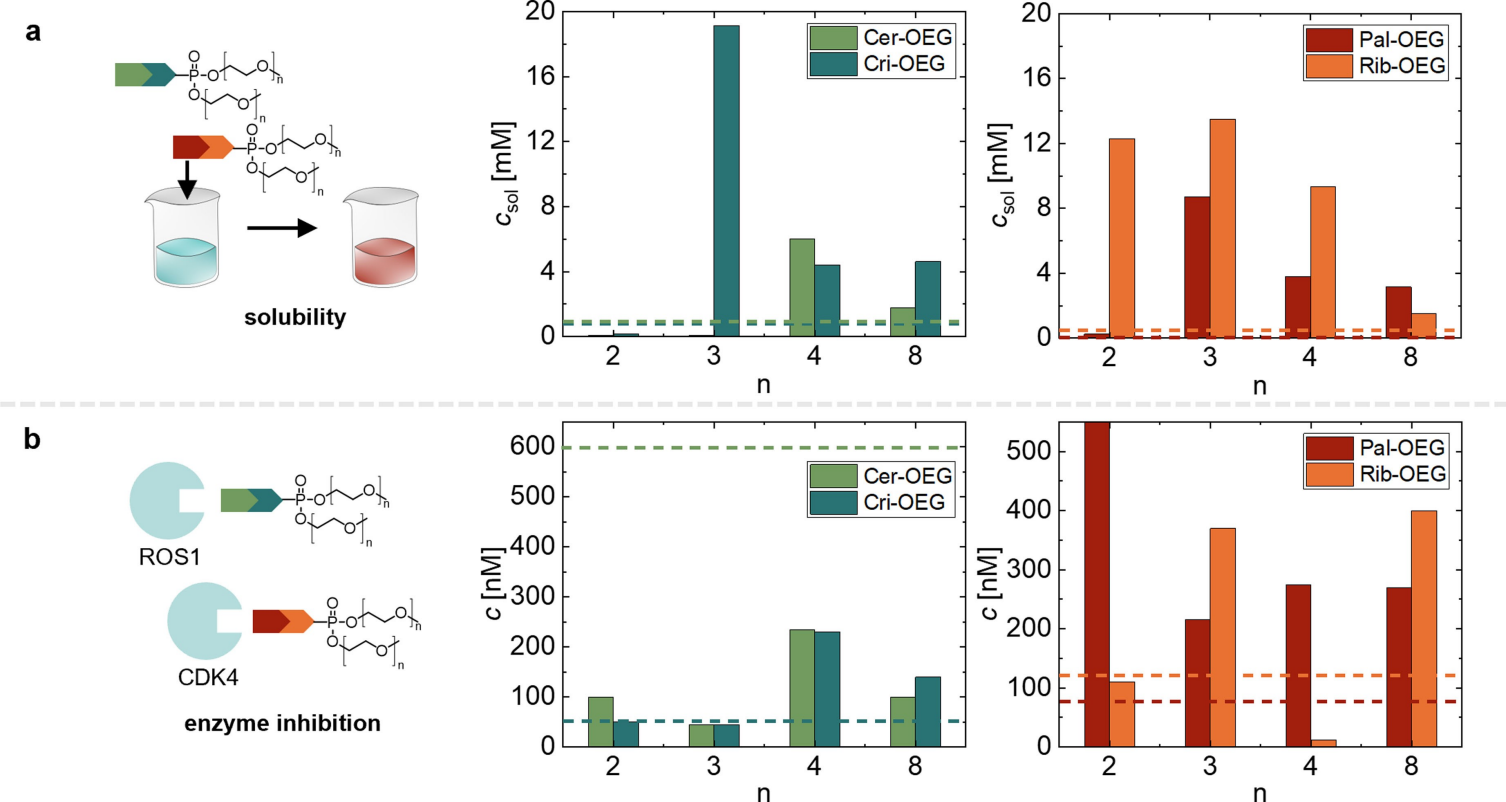
Influence of OEG chain length (n=2, 3, 4, and 8) of different drug‐conjugates (**Cer‐OEG_n_**, **Cri‐OEG_n_**, **Pal‐OEG_n_** and **Rib‐OEG_n_**). Values for n=4 of Cer‐OEG, Cri‐OEG, and Pal‐OEG are taken from reference [21]. (**a**) Water solubility (*c*[mM]) of drug‐conjugates determined from absorption at λ=600 nm. Dotted lines correspond to the properties of the native drugs. (**b**) Enzyme inhibition: IC_50_ values of **Cer‐OEG_n_** and **Cri‐OEG_n_** against ROS1 and **Pal‐OEG_n_** and **Rib‐OEG_n_** against CDK4. Dotted lines correspond to the properties of the native drugs.

With all purified compounds in hand, we start to analyze the different properties of the conjugates and discuss the influence of the OEG‐chain lengths. We determine the solubility in water with UV/Vis spectroscopy by measuring the optical density at 600 nm. The absorption (*A*) is plotted against varying concentration (*c*) of the drug conjugates (see Figure S1). In the range, in which the drug and their conjugates are still soluble, *A* can be fitted linearly. When the solubility limit is reached, the drug crashes out of solution leading to scattering and reduced transmission manifesting a threshold, upon which the increase in absorption is fitted with a second line of higher slope. The intersection between the two linear fits gives the critical concentration for the kinetic solubility *c*_sol_. Detailed solubility plots can be found in the supporting information (see Figure S1). An overview of all determined solubilities is provided in Figure 2 and Table 1. In our previous study with n=4, the solubility of all three kinase inhibitor prodrugs in water is improved over the native drug. However, the extent of improvement varies between the different kinase inhibitor prodrugs. For a better overview, we have included the performance of our previous study (see Figure 2).^[21]^ Previously, the solubility limit for Ceritinib has been determined to be *c*_sol_=6 mM with an OEG chain length of n=4. OEGylation with n=8 delivers poorer solubility of 1.75 mM; however, this is still better than for the native Ceritninib with a limit of 0.93 mM (see dashed line in Figure 2a).^[21]^ Shorter OEG chains with n=2 and n=3 do not deliver improved solubility over the native Ceritinib (*c*_sol_=0.07 and 0.08 mM, respectively, see light green data in Figure 2a). The maximal water solubility for Crizotinib is found for n=3 with *c*_sol_=19.14 mM, a factor of 25 better than the native drug. For n=4 and n=8 the solubility is still higher than for the native drug and only for OEG‐chains with n=2 the solubility is reduced to *c*_sol_=0.17 mM, below the native drug, see dark green data in Figure 2a. Very similar behavior is observed for Palbociclib‐conjugates. We find the maximal solubility of 8.71 mM for n=3. OEG chains with n=4 and n=8 repeat only slightly improve the solubility, while n=2 delivery 0.25 mM just above the solubility of the native drug (see red data in Figure 2a). For the additional CDK‐inhibitor Ribociclib, we determine a solubility of 0.49 mM in water. We observe an improvement of solubility upon conjugation with all of our different OEG‐phosphates. Maximal solubility is observed for n=3 with 13.49 mM, closely followed by 12.30 mM for n=2, and 9.33 mM for n=4. For 8 repeat units we determine 1.52 mM as the solubility limit.

**Table 1:** Summary of solubility limit in water *c*_sol_, inhibitory potential IC_50_, and hydrodynamic diameter *d* of drugs before and after OEGylation. Values marked with ^a^ are taken from our previous publication and added for better comparability.^[21]^

	*c*_sol_ (water) [mM]	IC_50_ (ROS1) [nM]	IC_50_ (CDK4) [nM]	*d* [nm]
Ceritinib	≤0.93^a^	600 ^a^	–	‐
Cer‐OEG_2_	≤0.07	100	–	565
Cer‐OEG_3_	≤0.08	45	–	279
Cer‐OEG_4_	≤6 ^a^	235 ^a^	–	3–10 ^a^
Cer‐OEG_8_	≤1.75	100	–	5
Crizotinib	≤0.77 ^a^	50 ^a^	–	‐
Cri‐OEG_2_	≤0.17	50	–	151
Cri‐OEG_3_	≤19.14	45	–	3.7
Cri‐OEG_4_	≤4.4 ^a^	230 ^a^	–	3–10 ^a^
Cri‐OEG_8_	≤4.62	140	–	3.3
Palbociclib	≤0.02 ^a^	–	85 ^a^	–
Pal‐OEG_2_	≤0.25	–	1000	264
Pal‐OEG_3_	≤8.71	–	215	5
Pal‐OEG_4_	≤3.8 ^a^	–	275 ^a^	3–10 ^a^
Pal‐OEG_8_	≤3.16	–	270	596
Ribociclib	≤0.49	–	120	–
Rib‐OEG_2_	≤12.30	–	110	2.5
Rib‐OEG_3_	≤13.49	–	370	1.8
Rib‐OEG_4_	≤9.33	–	12	92
Rib‐OEG_8_	≤1.52	–	400	64

Overall, we find the best improvements of solubility for n=3 (except for Ceritinib). Surprisingly, the solubility for longer chain lengths is reduced, which may be connected to the formation of micelles in water. We further assume that OEG‐chains of n=8 may be able to crystallize leading to a reduction in solubility.

To study, whether self‐assembly into micelles might have an effect on the solubility limit of the prodrugs, we perform dynamic light scattering (DLS) of the drug‐conjugates in water. We prepare solutions at the critical solution concentrations (*c*_sol_) and determine the hydrodynamic diameter (*d*) or the formed micelles or aggregates. Overall, substances with significant water solubility (>1 mM) deliver *d* below 100 nm (except for **Pal‐OEG_8_**), hinting at the formation of spherical micelles (see Table 1 and **Figure** **S2–S4**). Micelles below 6 nm (**Cer‐OEG_8_**, **Cri‐OEG_3_**, **Cri‐OEG_8_**, **Pal‐OEG_3_**, **Rib‐OEG_2_**, and **Rib‐OEG_3_**) are potentially affected by renal clearance. Drug‐conjugates with a solubility below 1 mM (**Cer‐OEG_2_**, **Cer‐OEG_3_**, **Cri‐OEG_2_** and **Pal‐OEG_2_**) tend to form larger objects with *d* above 250 nm. These data suggest, that drug conjugates with n=3 or 4 might exhibit the right lipid shape to form spherical micelles, leading to the high solubility. We assume that the self‐assembly influences primarily the water solubility, as we do not observe any correlations regarding enzyme inhibition or hydrolysis.

### 2.3 Inhibition Potential

Apart from solubility and self‐assembly, we investigate the inhibitory potential against the respectively targeted receptors. For Ceritinib and Crizotinib conjugates we chose ROS proto‐oncogene 1 (ROS1) as a target in a cell free enzyme assay. For Palbociclib and Ribociclib conjugates we chose cyclin‐dependent kinase 4 (CDK4) as a binding target in a cell free enzyme assay. Previously, the native Ceritinib drug molecule showed an IC_50_ of 600 nM against ROS1 (see dotted light green line in Figure 2b).^[21]^ Here, the tested prodrug conjugates show even lower IC_50_ of 45 nM against ROS1 (thus better binding) for the well soluble **Cer‐OEG_3_** and 100 nM for **Cer‐OEG_2_** and **Cer‐OEG_8_** (see Figure 2b and S6). This improved binding was also observed previously for **Cer‐OEG_4_** (see Figure 2b).^[21]^ The solubility‐mediating OEG‐chains appear to be advantageous for the inhibition of ROS1. Interestingly, this behaviour cannot be generalized to the other kinase inhibiting prodrug constructs. When looking at the binding affinities of the OEG‐functionalized Crizotinib prodrugs, we find very similar or higher IC_50_ values as for the native Crizotinib (IC_50_: 50 nM) (see dark green line in Figure 2b and Figure S7).^[21]^ In both ROS1 targeting drugs, the functionalized piperidine ring is located away from the receptor domain of the molecule and is therefore not directly involved in the binding.[^8^, ^23^, ^24^] Nevertheless, we expect longer OEG‐chains to interfere more strongly with the receptor binding leading to higher IC_50_ values – a hypothesis, which appears to be valid for Crizotinib.

When investigating the CDK inhibitors, all OEGylated Palbociclib derivatives show a decrease in the inhibitory potential, which is the normal and desired working principle of a prodrug. For n=2 the IC_50_ is significantly increased to 1000 nM, whereas **Pal‐OEG_3_**, **Pal‐OEG_3_**, and **Pal‐OEG_8_**, all inhibit CDK4 in a similar concentration range between 215 nM and 270 nM respectively (see Figure 2b and S8). X‐ray crystal structure of Palbociclib binding to CDK6 (a structural homolog to CDK4) shows the piperazine unit sticking out of the binding pocket.[^23^, ^25^] In our prodrug molecules it is this piperazine unit that is functionalized with OEG, meaning it is not substantially involved in binding and inhibition. For Ribociclib we have to first establish the IC_50_ value for comparison to the OEG‐functionalized prodrug constructs. The IC_50_ of Ribociclib towards CDK4 is 120 nM. **Rib‐OEG_2_** and **Rib‐OEG_4_** show slightly decreased IC_50_ values of 110 nM and 12 nM, representing similar or even improved receptor inhibition (see Table 1 and Figure 2b). **Rib‐OEG_3_** and **Rib‐OEG_8_** show a slight increase in IC_50_ with 370 nM and 400 nM (see Table 1, Figure 2b and Figure S9). By design, the OEG‐functionalized part of the molecule does not take part in binding, therefore producing better target inhibition than the native drug for prodrugs that exhibit improved solubility. However, depending on the respective drug molecule, the amine functionality is suspected to take part in some hydrogen bonding with distant amino acids.[^26^, ^27^] Therefore, there is no direct correlation between improved solubility and improved binding. Moreover, n=8 seems to induce increases steric demand preventing easy access to the binding site and these all OEG_8_‐prodrugs have higher IC_50_ values than the native drug. (see Table 1).

### 2.4 Hydrolysis and Drug Release

**Figure 3:**
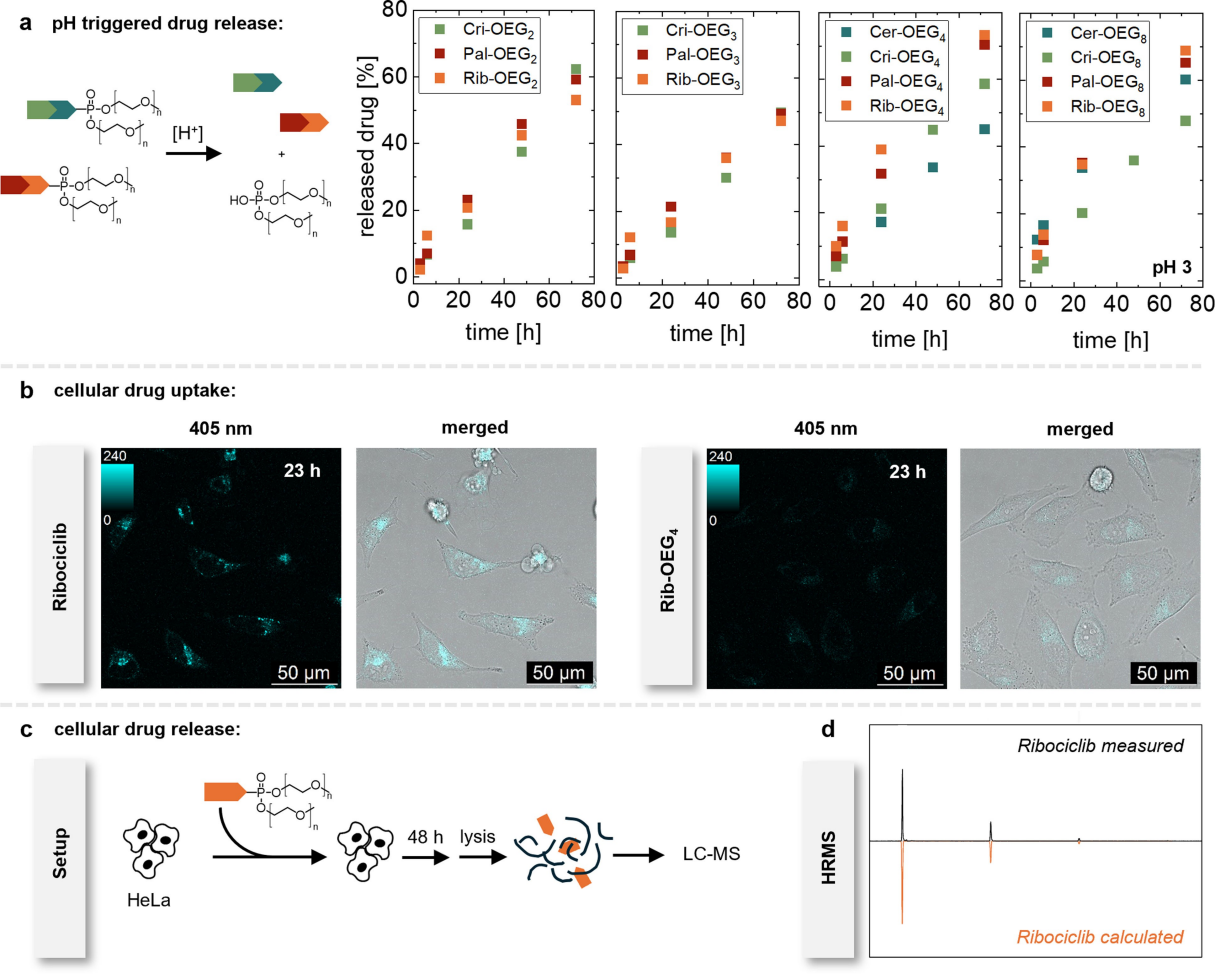
Influence of OEG chain length (n=2, 3, 4, and 8) of the release kinetics of the different drug‐conjugates (**Cer‐OEGn**, **Cri‐OEGn**, **Pal‐OEGn** and **Rib‐OEGn**). Values for n=4 of Cer‐OEG, Cri‐OEG, and Pal‐OEG are taken from reference [21]. (**a**) Drug release determined from HPLC analysis. Time (in h) is plotted against the released drug (%) at pH 3. Labeling of the y axis applies to all plots. (**b**) CLSM images of Ribociclib (25 μM) and **Rib‐OEG_4_** (25 μM) uptake in HeLa cells after 23 h. 405 nm: fluorescence channel after excitation at 405 nm. merged: fluorescence channel and bright field channel. (**c**) Schematic representation of the experimental procedure for cellular drug release studies for **Rib‐OEG_4_**. (**d**) ESI+ spectra of the detected Ribociclib after cell induced cleavage of the promoiety measured and calculated after chromatography of cell lysate.

The working principle of a prodrug is to improve solubility and circulation time and not necessarily to improve binding to the target. However, binding should of course become important when the prodrug has arrived at the designated site and the promoiety is cleaved to release the native drug. In case of the phosphoramidate linked OEG chains, release of the native drug is induced by hydrolysis at low pH.[^21^, ^28^, ^29^, ^30^, ^31^, ^32^] The stability of the phosphoramidate linker and therefore its half‐life depends on the chemical surrounding in the molecule.[^28^, ^29^] Since we only change distant substituents by varying the binding motif of the respective kinase inhibitor, we expect only minor influence on the rate of hydrolysis. We investigate the samples at pH 3, 4, 5, 6, and 7.4 over a course of 72 h at 37 °C using high pressure liquid chromatography (HPLC) (see Figure S10–13 and Table S1–11). We observe the fastest hydrolysis at pH=3, and we compare the half‐lives, that is the time (h) needed for 50 % drug release (see Figure 3a). We perform mass spectrometry of the released compounds to verify that the released molecule is the intact native drug and no degradation of the drug molecule takes place (see Figure S48–58).

The solubility of **Cer‐OEG_2_** and **‐OEG_3_** is too low to perform the hydrolysis experiment, which is why they are excluded from this study. We observe slow release for all tested conjugates even at pH 3, with **Cer‐OEG_4_** lasting 96 h^[21]^ and **Cer‐OEG_8_** requiring 50 h until 50 % of the prodrug have been released. All Crizotinib derivatives exhibit comparable release rates with half‐lives around 60 to 70 h. Also the Palbociclib‐ and Ribociclib prodrugs show a similar pattern, with, n=4 and 8 exhibiting half‐lives between 40 and 50 h, while constructs with n=2 and 3 show slightly higher stability and require approximately 55 to 75 h for release.

In general, all species are stable at physiological pH over the course of 72 h. Most conjugates show the fastest drug release for n=4 (except for Ceritinib prodrugs).

### 2.5 Cell Uptake and Release

Altogether an OEG chain length of n=4 seems to be favorable for producing prodrugs with improved solubility from kinase inhibitors. Previously, cell induced hydrolysis has been shown for OEG‐functionalized kinase inhibitors prodrugs of Ceritinib, Crizotinib, and Palbociclib. Here we therefore concentrate on **Rib‐OEG_4_**. We incubate HeLa cells with Ribociclib and **Rib‐OEG_4_** for 23 h while imagining them with confocal laser scanning microscopy (CLSM) (see Figure 3b and Figure S14–S15). The drug and its conjugate are exited at 405 nm, leading to weak fluorescence of the drugs. Both native and functionalized drugs seen to be taken up by the cells. In some spots the native Ribociclib appears to have formed aggregates, which will be an effect of the lower solubility.

We further perform a drug release study in the HeLa cells to show that the drug can be released also in biological environment. HeLa cells are treated with **Rib‐OEG_4_** for 48 h followed by cell lysis. The cell lysate is analyzed with liquid chromatography‐mass spectrometry (LC–MS). We find mainly the native Ribociclib, alongside some small **Rib‐OEG_4_** peaks, indicating that after 48 h most of the native drug has been released from the prodrug constructs (see Figure 3c and d). We can show that the released structure detected with HRMS is identical with the calculated mass of Ribociclib (see Figure 3d). This confirms that release of the drug is possible in cells and that the low pH in some of the cell compartments, such as the lysosomes, yield release of the effective drug molecule.

## 3 Conclusions

In summary, we show that the previously developed approach for the functionalization of secondary amines in kinase inhibitors is universally applicable to various OEG‐chains. Even quite short chains of n=3 produce a strongly increased solubility in water. In combination with binding performance and release kinetics the sweet spot seems to be at n=4. The rather slow drug release could render our approach useful to prepare kinase inhibitor prodrugs with long circulation times, especially when these assemble into micelles. We have extended our general approach for tuning the hydrophilicity of drugs, which can be extended to other prodrug approaches.

## Experimental Methods and Materials

*Materials*: All used chemicals are purchased from commercial suppliers and used without further purification. POCl_3_, diethylene glycol monomethyl ether and triethylene glycol monomethyl ether are purchased from Sigma Aldrich. Octaethylene glycol monomethyl ether is purchased from abcr. Ceritinib, Crizotinib, Palbociclib, and Ribocilib are purchased from BLDpharm. CDK4 and ROS1 kinase assay kits are purchased from biomol and Kinase Glo Max is purchased from Promega.

*General Synthetic Procedure for OEG‐Phosphate*: OEG‐Phosphates are synthesized according to previous strategy.^[21]^ OEG (2 eq.) is dissolved in dry toluene together with triethylamine (2 eq.). In a separate flask, phosphoryl trichloride (1 eq.) is dissolved in toluene under water and oxygen free conditions. The solution is cooled to 0 °C and the solvated OEG solution is added dropwise. The mixture is allowed to warm to room temperature and is stirred overnight. The reaction is filtered and quenched with saturated Na_2_CO_3_ solution at 0 °C and stirred for 2 h. After hydrolysis, the solvent mix is removed by evaporation. For separation of different OEGylated‐phosphates, the crude product is dissolved in water and brought to a pH of 14 with NaOH. Washing with DCM (5x) removes the side‐product with the organic phase. The remaining aqueous solution is acidified with HCl (35 %) to a pH of 2. The desired two times substituted OEG‐phosphate is extracted with DCM (5x) and dried over magnesium sulphate. Solvent is removed to obtain the corresponding OEG‐phosphate.

*General Synthetic Procedure for Drug Conjugates*: Drug conjugate synthesis follows previous procedure.[^21^, ^22^] The OEG‐phosphate (1.1 eq.) is dissolved in DCM (2 mL). Triphenyl phosphine (2.2 eq.) and trichloro acetonitrile (2.3 eq.) are added to the solution. The reaction mixture is stirred for 1–2 h at rt. The drug (1.2 eq.) is dissolved together with triethyl amine (3.1 eq.) in DCM (1 mL) and added to the reaction mixture. The reaction is stirred for another 2–3 h at rt while monitored with TLC. The mixture is then diluted with DCM and washed with 1 M HCl, followed by sat. aq. NaHCO_3_. The organic layer is dried with magnesium sulphate and concentrated in vacuo. The crude material is purified using column chromatography (EtOAc: 20 % MeOH, if not specified otherwise for the specific substance) to give the desired drug conjugate.

*NMR*: ^1^H‐NMR and ^31^P‐NMR measurements are performed on 400 MHz BrukerAvance 400 spectrometer. The spectra are referenced to the solvent peak (^1^H: DMSO‐d_6_ 2.5 ppm, CDCl_3_ 7.26 ppm). The data is assessed and evaluated with MestReNova (Mestrelab Research).

*Dynamic Light Scattering (DLS)*: Possible self‐assemblies are investigated with DLS Nano‐Zetasizer (Malvern Instruments) at 25 °C with a scattering angle of 173° at λ=633 nm.

*UV‐Vis Spectroscopy*: Changes in solubility are measured with a Lambda 365 spectrometer by PerkinElmer supplied with UV WinLab software. Samples are prepared in water and placed in a 1 cm quartz cuvette.

*High Performance Liquid Chromatography (HPLC)*: Drug release is monitored on Agilent 1260 Infinity containing the following modules: G1311B Quat Pump, G1329B ALS, G1365D MWD VL in combination with a Jetstream 2 plus column thermostat. Reverse phase column (Poroshell 120, EC−C18, 3×100 mm, 2.7 μm) is temperature to 40 °C. Flow rate is set to 0.3 mL/min and absorption is detected at 254 nm. Solvent mixture contains water (65–0 %), acetonitrile (15–80 %) and 0.1 % aqueous formic acid solution (20 %) for **Cer‐OEG_n_**, **Cri‐OEG_n_** and **Pal‐OEG_n_**. For **Rib‐OEG_n_**, solvent mixture contains water (80–30 %), acetonitrile (0–50 %) and 0.1 % aqueous formic acid solution (20 %).

*Enzyme Binding Studies (ROS1)*:^[21]^ ROS1 assays are performed with the following substances: **Cer‐OEG_2_**, **Cer‐OEG_3_**, **Cer‐OEG_8_**, **Cri‐OEG_2_**, **Cri‐OEG_3_** and **Cri‐OEG_8_**. The instructions are followed with some specifications: DTT (10 μL of 1 M DTT to 1 mL buffer) is added to the buffer solution and the assay is performed with 1 % DMSO in the final reaction volume. Serial dilutions of inhibitor (5 μL) are mixed with 20 μL enzyme (5 ng/μL), 5 μL of IGF‐1Rtide (1 mg/mL) and 1 μL ATP solution (500 μM) in a 96‐well plate. The blank contains no enzyme and no inhibitor. The positive control contains enzyme but no inhibitor. The receptor inhibitory rate is calculated from the luminescence as IC_50_. The IC_50_ is defined as the drug concentration required to inhibit the receptor by 50 % relative to the controls. Luminescence is recorded on Promega GloMax Navigator Microplate Luminometer. Samples are prepared in duplicates.

*Enzyme Binding Assays (CDK4)*:^[21]^ CDK4 assays are performed with the following substances: **Pal‐OEG_2_**, **Pal‐OEG_3_**, **Pal‐OEG_8_**, **Ribociclib**, **Rib‐OEG_2_**, **Rib‐OEG_3_**, **Rib‐OEG_4_** and **Rib‐OEG_8_**. The instructions are followed with some specifications: DTT (10 μL of 1 M DTT to 1 mL buffer) is added to the buffer solution and the assay is performed with 1 % DMSO in the final reaction volume. Serial dilutions of inhibitor (5 μL) are mixed with 20 μL enzyme (10 ng/μL), 5 μL of 10x CDK4 substrate peptide and 1 μL ATP solution (500 μM) in a 96‐well plate. The blank contains no enzyme and no inhibitor. The positive control contains enzyme but no inhibitor. The receptor inhibitory rate is calculated from the luminescence as IC_50_. The IC_50_ is defined as the drug concentration required to inhibit the receptor by 50 % relative to the controls. Luminescence is recorded on Promega GloMax Navigator Microplate Luminometer. Samples are prepared in duplicates.

*pH Dependent Hydrolysis*:^[21]^ OEGylated drugs are dissolved in buffer with a final concentration of 1–2 mM. For pH 3, 4 and 5 a 0.1 mM acetate buffer is used. For pH 6 and 7.4 we use a 0.1 mM HEPES buffer. Samples are tempered to 37 °C and aliquots are removed at different time points. Aliquots are diluted with 400 μL HPLC solvent (**Cer‐OEG_n_**, **Cri‐OEG_n_** and **Pal‐OEG_n_**: 65 % water, 15 % acetonitrile and 20 % 0.1 % aqu. formic acid; **Rib‐OEG_n_**: 80 % water and 20 % 0.1 % aqu. formic acid). Drug release percentages are calculated form the ratio of the peak area of drug and drug‐OEG conjugate for 0, 3, 6, 24, 48 and 72 h.

*Cell Uptake*: Cell uptake studies are performed according to previous publication with 25 μM **Ribociclib** and **Rib‐OEG_4_**.^[21]^ Uptake is followed by Confocal for 23 h using Leica TCS SP8 Lightning confocal microscope with an OnkoLab incubator chamber with a constant temperature of 37 °C and a 5 % CO_2_ atmosphere at 95 % humidity (gas flow: 0.1 L/min). Drug and drug‐OEG conjugate is excited at 405 nm.

*Cellular Drug‐Release with LC–MS*: Drug release studies are performed according to previous publication with 20 μM of **Rib‐OEG_4_**.^[21]^

## Conflict of Interests

The authors declare no conflict of interest.

4

## Supporting information

As a service to our authors and readers, this journal provides supporting information supplied by the authors. Such materials are peer reviewed and may be re‐organized for online delivery, but are not copy‐edited or typeset. Technical support issues arising from supporting information (other than missing files) should be addressed to the authors.

Supporting Information

## Data Availability

The data that support the findings of this study are available in the supplementary material of this article.
