# Supplementary material for: Oligoethylene Phosphoramidate‐Based Kinase Inhibitor Prodrugs – Solubility, Enzyme Inhibition, and Hydrolysis
Source: Chemistry. 2025 Jan 29;31(12):e202404618. doi: 10.1002/chem.202404618 (PMC11855230; doi:10.1002/chem.202404618)
Supplement: Supplementary file 1 — Supporting Information [file CHEM-31-e202404618-s001.pdf]

Chemistry–A European Journal

Supporting Information

Oligoethylene Phosphoramidate-Based Kinase Inhibitor Prodrugs – Solubility, Enzyme Inhibition, and Hydrolysis

Sarah Spiewok, Maximilian Schaefer, Markus Lamla, Yannick Jaritz, and Alexander J. C. Kuehne*

Supporting Information

Oligoethylene Phosphoramidate-based Kinase Inhibitor Prodrugs – Solubility, Enzyme Inhibition, and Hydrolysis

*Sarah Spiewok, Maximilian Schaefer, Markus Lamla, Yannick Jaritz, and Alexander J. C. Kuehne**

Table of Contents

Synthesis and Characterization....3
Synthesis of OEG_2-Phosphate....3
Synthesis of OEG_3-Phosphate....3
Synthesis of OEG_8-Phosphate....3
Synthesis of Cer-OEG_2....3
Synthesis of Cri-OEG_2....4
Synthesis of Pal-OEG_2....4
Synthesis of Rib-OEG_2....4
Synthesis of Cer-OEG_3....5
Synthesis of Cri-OEG_3....5
Synthesis of Pal-OEG_3....5
Synthesis of Rib-OEG_3....6
Synthesis of Rib-OEG_4....6
Synthesis of Cer-OEG_8....6
Synthesis of Cri-OEG_8....7
Synthesis of Pal-OEG_8....7
Synthesis of Rib-OEG_8....7
Water Solubility....8
Dynamic Light Scattering (DLS)....9
Cell-free Binding Assays....13
pH Dependent Hydrolysis....17
Cell Uptake with Confocal Laser Scanning Microscopy (CLSM)....30
NMR Spectra....32
Mass Spectra....48

Synthesis and Characterization

Synthesis of OEG_2-Phosphate

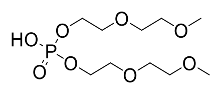

The reaction follows the general synthetic procedure for OEG-Phosphate using 8.7 g of diethylenglycolmethylether. OEG_2-phosphate is formed as oily solid (yield: 6%). ^{1}H-NMR (400 MHz, $CDCl_3$): δ= 4.28 – 4.16 (m, 3H), 3.77 – 3.64 (m, 8H), 3.60 – 3.51 (m, 4H), 3.40 (s, 6H) ppm. ^{31}P-NMR (162 MHz, $CDCl_3$): δ= 0.25 ppm. HRMS (ESI^-) ($C_{10}H_{22}O_8P^-$ calc.: m/z 301.1057): m/z= 301.1080.

Synthesis of OEG_3-Phosphate

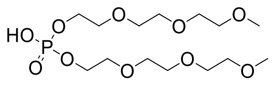

The reaction follows the general synthetic procedure for OEG-Phosphate using 11.8 g of triethylene glycol monomethyl ether. OEG_3-phosphate is formed as oily solid (yield: 11%). ^{1}H-NMR (400 MHz, $CDCl_3$): δ= 4.26 – 4.13 (m, 4H), 3.68 (dddd, J = 13.8, 6.8, 3.0, 1.7 Hz, 16H), 3.59 – 3.53 (m, 4H), 3.38 (s, 6H) ppm. ^{31}P-NMR (162 MHz, $CDCl_3$): δ= 0.00 ppm. HRMS (ESI^-) ($C_{14}H_{30}O_{10}P^-$ calc.: m/z 389.1582): m/z= 389.1619.

Synthesis of OEG_8-Phosphate

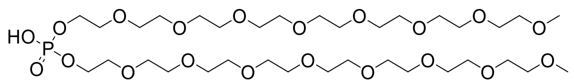

The reaction follows the general synthetic procedure for OEG-Phosphate using 5 g of Octaethylene glycol monomethyl ether. OEG_8-phosphate is formed as white slightly oily solid (yield: 42%). ^{1}H-NMR (400 MHz, DMSO-d_6): δ= 3.99 – 3.93 (m, 4H), 3.61 – 3.55 (m, 4H), 3.53 – 3.49 (m, 52H), 3.44 – 3.41 (m, 4H), 3.24 (s, 6H) ppm. ^{31}P-NMR (243 MHz, DMSO-d_6): δ= -1.36. ppm. HRMS (ESI^+) ($C_{34}H_{70}Na_2O_{20}P^+$ calc.: m/z 875.3988): [$M+2Na^+$] m/z= 875.39468.

Synthesis of Cer-OEG_2

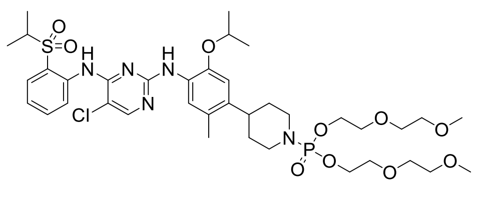

The reaction follows the general synthetic procedure for Drug-OEG conjugates using 200 mg of Ceritinib. Cer-OEG_2 is formed as oily solid (yield: 46%). ^{1}H-NMR (400 MHz, DMSO-d_6): δ= 9.53 (s, 1H), 8.57 (d, J = 8.4 Hz, 1H), 8.15 (s, 1H), 7.99 (s, 1H), 7.93 (dd, J = 8.0, 1.6 Hz, 1H), 7.67 – 7.56 (m, 2H), 6.73 (s, 1H), 4.64 – 4.49 (m, 1H), 4.21 – 4.11 (m, 4H), 3.74 (t, J = 5.0 Hz, 4H), 3.69 – 3.65 (m, 4H), 3.58 – 3.51 (m, 4H), 3.37 (s, 6H), 3.31 – 3.19 (m, 1H), 2.93 – 2.71 (m, 3H), 2.15 (s, 3H), 1.73 (d,

J = 13.0 Hz, 4H), 1.37 (d, J = 6.0 Hz, 6H), 1.32 (d, J = 6.9 Hz, 6H) ppm. ^{31}P-NMR (162 MHz, $CDCl_3$): δ= 9.35 ppm. HRMS (ESI$^+$) ($C_{38}H_{57}ClN_5NaO_{10}PS^+$ calc.: m/z 864.3144): [M+Na$^+$] m/z= 864.3172.

Synthesis of Cri-OEG$_2$

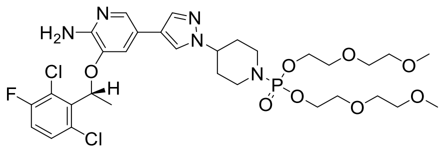

The reaction follows the general synthetic procedure for Drug-OEG conjugates using 200 mg of Crizotinib. Cri-OEG$_2$ is formed as oily solid (yield: 43%). ^{1}H-NMR (400 MHz, $CDCl_3$): δ= 7.75 (d, *J* = 1.8 Hz, 1H), 7.55 (d, *J* = 0.7 Hz, 1H), 7.50 (s, 1H), 7.30 (dd, *J* = 8.9, 4.8 Hz, 1H), 7.05 (dd, *J* = 8.9, 7.9 Hz, 1H), 6.87 (d, *J* = 1.8 Hz, 1H), 6.07 (q, *J* = 6.7 Hz, 1H), 4.21 (td, *J* = 7.5, 3.7 Hz, 1H), 4.15 (dt, *J* = 7.5, 4.9 Hz, 4H), 3.75 (s, 2H), 3.72 (dd, *J* = 5.9, 4.1 Hz, 4H), 3.69 – 3.62 (m, 4H), 3.57 – 3.50 (m, 4H), 3.35 (d, *J* = 0.6 Hz, 6H), 2.91 (q, *J* = 12.0 Hz, 2H), 2.13 (d, *J* = 12.9 Hz, 2H), 1.97 (td, *J* = 12.1, 4.2 Hz, 2H), 1.86 (d, *J* = 6.7 Hz, 3H) ppm. ^{31}P-NMR (243 MHz, $CDCl_3$): δ= 8.89 ppm. HRMS (ESI$^+$) ($C_{31}H_{43}Cl_2FN_5NaO_8P^+$ calc.: m/z 756.2103): [M+Na$^+$] m/z= 756.2135.

Synthesis of Pal-OEG$_2$

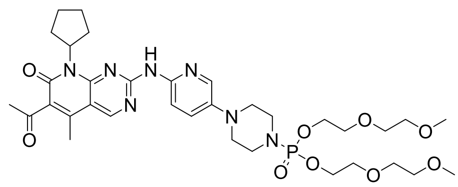

The reaction follows the general synthetic procedure for Drug-OEG conjugates using 200 mg of Palbociclib. Pal-OEG$_2$ is formed as oily solid (yield: 24%). ^{1}H-NMR (400 MHz, $CDCl_3$): δ= 8.84 (s, 1H), 8.18 (d, J = 9.1 Hz, 1H), 8.04 (d, J = 2.9 Hz, 1H), 7.32 (dd, J = 9.1, 3.0 Hz, 1H), 5.96 – 5.78 (m, 1H), 4.23 – 4.08 (m, 4H), 3.72 (dd, J = 5.7, 4.2 Hz, 4H), 3.68 – 3.62 (m, 4H), 3.57 – 3.51 (m, 4H), 3.37 (s, 10H), 3.13 (t, J = 4.8 Hz, 4H), 2.54 (s, 3H), 2.37 (s, 5H), 2.12 – 2.00 (m, 2H), 1.87 (q, J = 3.8 Hz, 2H), 1.69 (td, J = 5.5, 2.9 Hz, 2H) ppm. ^{31}P-NMR (243 MHz, $CDCl_3$): δ= 8.45 ppm. HRMS (ESI$^+$) ($C_{34}H_{50}N_7NaO_9P^+$ calc.: m/z 754.3300): [M+Na$^+$] m/z= 754.3328.

Synthesis of Rib-OEG$_2$

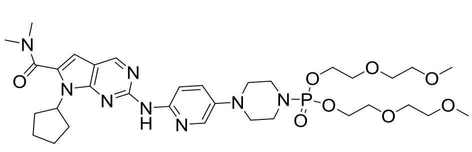

The reaction follows the general synthetic procedure for Drug-OEG conjugates using 200 mg of Ribociclib. Rib-OEG$_2$ is formed as oily solid (yield: 23%). ^{1}H-NMR (400 MHz, $CDCl_3$): δ= 8.69 (s, 1H), 8.36 (d, J = 9.0 Hz, 1H), 7.98 (d, J = 3.0 Hz, 1H), 7.31 (dd, J = 9.1, 3.0 Hz, 1H), 6.43 (s, 1H), 4.86 – 4.72 (m, 1H), 4.21 – 4.10 (m, 4H), 3.72 (dd, J = 5.7, 4.2 Hz, 4H), 3.68 – 3.63 (m, 4H), 3.57 – 3.52 (m, 4H), 3.37 (s, 10H), 3.15 (s, 6H), 3.09 (t, J = 4.9 Hz, 4H), 2.58 (t, J = 10.9 Hz, 2H), 2.12 – 1.99 (m, 4H), 1.72 (s, 2H) ppm. ^{31}P-NMR (243 MHz, $CDCl_3$): δ= 8.54 ppm. HRMS (ESI$^+$) ($C_{33}H_{51}N_8NaO_8P^+$ calc.: m/z 741.3460): [M+Na$^+$] m/z= 741.3484.

Synthesis of Cer-OEG$_3$

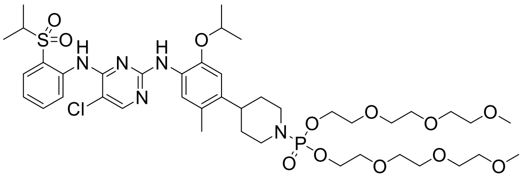

The reaction follows the general synthetic procedure for Drug-OEG conjugates using 200 mg of Ceritinib. Cer-OEG$_3$ is formed as oily solid (yield: 45%). ^{1}H-NMR (400 MHz, $CDCl_3$): δ= 9.55 (s, 1H), 8.57 (d, J = 8.4 Hz, 1H), 8.14 (s, 1H), 8.02 – 7.89 (m, 2H), 7.65 – 7.56 (m, 1H), 6.73 (s, 1H), 4.62 – 4.50 (m, 1H), 4.15 (dtd, J = 7.9, 4.9, 2.7 Hz, 4H), 3.73 – 3.63 (m, 16H), 3.57 – 3.50 (m, 4H), 3.37 (s, 6H), 3.31 – 3.20 (m, 1H), 2.91 – 2.72 (m, 3H), 2.15 (s, 3H), 1.73 (d, J = 13.3 Hz, 3H), 1.58 (d, J = 3.8 Hz, 3H), 1.37 (d, J = 6.1 Hz, 6H), 1.32 (d, J = 6.9 Hz, 6H) ppm. ^{31}P-NMR (162 MHz, $CDCl_3$): δ= 9.36 ppm. HRMS (ESI$^+$) ($C_{42}H_{65}ClN_5NaO_{12}PS^+$ calc.: m/z 952.3669): [M+Na$^+$] m/z= 952.3708.

Synthesis of Cri-OEG$_3$

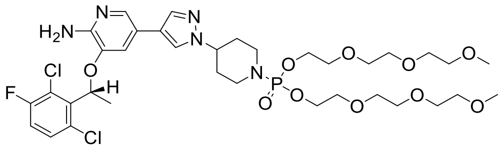

The reaction follows the general synthetic procedure for Drug-OEG conjugates using 200 mg of Crizotinib. Cri-OEG$_3$ is formed as oily solid (yield: 60%). ^{1}H-NMR (400 MHz, $CDCl_3$): δ= 7.70 (d, J = 1.7 Hz, 1H), 7.55 (d, J = 0.8 Hz, 1H), 7.50 (d, J = 0.8 Hz, 1H), 7.35 – 7.29 (m, 1H), 7.07 (dd, J = 8.9, 7.9 Hz, 1H), 6.90 (d, J = 1.8 Hz, 1H), 6.09 (q, J = 6.7 Hz, 1H), 4.26 – 4.17 (m, 1H), 4.17 – 4.10 (m, 4H), 3.74 (s, 1H), 3.73 – 3.69 (m, 4H), 3.69 – 3.60 (m, 12H), 3.55 – 3.49 (m, 4H), 3.35 (d, J = 0.5 Hz, 6H), 2.90 (q, J = 11.3 Hz, 2H), 2.20 – 2.04 (m, 2H), 1.97 (td, J = 12.1, 4.2 Hz, 1H), 1.87 (d, J = 6.7 Hz, 3H), 1.68 (s, 2H) ppm. ^{31}P-NMR (162 MHz, $CDCl_3$): δ= 8.89 ppm. HRMS (ESI$^+$) ($C_{35}H_{51}Cl_2FN_5NaO_{10}P^+$ calc.: m/z 844.2627): [M+Na$^+$] m/z= 844.2650.

Synthesis of Pal-OEG$_3$

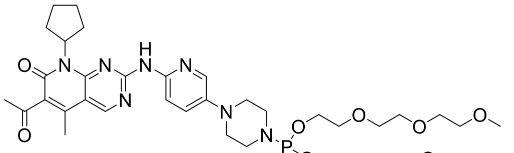

The reaction follows the general synthetic procedure for Drug-OEG conjugates using 200 mg of Palbociclib. Pal-OEG$_3$ is formed as oily solid (yield: 61%). ^{1}H-NMR (400 MHz, $CDCl_3$): δ= 8.79 (s, 1H), 8.18 (d, J = 9.0 Hz, 1H), 8.01 (d, J = 2.9 Hz, 1H), 7.90 (s, 1H), 7.33 (dd, J = 9.1, 3.0 Hz, 1H), 5.87 (t, J = 8.9 Hz, 1H), 4.15 (dt, J = 7.4, 4.9 Hz, 4H), 3.71 (q, J = 5.1 Hz, 4H), 3.66 (dtd, J = 13.0, 5.3, 3.4 Hz, 12H), 3.54 (dd, J = 5.8, 3.5 Hz, 4H), 3.37 (s, 10H), 3.13 (t, J = 5.0 Hz, 4H), 2.55 (s, 3H), 2.37 (s, 3H), 2.33 (s, 2H), 2.07 (s, 2H), 1.96 – 1.81 (m, 2H), 1.70 (t, J = 5.7 Hz, 2H) ppm. ^{31}P-NMR (162 MHz, $CDCl_3$): δ= 8.48 ppm. HRMS (ESI$^+$) ($C_{38}H_{58}N_7NaO_{11}P^+$ calc.: m/z 842.3825): [M+Na$^+$] m/z= 842.3876.

Synthesis of Rib-OEG$_3$

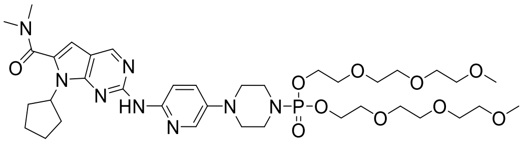

The reaction follows the general synthetic procedure for Drug-OEG conjugates using 200 mg of Ribociclib. Rib-OEG$_3$ is formed as oily solid (yield: 46%). ^{1}H-NMR (400 MHz, $CDCl_3$): δ= 8.68 (s, 1H), 8.37 (d, J = 9.1 Hz, 1H), 7.96 (d, J = 2.9 Hz, 1H), 7.33 (dd, J = 9.1, 3.0 Hz, 1H), 6.44 (s, 1H), 4.87 – 4.72 (m, 1H), 4.15 (dt, J = 7.4, 4.9 Hz, 4H), 3.75 – 3.69 (m, 4H), 3.68 – 3.60 (m, 12H), 3.56 – 3.51 (m, 4H), 3.37 (s, 10H), 3.16 (s, 6H), 3.09 (t, J = 5.0 Hz, 4H), 2.57 (d, J = 10.5 Hz, 2H), 2.12 – 2.01 (m, 4H), 1.72 (d, J = 6.4 Hz, 2H) ppm. ^{31}P-NMR (162 MHz, $CDCl_3$): δ= 8.54 ppm. HRMS (ESI$^+$) ($C_{37}H_{59}N_8NaO_{10}P^+$ calc.: m/z 829.3984): [M+Na$^+$] m/z= 829.4019.

Synthesis of Rib-OEG$_4$

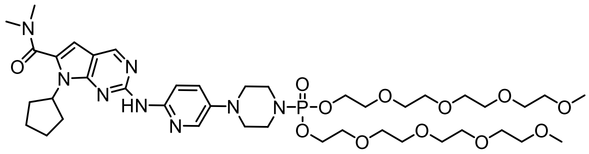

The reaction follows the general synthetic procedure for Drug-OEG conjugates using 20 mg of Ribociclib. Rib-OEG$_4$ is formed as oily solid (yield: 40%). ^{1}H-NMR (400 MHz, $CDCl_3$): δ= 8.68 (s, 1H), 8.36 (d, J = 9.1 Hz, 1H), 7.98 (d, J = 2.9 Hz, 1H), 7.31 (dd, J = 9.1, 3.0 Hz, 1H), 6.43 (s, 1H), 4.85 – 4.72 (m, 1H), 4.14 (dt, J = 7.5, 4.9 Hz, 4H), 3.71 (dd, J = 5.7, 4.2 Hz, 4H), 3.67 – 3.61 (m, 20H), 3.55 – 3.51 (m, 4H), 3.36 (s, 10H), 3.15 (s, 6H), 3.09 (t, J = 4.9 Hz, 4H), 2.58 (s, 2H), 2.07 (d, J = 12.7 Hz, 4H), 1.70 (s, 2H) ppm. ^{31}P-NMR (162 MHz, $CDCl_3$): δ= 8.58 ppm. HRMS (ESI$^+$) ($C_{41}H_{67}N_8NaO_{12}P^+$ calc.: m/z 917.4508): [M+Na$^+$] m/z= 917.4421

Synthesis of Cer-OEG$_8$

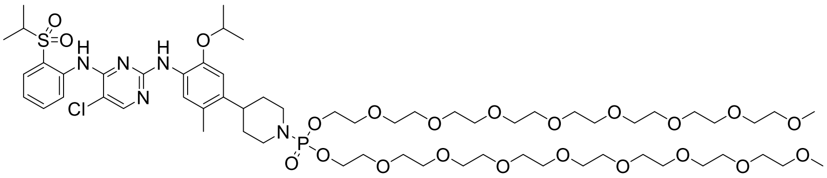

The reaction follows the general synthetic procedure for Drug-OEG conjugates using 50 mg of Ceritinib. Cer-OEG$_8$ is formed as oily solid (yield: 47%). ^{1}H-NMR (400 MHz, $CDCl_3$): δ= 9.50 (s, 1H), 8.58 (d, *J* = 8.2 Hz, 1H), 8.15 (s, 1H), 8.02 (s, 1H), 7.93 (dd, *J* = 8.0, 1.6 Hz, 1H), 7.67 – 7.58 (m, 1H), 7.53 (d, *J* = 10.1 Hz, 1H), 6.72 (s, 1H), 4.60 – 4.50 (m, 1H), 4.17 – 4.11 (m, 4H), 3.72 (t, *J* = 5.0 Hz, 4H), 3.67 – 3.61 (m, 52H), 3.56 – 3.52 (m, 4H), 3.37 (s, 6H), 3.30 – 3.22 (m, 1H), 2.91 – 2.70 (m, 3H), 2.15 (s, 3H), 1.73 (d, *J* = 12.9 Hz, 2H), 1.62 (s, 4H), 1.37 (d, *J* = 6.0 Hz, 6H), 1.32 (d, *J* = 6.9 Hz, 6H) ppm. ^{31}P-NMR (243 MHz, $CDCl_3$): δ= 9.38 ppm. HRMS (ESI$^+$) ($C_{62}H_{105}ClN_5NaO_{22}PS^+$ calc.: m/z 1392.6290): [M+Na$^+$] m/z= 1392.61932.

Synthesis of Cri-OEG$_8$

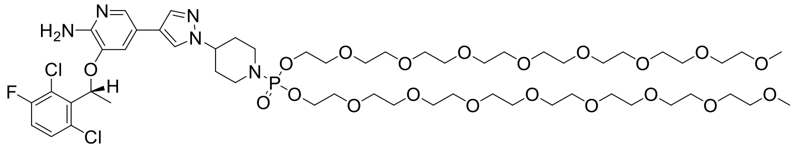

The reaction follows the general synthetic procedure for Drug-OEG conjugates using 50 mg of Crizotinib. Cri-OEG$_8$ is formed as oily solid (yield: 68%). ^{1}H-NMR (400 MHz, $CDCl_3$): δ= 7.74 (d, J = 1.8 Hz, 1H), 7.52 (dd, J = 17.7, 0.8 Hz, 2H), 7.31 (dd, J = 8.9, 4.8 Hz, 1H), 7.06 (dd, J = 8.9, 7.9 Hz, 1H), 6.86 (d, J = 1.8 Hz, 1H), 6.07 (q, J = 6.7 Hz, 1H), 4.80 (s, 2H), 4.19 (s, 1H), 4.13 (dt, J = 7.6, 2.6 Hz, 4H), 3.70 (dd, J = 5.7, 4.2 Hz, 4H), 3.66 – 3.59 (m, 53H), 3.56 – 3.52 (m, 4H), 3.37 (s, 6H), 2.95 – 2.84 (m, 2H), 2.12 (d, J = 12.7 Hz, 2H), 1.95 (dd, J = 12.1, 4.2 Hz, 2H), 1.84 (s, 3H) ppm. ^{31}P-NMR (243 MHz, $CDCl_3$): δ= 8.91 ppm. HRMS (ESI$^+$) ($C_{55}H_{91}Cl_2N_5NaO_{20}P^+$ calc.: m/z 1284.5249): [M+Na$^+$] m/z= 1284.51661.

Synthesis of Pal-OEG$_8$

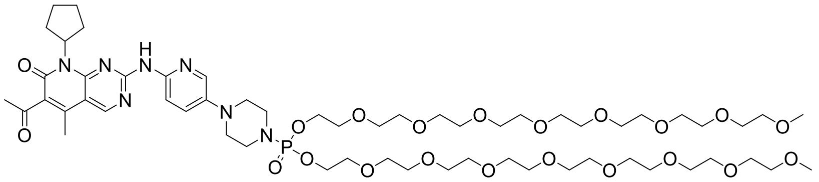

The reaction follows the general synthetic procedure for Drug-OEG conjugates using 50 mg of Palbociclib. Pal-OEG$_8$ is formed as oily solid (yield: 79%). ^{1}H-NMR (400 MHz, $CDCl_3$): δ= 8.79 (s, 1H), 8.18 (d, *J* = 9.0 Hz, 1H), 8.01 (d, *J* = 3.0 Hz, 1H), 7.87 (s, 1H), 7.33 (dd, *J* = 9.1, 3.0 Hz, 1H), 5.92 – 5.82 (m, 1H), 4.14 (dt, *J* = 7.5, 4.9 Hz, 4H), 3.71 (dd, *J* = 5.7, 4.3 Hz, 4H), 3.67 – 3.61 (m, 52H), 3.57 – 3.52 (m, 4H), 3.37 (s, 10H), 3.13 (t, *J* = 5.0 Hz, 4H), 2.55 (s, 3H), 2.37 (s, 3H), 2.34 (d, *J* = 7.6 Hz, 2H), 2.07 (s, 2H), 1.88 (d, *J* = 6.4 Hz, 2H), 1.70 (t, *J* = 5.7 Hz, 2H) ppm. ^{31}P-NMR (243 MHz, $CDCl_3$): δ= 8.49 ppm. HRMS (ESI$^+$) ($C_{58}H_{99}N_7NaO_{21}P^+$ calc.: m/z 1260.6627): [M+Na$^+$] m/z= 1260.66438.

Synthesis of Rib-OEG$_8$

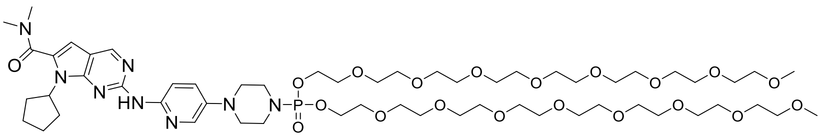

The reaction follows the general synthetic procedure for Drug-OEG conjugates using 45 mg of Ribociclib. Rib-OEG$_8$ is formed as oily solid (yield: 45%). ^{1}H-NMR (400 MHz, $CDCl_3$): δ= 8.68 (s, 1H), 8.36 (d, J = 9.1 Hz, 1H), 7.97 (d, J = 2.9 Hz, 1H), 7.73 (s, 1H), 7.31 (dd, J = 9.1, 3.0 Hz, 1H), 6.44 (s, 1H), 4.85 – 4.73 (m, 1H), 4.14 (dt, J = 7.6, 5.0 Hz, 4H), 3.71 (dd, J = 5.8, 4.3 Hz, 4H), 3.64 (q, J = 3.7 Hz, 52H), 3.58 – 3.51 (m, 4H), 3.37 (s, 6H), 3.37 – 3.32 (m, 4H), 3.16 (s, 6H), 3.09 (t, J = 4.9 Hz, 4H), 2.57 (d, J = 10.4 Hz, 2H), 2.07 (d, J = 11.8 Hz, 4H), 1.72 (d, J = 6.5 Hz, 2H) ppm. ^{31}P-NMR (243 MHz, $CDCl_3$): δ= 8.75 ppm. HRMS (ESI$^+$) ($C_{57}H_{99}N_8NaO_{20}P^+$ calc.: m/z 1269.6606): [M+Na$^+$] m/z= 1269.65366.

Water Solubility

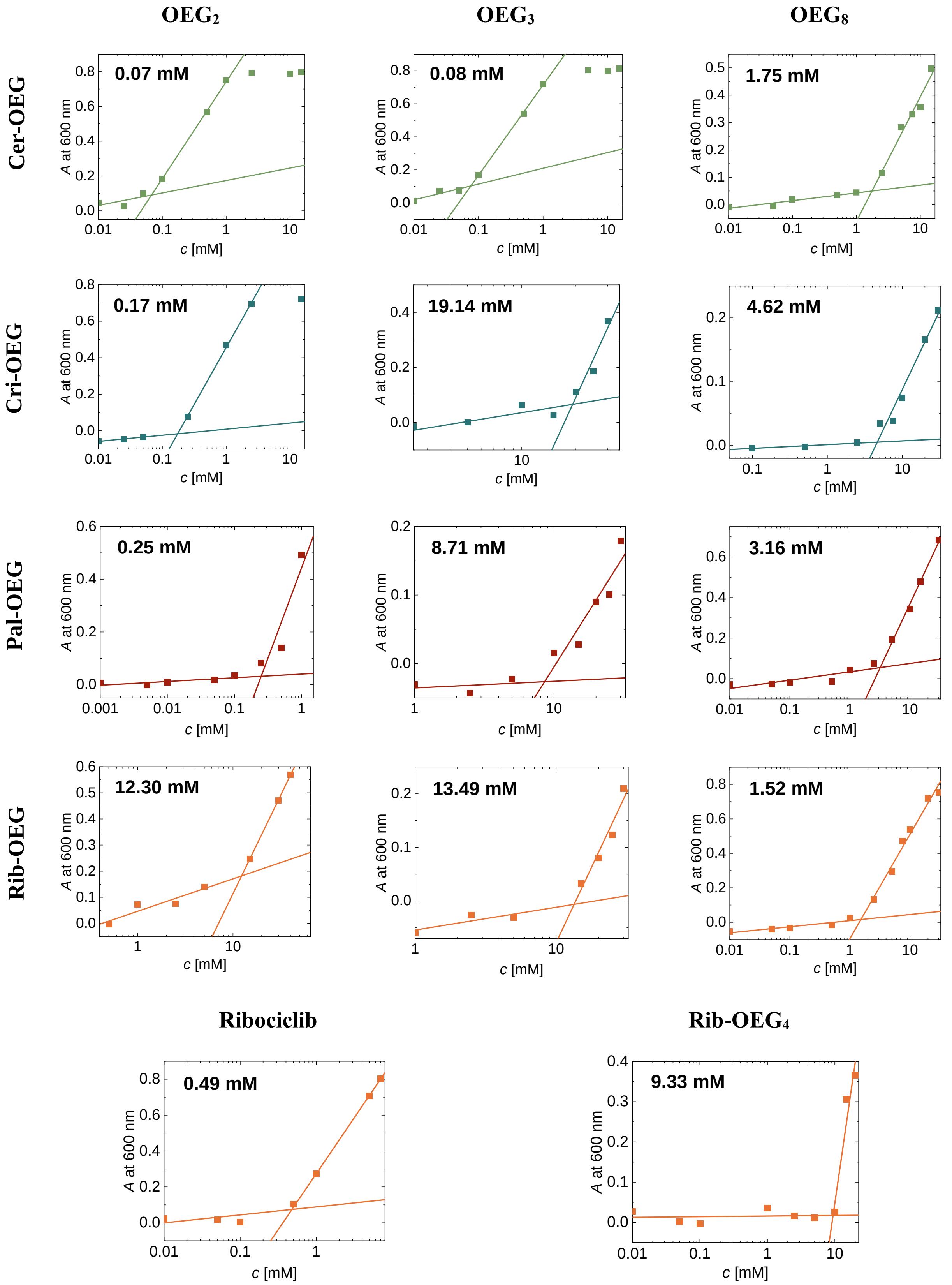

Figure S1: Water solubility determined by UV-Vis spectroscopy. Plotted absorption (*A*) at 600 nm against different concentrations (*c*). Ceritinib-conjugates are shown in green, Crizotinib-conjugates are shown in blue, Palbociclib-conjugates are shown in red and Ribociclib and Ribociclib-conjugates are shown in orange.

Dynamic Light Scattering (DLS)

Cer-OEG$_2$

Cer-OEG$_3$

Cer-OEG$_8$

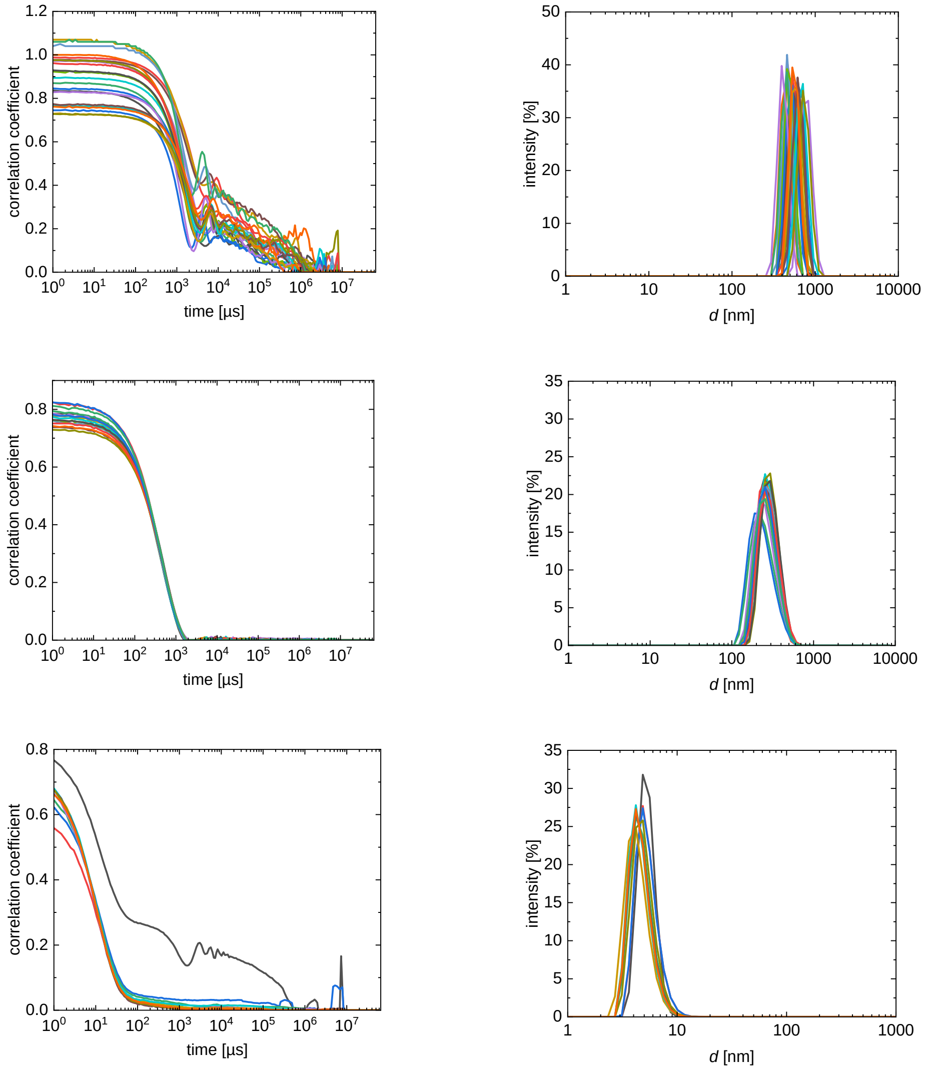

Figure S2: Correlation coefficient and hydrodynamic radius of Ceritinib prodrugs determined by DLS in water. **Cer-OEG$_2$** (0.07 mM) with a hydrodynamic radius of 565 nm (PDI 0.46). **Cer-OEG$_3$** (0.08 mM) with a hydrodynamic radius of 279 nm (PDI 0.05). **Cer-OEG$_8$** (1.75 mM) with a hydrodynamic radius of 4.9 nm (PDI 0.25).

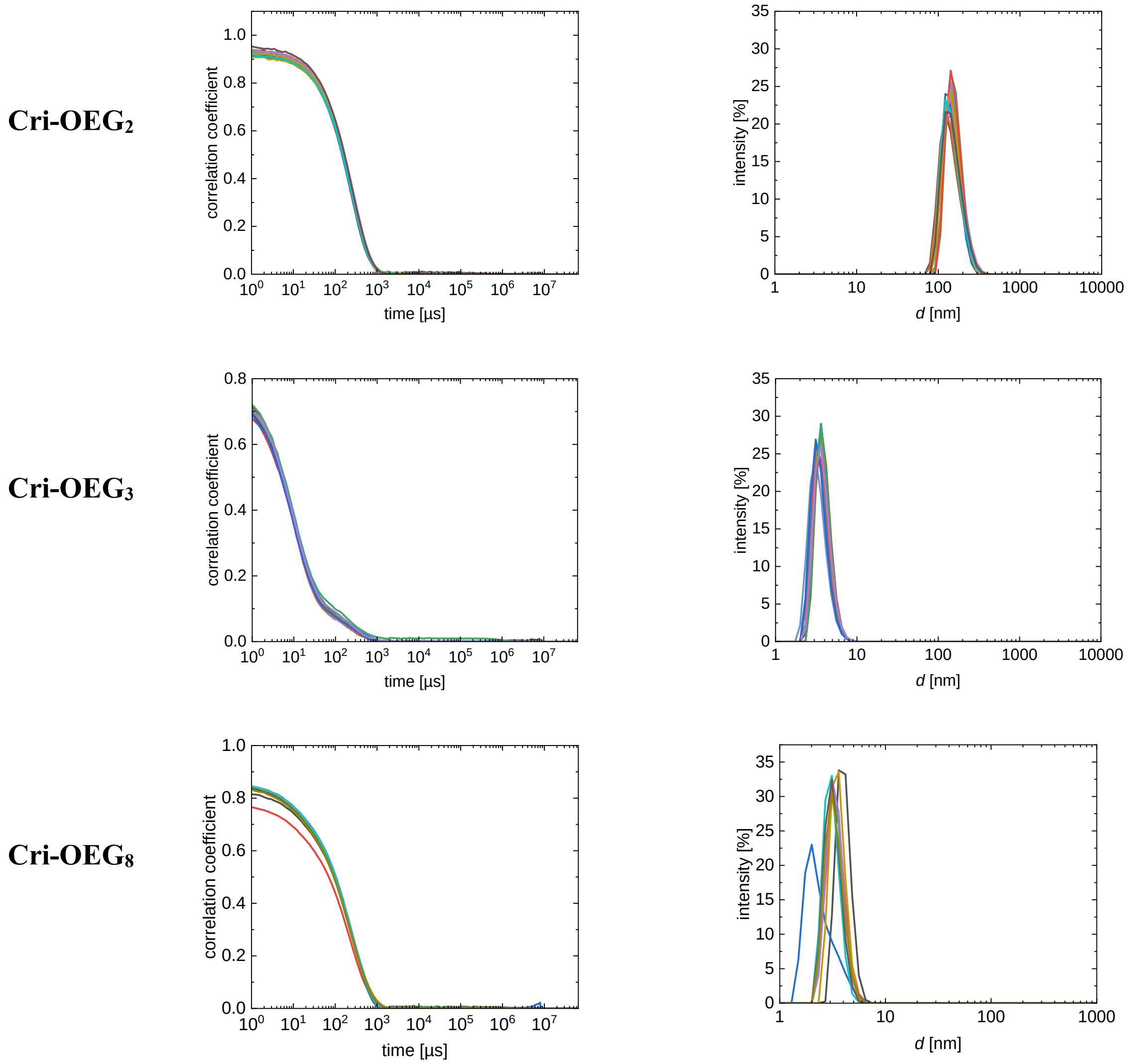

Figure S3: Correlation coefficient and hydrodynamic radius of Crizotinib prodrugs determined by DLS in water. **Cri-OEG$_2$** (0.17 mM) with a hydrodynamic radius of 151 nm (PDI 0.03). **Cri-OEG$_3$** (19.14 mM) with a hydrodynamic radius of 3.7 nm (PDI 0.02). **Cri-OEG$_8$** (4.62 mM) with a hydrodynamic radius of 3.3 nm (PDI 0.36).

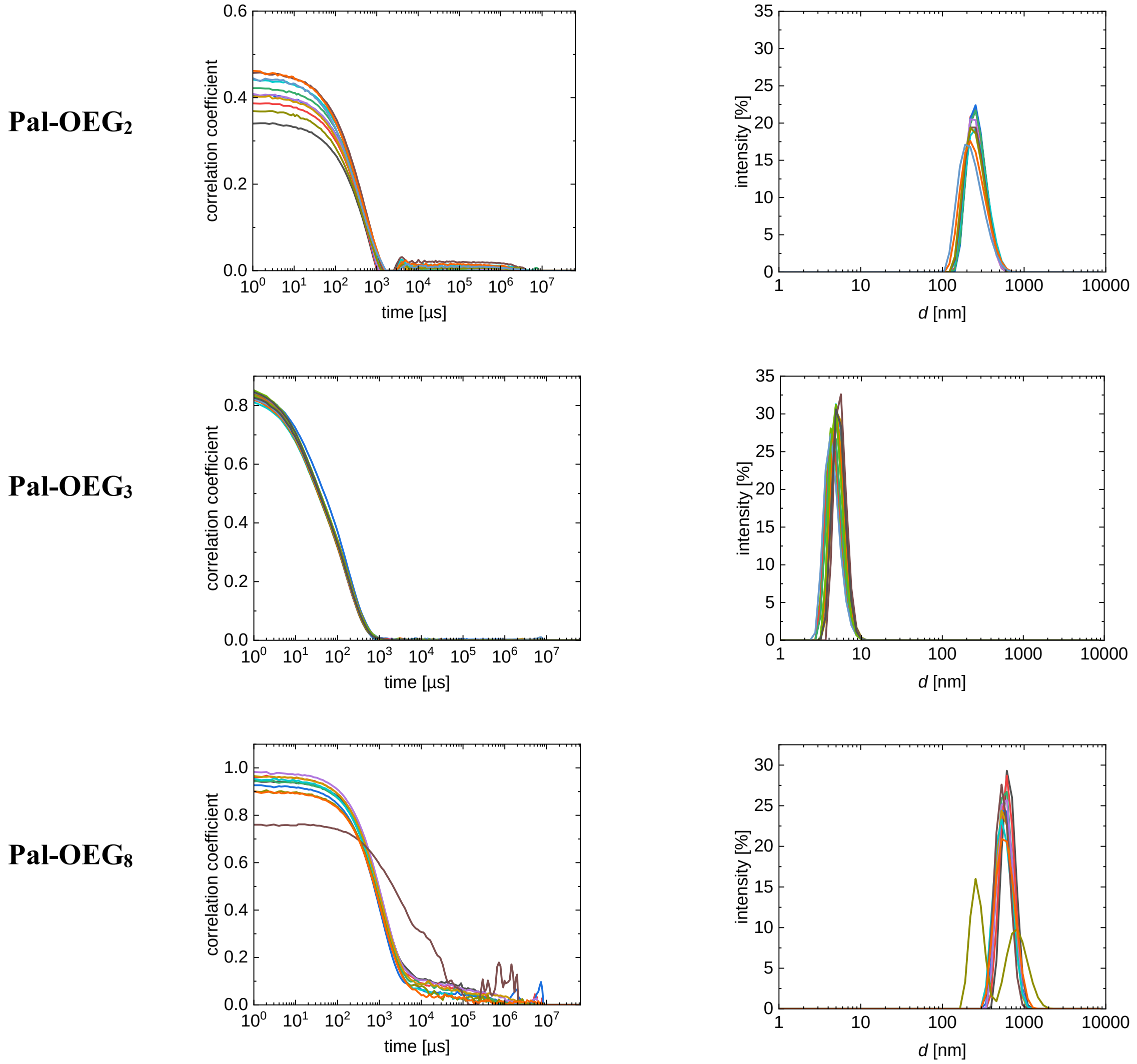

Figure S4: Correlation coefficient and hydrodynamic radius of Palbociclib prodrugs determined by DLS in water. **Pal-OEG$_2$** (0.25 mM) with a hydrodynamic radius of 264 nm (PDI 0.17). **Pal-OEG$_3$** (8.71 mM) with a hydrodynamic radius of 5 nm (PDI 0.74). **Pal-OEG$_8$** (3.75 mM) with a hydrodynamic radius of 596 nm (PDI 0.33).

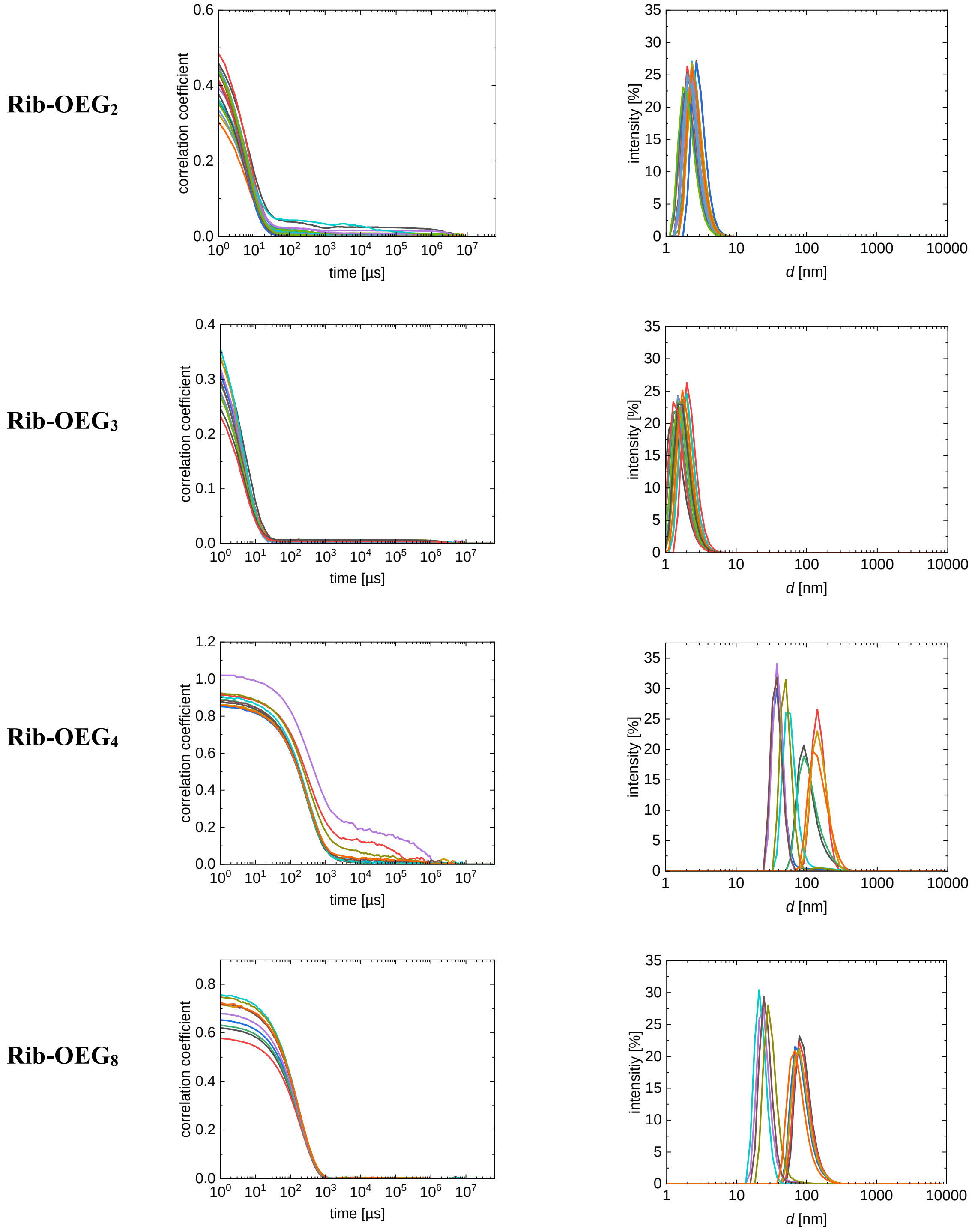

Figure S5: Correlation coefficient and hydrodynamic radius of Ribociclib prodrugs determined by DLS in water. **Rib-OEG$_2$** (12.3 mM) with a hydrodynamic radius of 2.5 nm (PDI 0.22). **Rib-OEG$_3$** (13.5 mM) with a hydrodynamic radius of 1.8 nm (PDI 0.15). **Rib-OEG$_4$** (10 mM) with a hydrodynamic radius of 92 nm (PDI 0.35). **Rib-OEG$_8$** (1.1 mM) with a hydrodynamic radius of 64 nm (PDI 0.22).

Cell-free Binding Assays

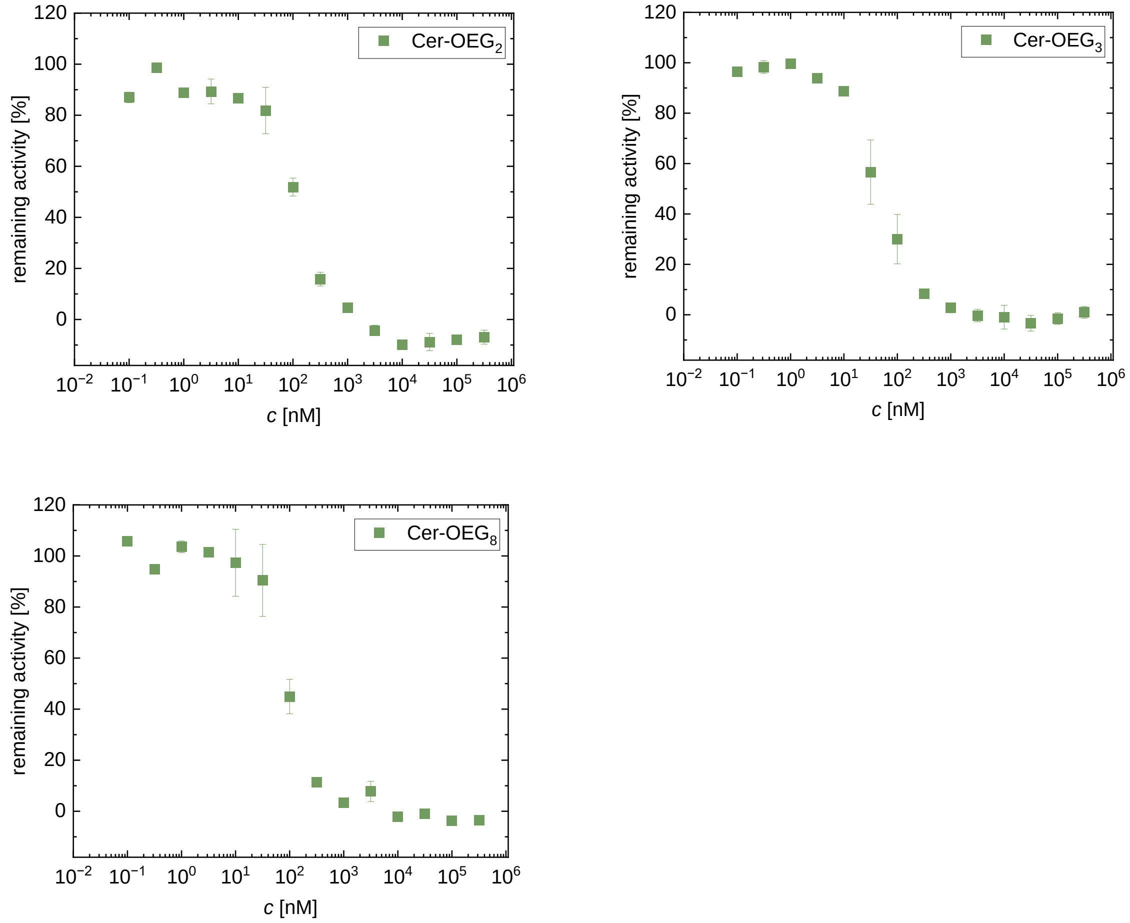

Figure S6: ROS protooncogene 1 (ROS1) cell-free inhibitor assay with drug-conjugates (n= 2,3 and 8) (green). Half maximal inhibitory concentration (IC_{50}) of **Cer-OEG$_2$**= 100 nM, **Cer-OEG$_3$**= 45 nM and **Cer-OEG$_8$**= 100 nM.

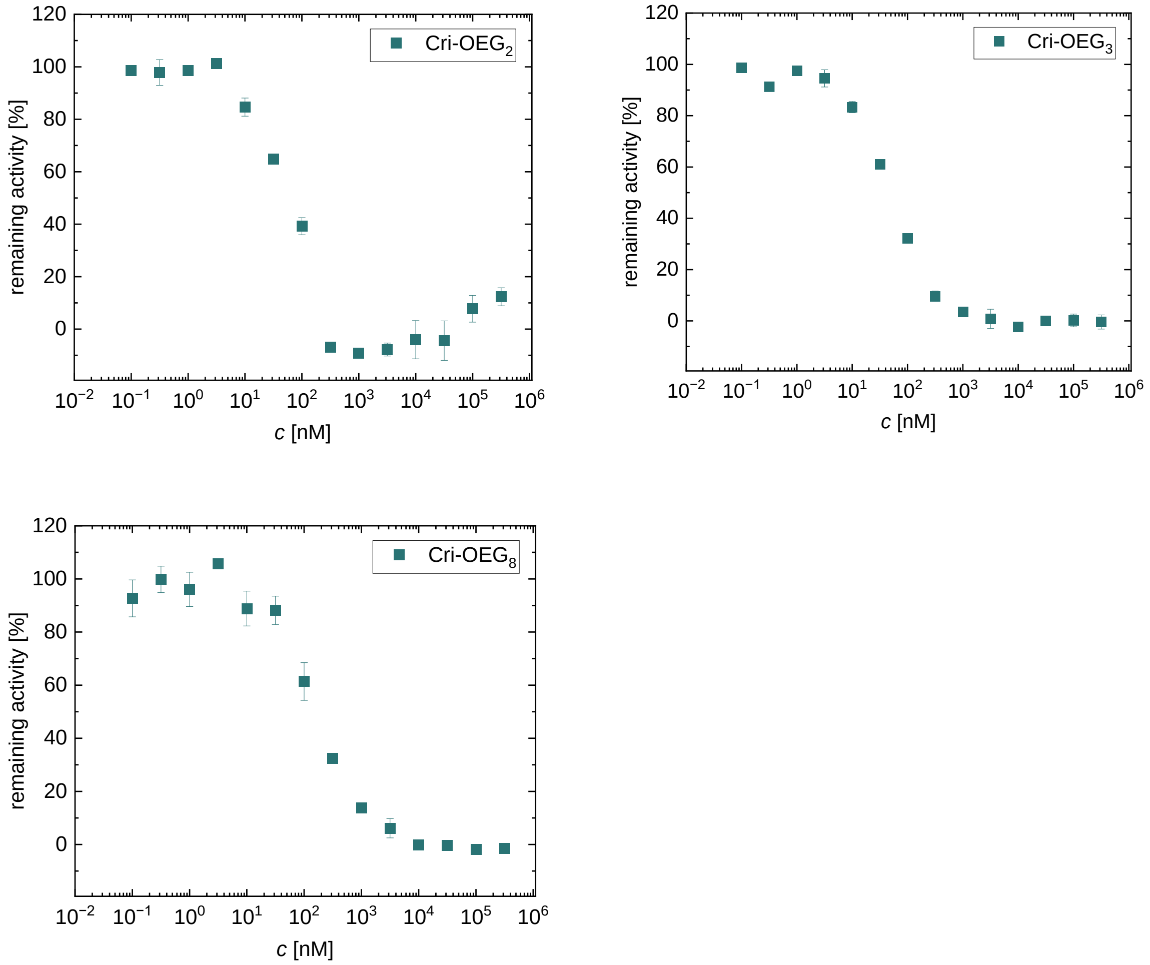

Figure S7: ROS protooncogene 1 (ROS1) cell-free inhibitor assay with drug-conjugates (n= 2,3 and 8) (blue). Half maximal inhibitory concentration (IC_{50}) of **Cri-OEG$_2$**= 50 nM, **Cri-OEG$_3$**= 45 nM and **Cri-OEG$_8$**= 140 nM.

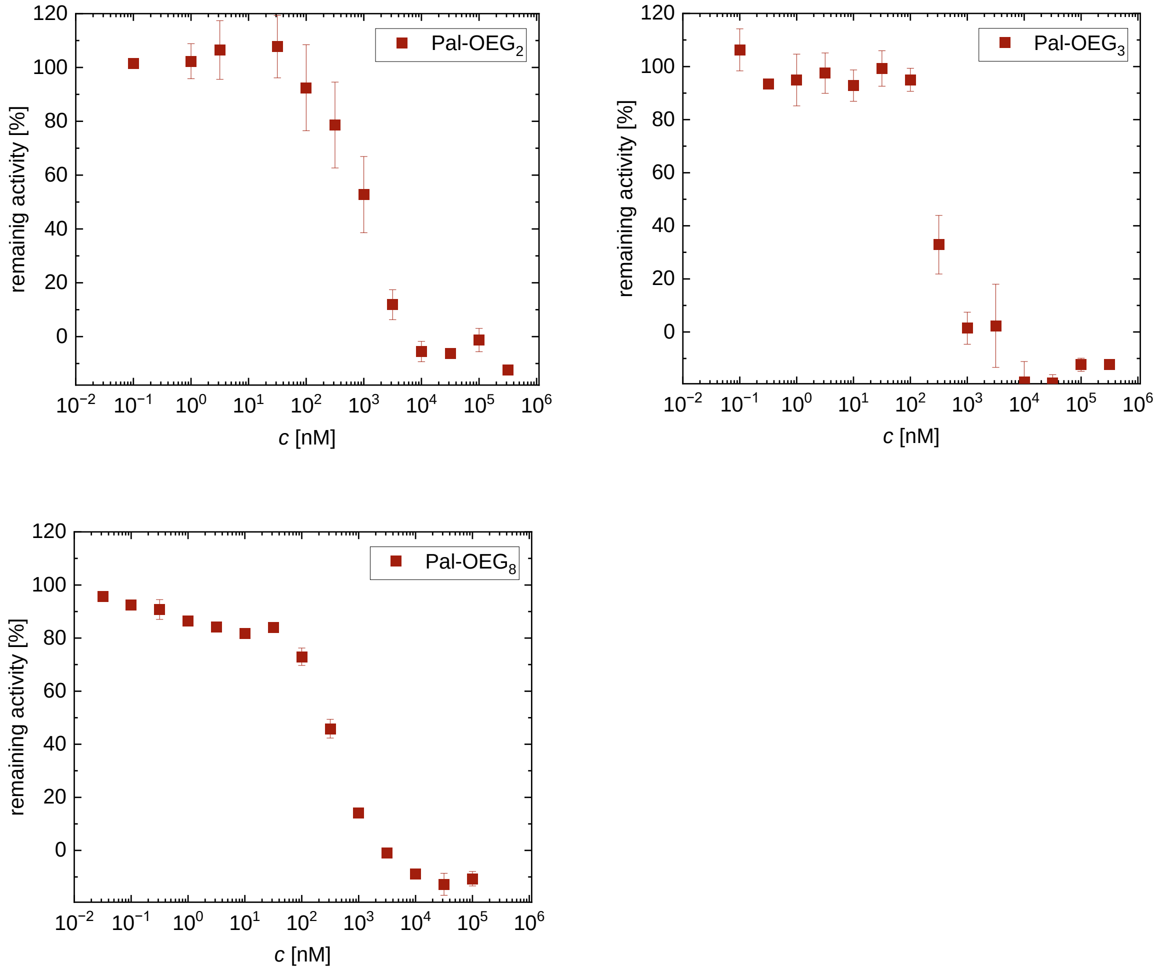

Figure S8: Cyclin-dependent kinase 4 (CDK4) cell-free inhibitor assay with drug-conjugates (n= 2,3 and 8) (red). Half maximal inhibitory concentration (IC_{50}) of **Pal-OEG$_2$**= 1000 nM, **Pal-OEG$_3$**= 215 nM and **Pal-OEG$_8$**= 270 nM.

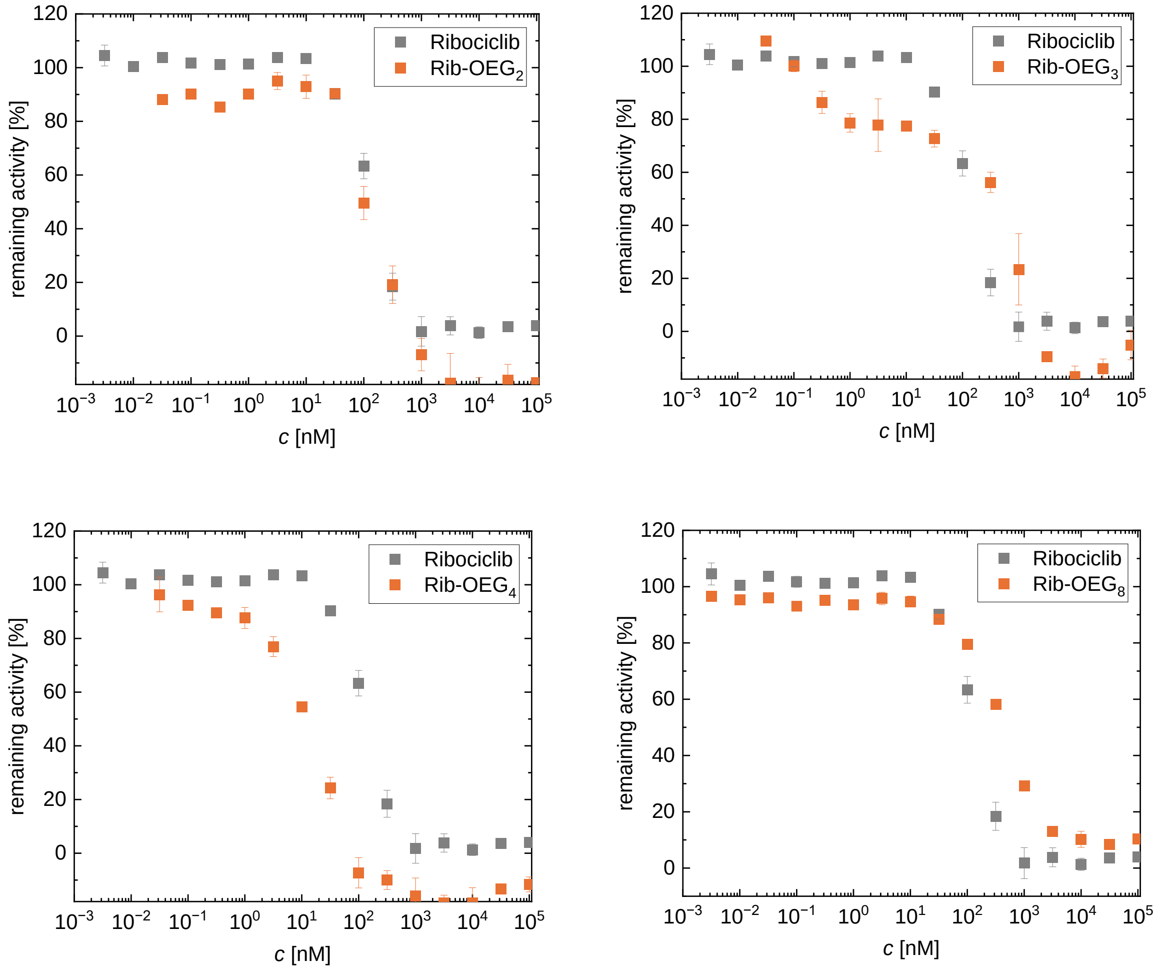

Figure S9: Cyclin-dependent kinase 4 (CDK4) cell-free inhibitor assay with Ribociclib (grey) and drug-conjugates (n= 2,3,4 and 8) (orange). Half maximal inhibitory concentration (IC_{50}) of Ribociclib= 120 nM, **Rib-OEG$_2$**= 110 nM, **Rib-OEG$_3$**= 370 nM, **Rib-OEG$_4$**= 12 nM and **Rib-OEG$_8$**= 400 nM.

pH Dependent Hydrolysis

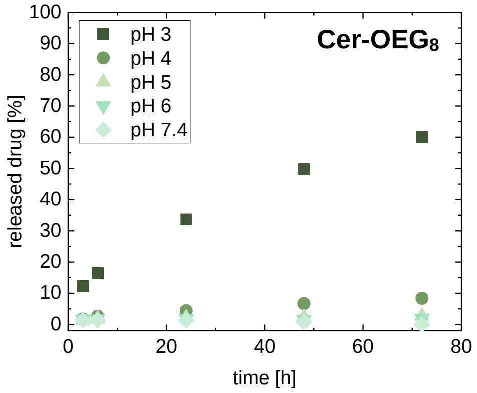

Figure S10: Ceritinib release determined from HPLC data after various times (0-96 h). **Cer-OEG$_2$** and **-OEG$_3$** are excluded from the study since the don´t dissolve properly.

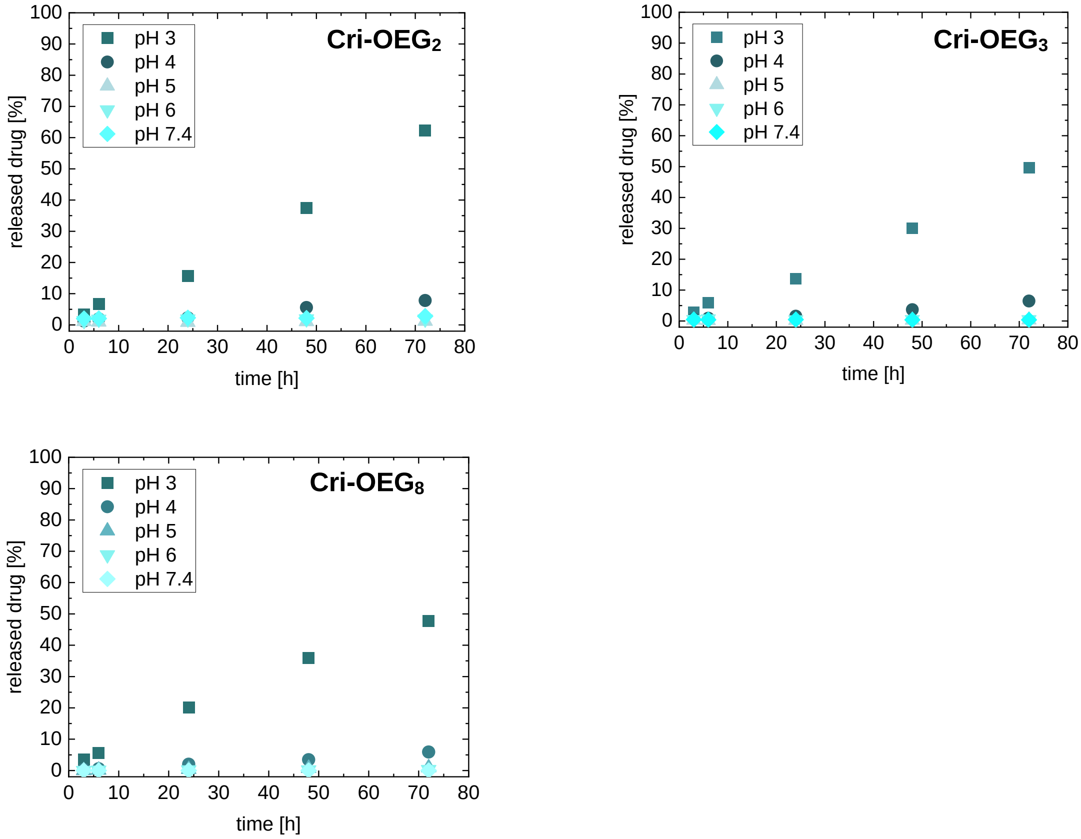

Figure S11: Crizotinib release determined from HPLC data after various times (0-96 h).

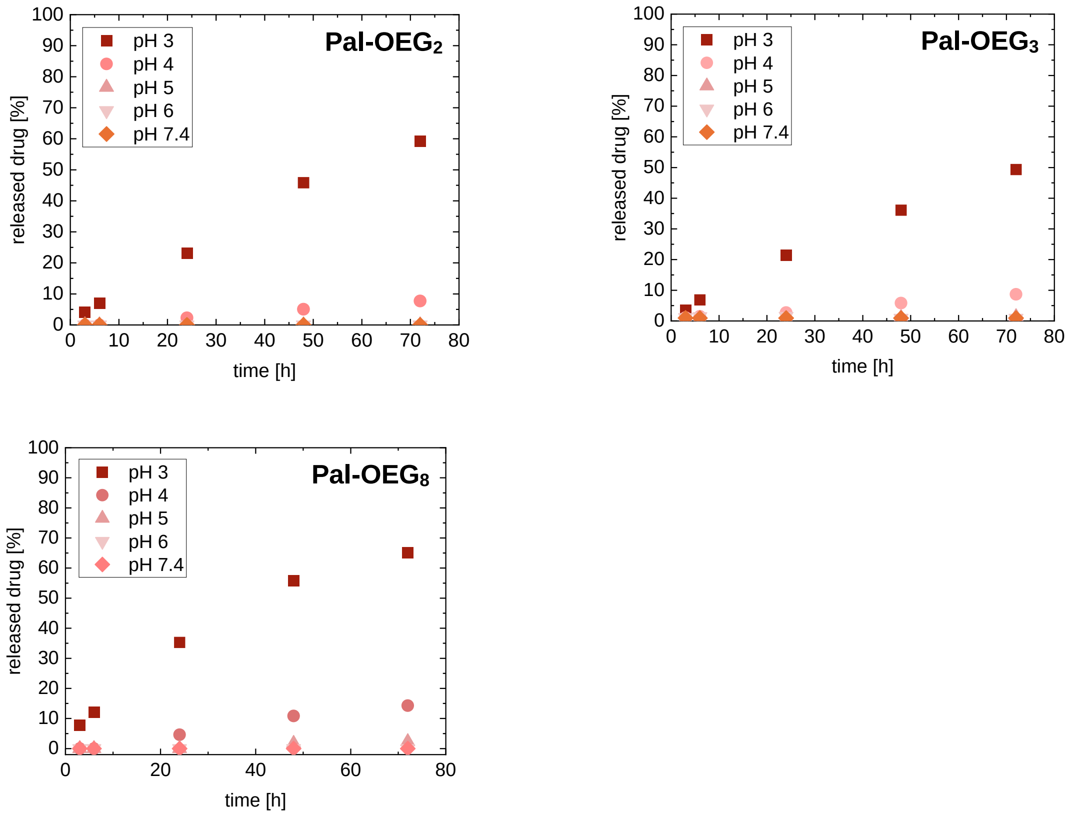

Figure S12: Palbociclib release determined from HPLC data after various times (0-96 h).

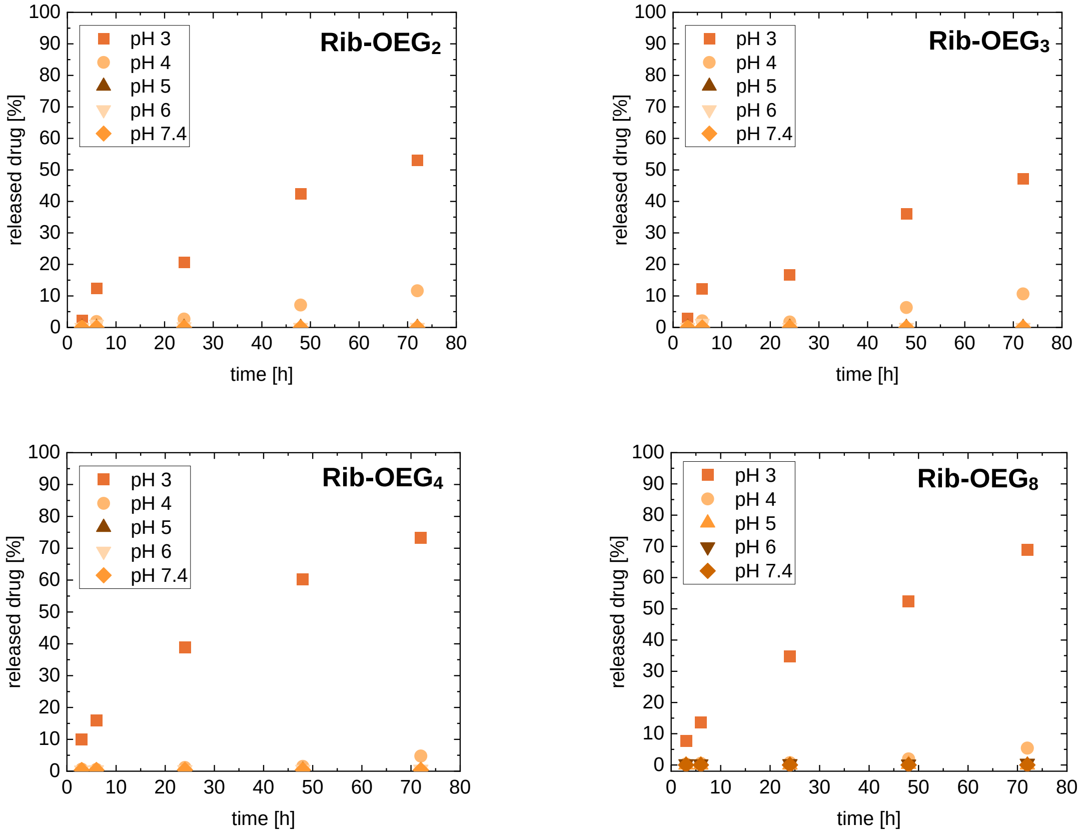

Figure S13: Ribociclib release determined from HPLC data after various times (0-96 h).

Table S1: Detailed values of pH dependent degradation of **Cri-OEG$_2$**. The drug release is calculated with the peak area (mAU*s) of the drug peak divided by the sum of the conjugate and drug peak.

Sample	pH	time [h]	Drug release [%]
**Cri-OEG$_2$**	3	3	3.38
		6	6.73
		24	15.75
		48	37.50
		72	62.32
	4	3	1.22
		6	1.93
		24	2.26
		48	5.56
		72	7.83
	5	3	1.09
		6	0.91
		24	0.84
		48	1.06
		72	1.17
	6	3	1.38
		6	1.81
		24	1.90
		48	1.94
		72	1.87
	7.4	3	2.00
		6	2.05
		24	2.27
		48	2.15
		72	2.80

Table S2: Detailed values of pH dependent degradation of **Pal-OEG$_2$**. The drug release is calculated with the peak area (mAU*s) of the drug peak divided by the sum of the conjugate and drug peak.

Sample	pH	time [h]	Drug release [%]
**Pal-OEG$_2$**	3	3	4.08
		6	7.00
		24	23.07
		48	45.86
		72	59.23
	4	3	0
		6	0
		24	2.28
		48	5.07
		72	7.72
	5	3	0
		6	0
		24	0
		48	0
		72	0
	6	3	0
		6	0
		24	0
		48	0
		72	0
	7.4	3	0
		6	0
		24	0
		48	0
		72	0

Table S3: Detailed values of pH dependent degradation of **Rib-OEG$_2$**. The drug release is calculated with the peak area (mAU*s) of the drug peak divided by the sum of the conjugate and drug peak.

Sample	pH	time [h]	Drug release [%]
**Rib-OEG$_2$**	3	3	2.17
		6	12.39
		24	20.70
		48	42.42
		72	53.04
	4	3	0
		6	1.88
		24	2.66
		48	7.11
		72	11.63
	5	3	0
		6	0
		24	0
		48	0
		72	0
	6	3	0
		6	1.01
		24	0.20
		48	0
		72	0
	7.4	3	0
		6	0
		24	0
		48	0
		72	0

Table S4: Detailed values of pH dependent degradation of **Cri-OEG$_3$**. The drug release is calculated with the peak area (mAU*s) of the drug peak divided by the sum of the conjugate and drug peak.

Sample	pH	time [h]	Drug release [%]
**Cri-OEG$_3$**	3	3	2.87
		6	5.90
		24	13.65
		48	30.06
		72	49.65
	4	3	0.64
		6	0.89
		24	1.55
		48	3.67
		72	6.46
	5	3	0.24
		6	0.17
		24	0.19
		48	0.26
		72	0.34
	6	3	0.44
		6	0.68
		24	0.36
		48	0.44
		72	0.42
	7.4	3	0.44
		6	0.40
		24	0.46
		48	0.38
		72	0.37

Table S5: Detailed values of pH dependent degradation of **Pal-OEG$_3$**. The drug release is calculated with the peak area (mAU*s) of the drug peak divided by the sum of the conjugate and drug peak.

Sample	pH	time [h]	Drug release [%]
Pal-OEG$_3$	3	3	3.50
		6	6.88
		24	21.37
		48	36.11
		72	49.35
	4	3	1.09
		6	1.53
		24	2.74
		48	5.83
		72	8.68
	5	3	0.72
		6	0.65
		24	0.75
		48	1.11
		72	1.38
	6	3	1.09
		6	1.41
		24	1.51
		48	0.89
		72	0.89
	7.4	3	0.97
		6	0.93
		24	0.93
		48	0.93
		72	0.94

Table S6: Detailed values of pH dependent degradation of **Rib-OEG$_3$**. The drug release is calculated with the peak area (mAU*s) of the drug peak divided by the sum of the conjugate and drug peak.

Sample	pH	time [h]	Drug release [%]
**Rib-OEG$_3$**	3	3	2.80
		6	12.23
		24	16.73
		48	36.00
		72	47.12
	4	3	0
		6	2.10
		24	1.72
		48	6.31
		72	10.63
	5	3	0
		6	0
		24	0
		48	0
		72	0
	6	3	0
		6	1.26
		24	0
		48	0
		72	0
	7.4	3	0
		6	0
		24	0
		48	0
		72	0

Table S7: Detailed values of pH dependent degradation of **Rib-OEG$_4$**. The drug release is calculated with the peak area (mAU*s) of the drug peak divided by the sum of the conjugate and drug peak.

Sample	pH	time [h]	Drug release [%]
Rib-OEG$_4$	3	3	9.95
		6	16.00
		24	38.92
		48	60.27
		72	73.30
	4	3	0.62
		6	0.53
		24	1.17
		48	1.5
		72	4.77
	5	3	0
		6	0
		24	0
		48	0.28
		72	0.21
	6	3	0.34
		6	0.42
		24	0.46
		48	0.56
		72	0.63
	7.4	3	0.34
		6	0.37
		24	0.40
		48	0.40
		72	0.39

Table S8: Detailed values of pH dependent degradation of **Cer-OEG$_8$**. The drug release is calculated with the peak area (mAU*s) of the drug peak divided by the sum of the conjugate and drug peak.

Sample	pH	time [h]	Drug release [%]
Cer-OEG$_8$	3	3	12.28
		6	16.48
		24	33.67
		48	49.84
		72	60.20
	4	3	1.74
		6	2.66
		24	4.38
		48	6.70
		72	8.39
	5	3	1.31
		6	1.85
		24	2.19
		48	2.31
		72	2.67
	6	3	1.71
		6	1.75
		24	1.86
		48	1.95
		72	1.71
	7.4	3	1.41
		6	1.39
		24	1.41
		48	0.75
		72	0

Table S9: Detailed values of pH dependent degradation of **Cri-OEG$_8$**. The drug release is calculated with the peak area (mAU*s) of the drug peak divided by the sum of the conjugate and drug peak.

Sample	pH	time [h]	Drug release [%]
Cri-OEG$_8$	3	3	3.60
		6	5.65
		24	20.19
		48	35.96
		72	47.79
	4	3	0.36
		6	0.56
		24	2.00
		48	3.46
		72	5.92
	5	3	0
		6	0.13
		24	0.38
		48	0.72
		72	0.91
	6	3	0
		6	0.11
		24	0.18
		48	0.20
		72	0.32
	7.4	3	0
		6	0
		24	0
		48	0
		72	0

Table S10: Detailed values of pH dependent degradation of **Pal-OEG$_8$**. The drug release is calculated with the peak area (mAU*s) of the drug peak divided by the sum of the conjugate and drug peak.

Sample	pH	time [h]	Drug release [%]
**Pal-OEG$_8$**	3	3	7.76
		6	12.13
		24	35.29
		48	55.73
		72	65.09
	4	3	0
		6	0
		24	4.62
		48	10.85
		72	14.29
	5	3	0
		6	0
		24	0
		48	1.74
		72	2.30
	6	3	0
		6	0
		24	0
		48	0
		72	0
	7.4	3	0
		6	0
		24	0
		48	0
		72	0

Table S11: Detailed values of pH dependent degradation of **Rib-OEG$_8$**. The drug release is calculated with the peak area (mAU*s) of the drug peak divided by the sum of the conjugate and drug peak.

Sample	pH	time [h]	Drug release [%]
**Rib-OEG$_8$**	3	3	7.65
		6	13.72
		24	34.73
		48	52.39
		72	68.84
	4	3	0.32
		6	0.50
		24	0.71
		48	1.94
		72	5.40
	5	3	0
		6	0
		24	0
		48	0.18
		72	0
	6	3	0.37
		6	0.33
		24	0.41
		48	0.27
		72	0.53
	7.4	3	0
		6	0
		24	0
		48	0
		72	0

Cell Uptake with Confocal Laser Scanning Microscopy (CLSM)

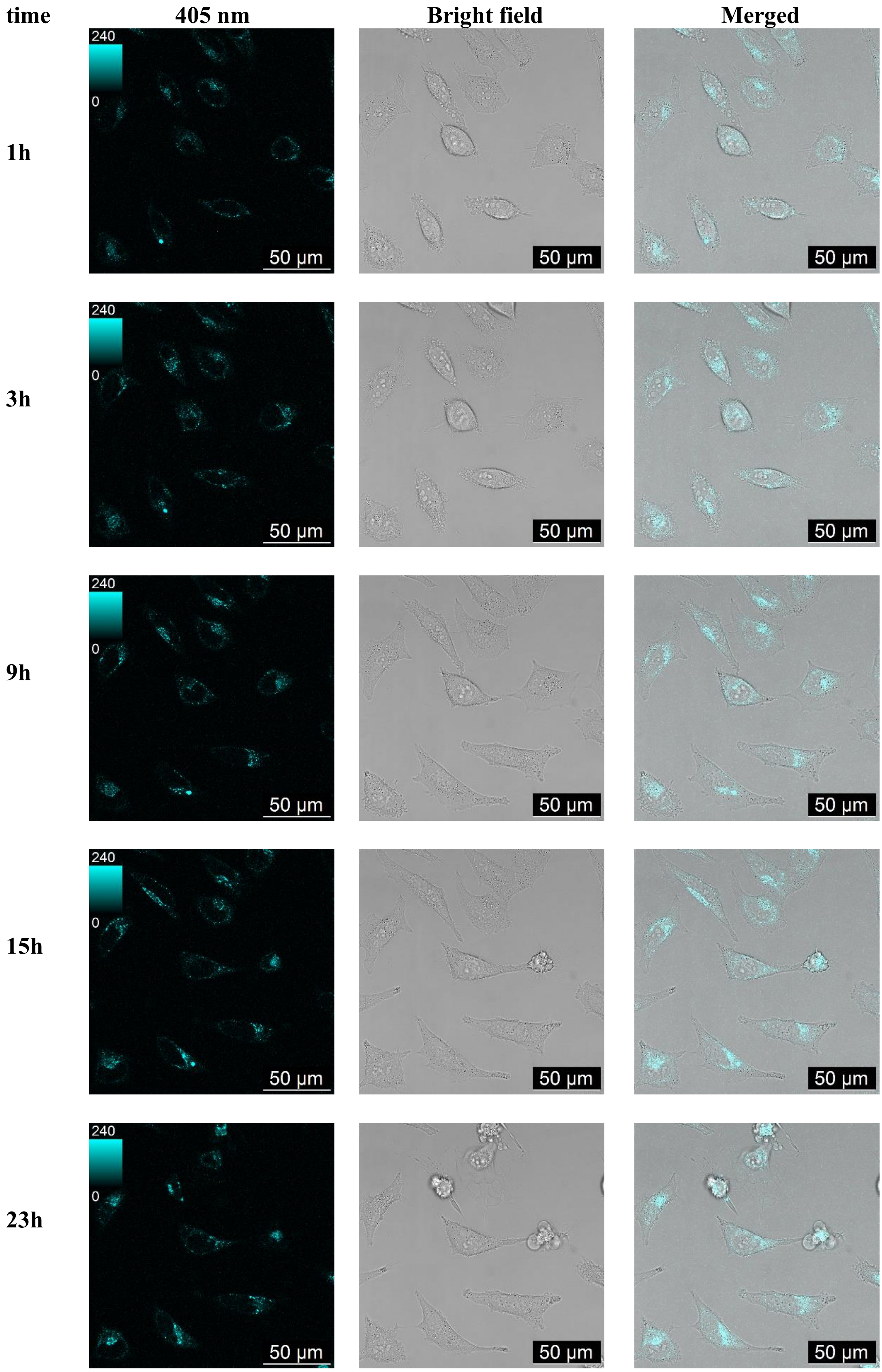

Figure S14: CLSM of **Ribociclib** (25 µM) in HeLa cells after different time points of incubation. Drug (shown in blue) is excited at 405 nm. Cells are visible in bright field.

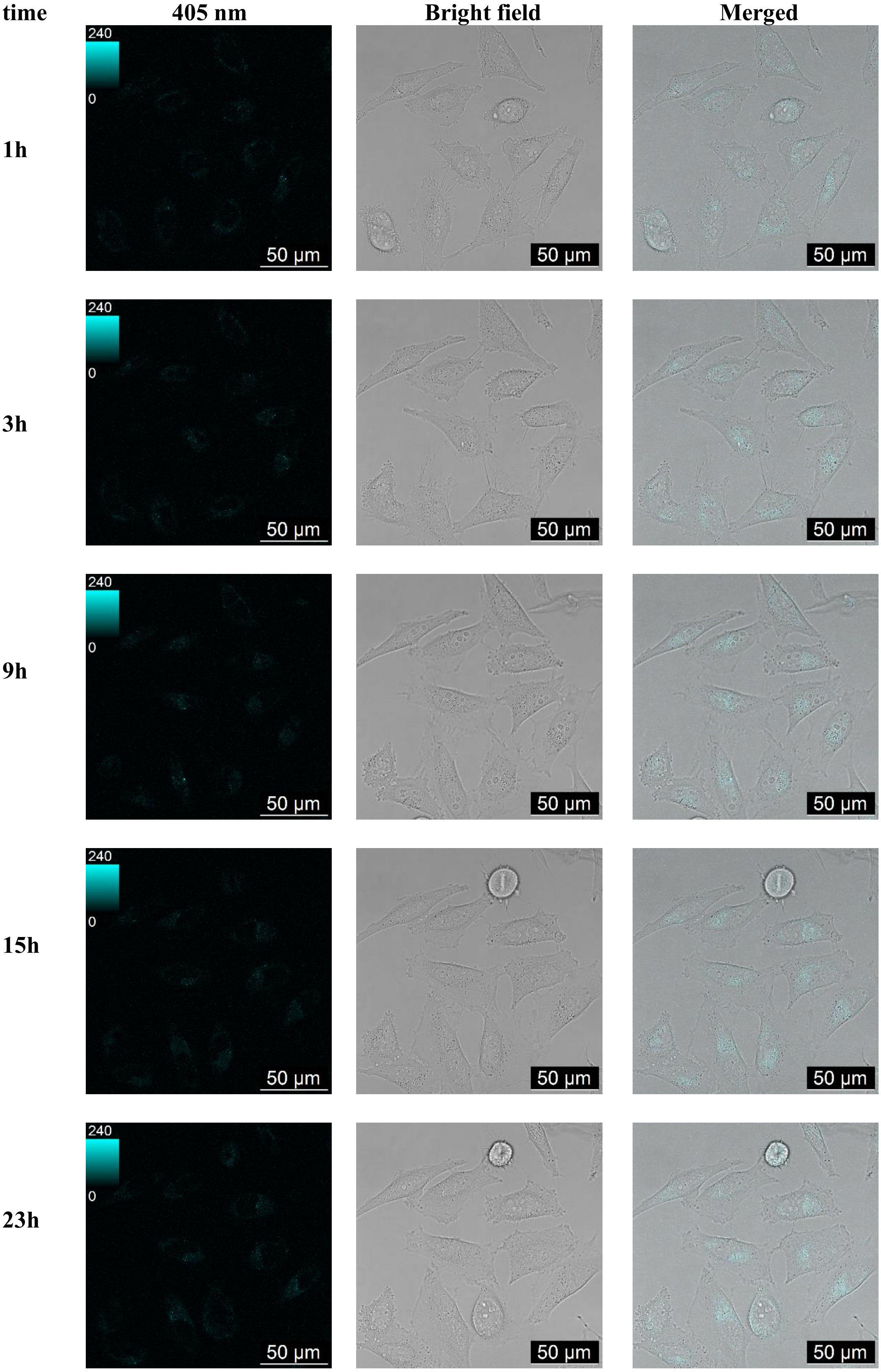

Figure S15: CLSM of **Rib-OEG$_4$** (25 µM) in HeLa cells after different time points of incubation. Drug (shown in blue) is excited at 405 nm. Cells are visible in bright field.

NMR Spectra

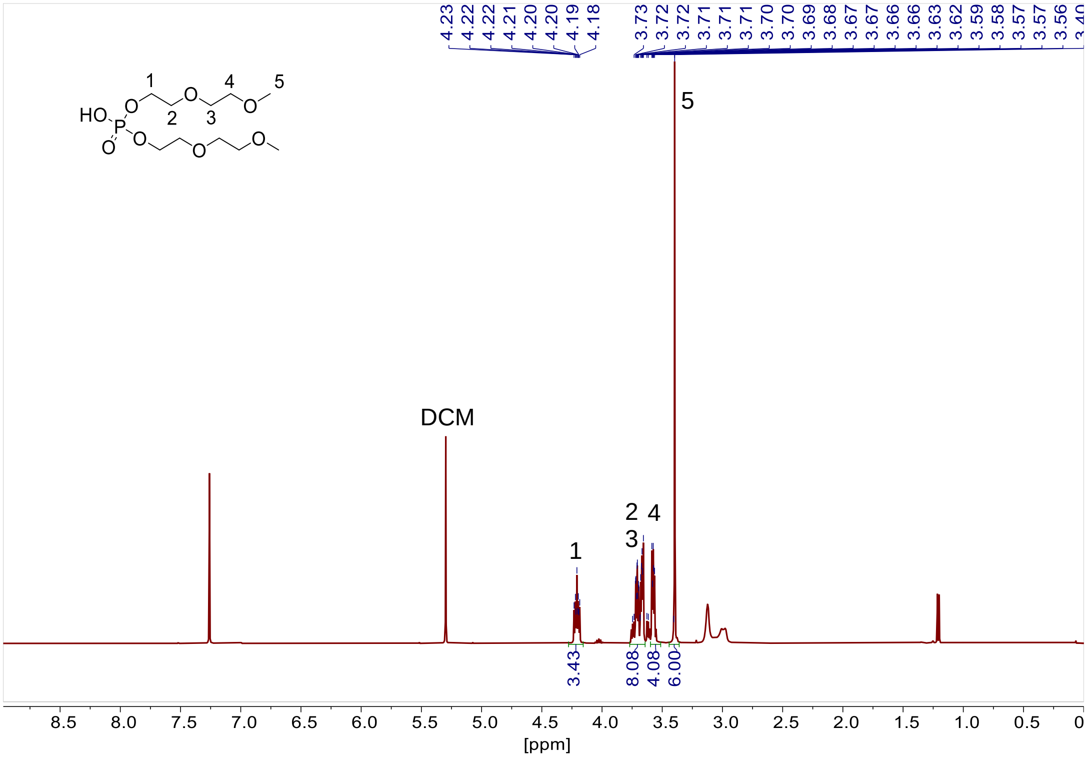

Figure S16: ^{1}H-NMR (400 MHz, $CDCl_3$) of **OEG$_2$-Phosphate** (bis(2-(2-methoxyethoxy)ethyl) hydrogen phosphate).

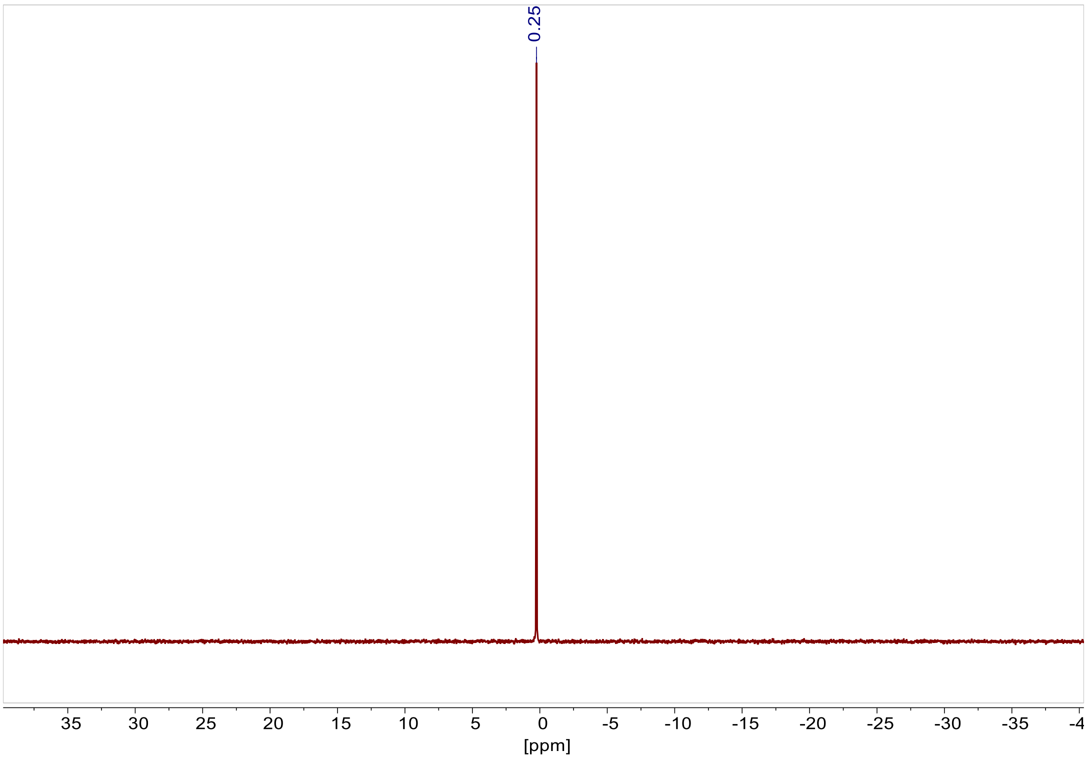

Figure S17: ^{31}P-NMR (162 MHz, $CDCl_3$) of **OEG$_2$-Phosphate** (bis(2-(2-methoxyethoxy)ethyl) hydrogen phosphate).

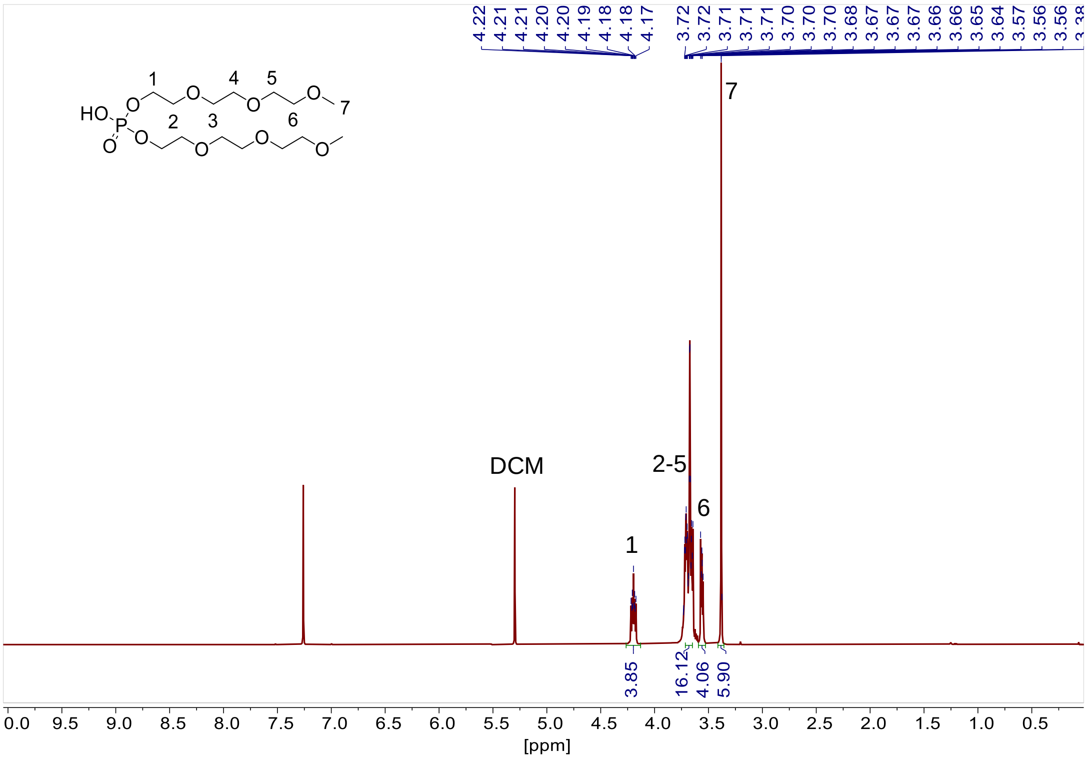

Figure S18: ^{1}H-NMR (400 MHz, $CDCl_3$) of **OEG_3-Phosphate** (bis(2-(2-(2-methoxyethoxy)ethoxy)ethyl) hydrogen phosphate).

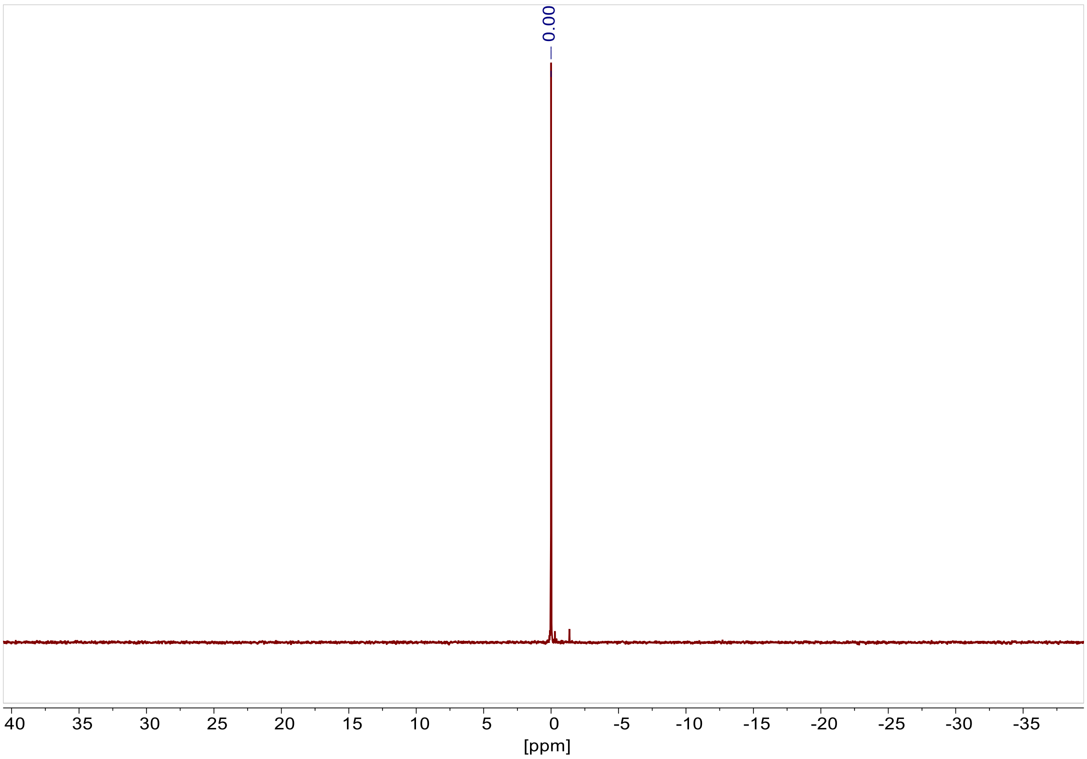

Figure S19: ^{31}P-NMR (162 MHz, $CDCl_3$) of **OEG_3-Phosphate** (bis(2-(2-(2-methoxyethoxy)ethoxy)ethyl) hydrogen phosphate).

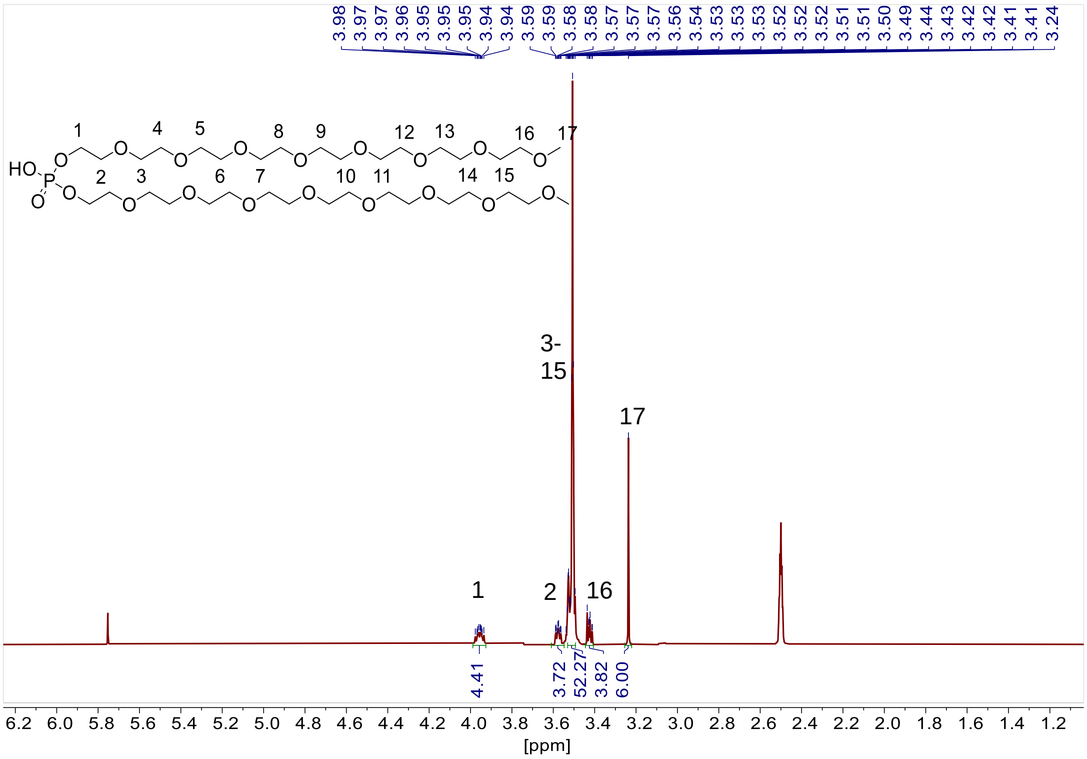

Figure S20: ^{1}H-NMR (400 MHz, DMSO-d$_6$) of **OEG$_8$-Phosphate** (di(2,5,8,11,14,17,20,23-octaoxapentacosan-25-yl) hydrogen phosphate).

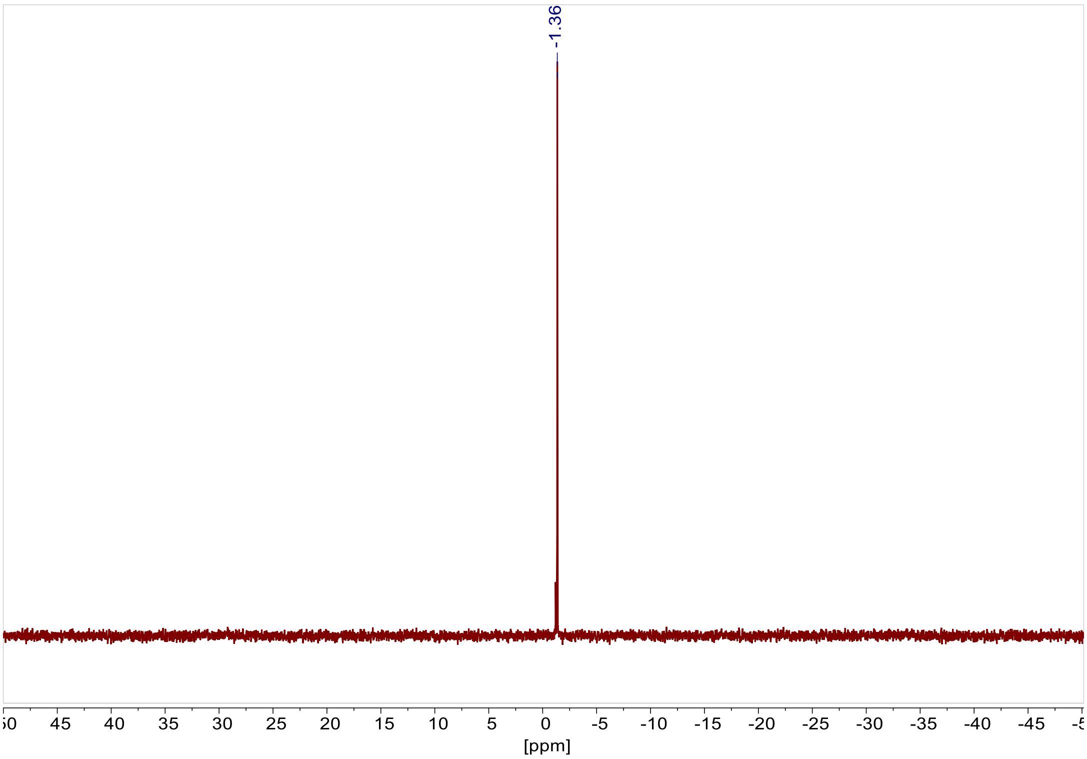

Figure S21: ^{31}P-NMR (243 MHz, DMSO-d$_6$) of **OEG$_8$-Phosphate** (di(2,5,8,11,14,17,20,23-octaoxapentacosan-25-yl) hydrogen phosphate).

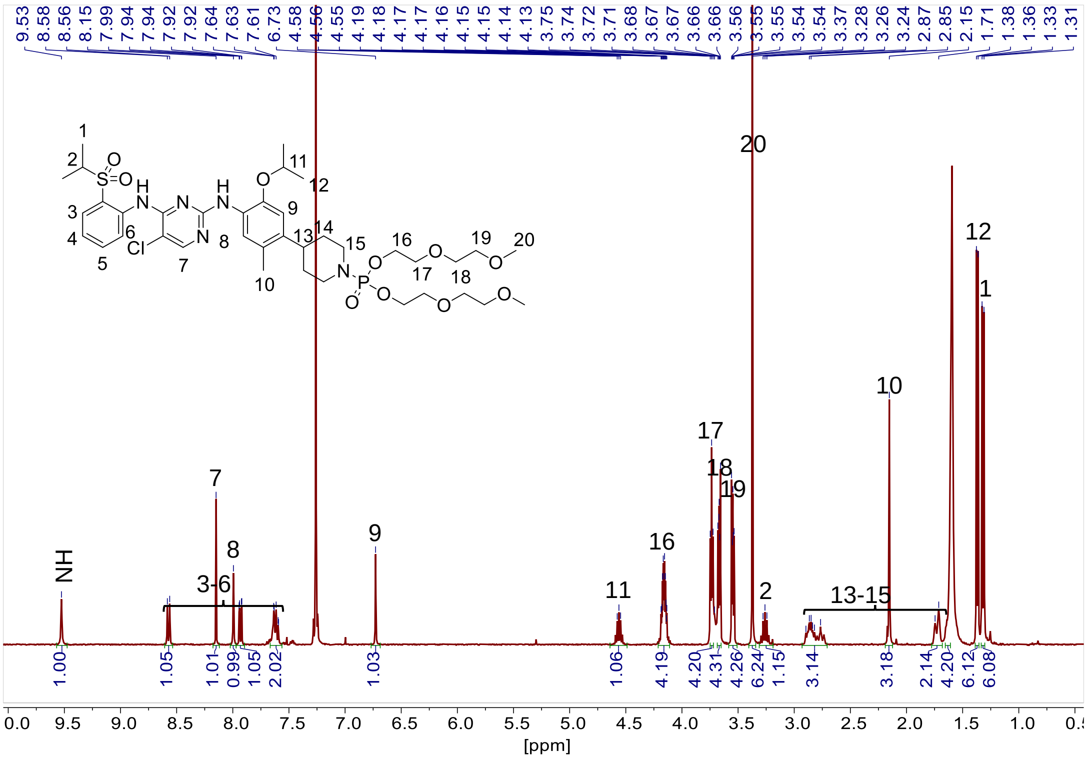

Figure S22: ^{1}H-NMR (400 MHz, $CDCl_3$) of **Cer-OEG$_2$** (bis(2-(2-methoxyethoxy)ethyl) (4-(4-((5-chloro-4-((2-(isopropylsulfonyl)phenyl)amino)pyrimidin-2-yl)amino)-5-isopropoxy-2-methylphenyl)piperidin-1-yl)phosphonate).

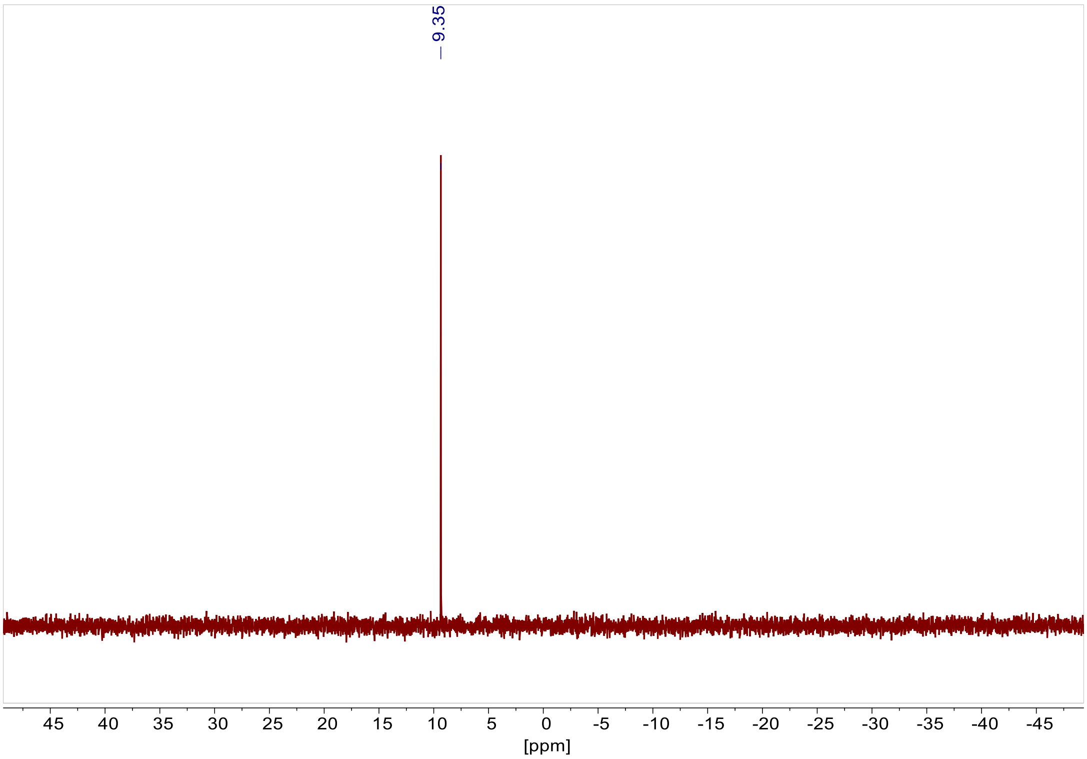

Figure S23: ^{31}P-NMR (162 MHz, $CDCl_3$) of **Cer-OEG$_2$** (bis(2-(2-methoxyethoxy)ethyl) (4-(4-((5-chloro-4-((2-(isopropylsulfonyl)phenyl)amino)pyrimidin-2-yl)amino)-5-isopropoxy-2-methylphenyl)piperidin-1-yl)phosphonate).

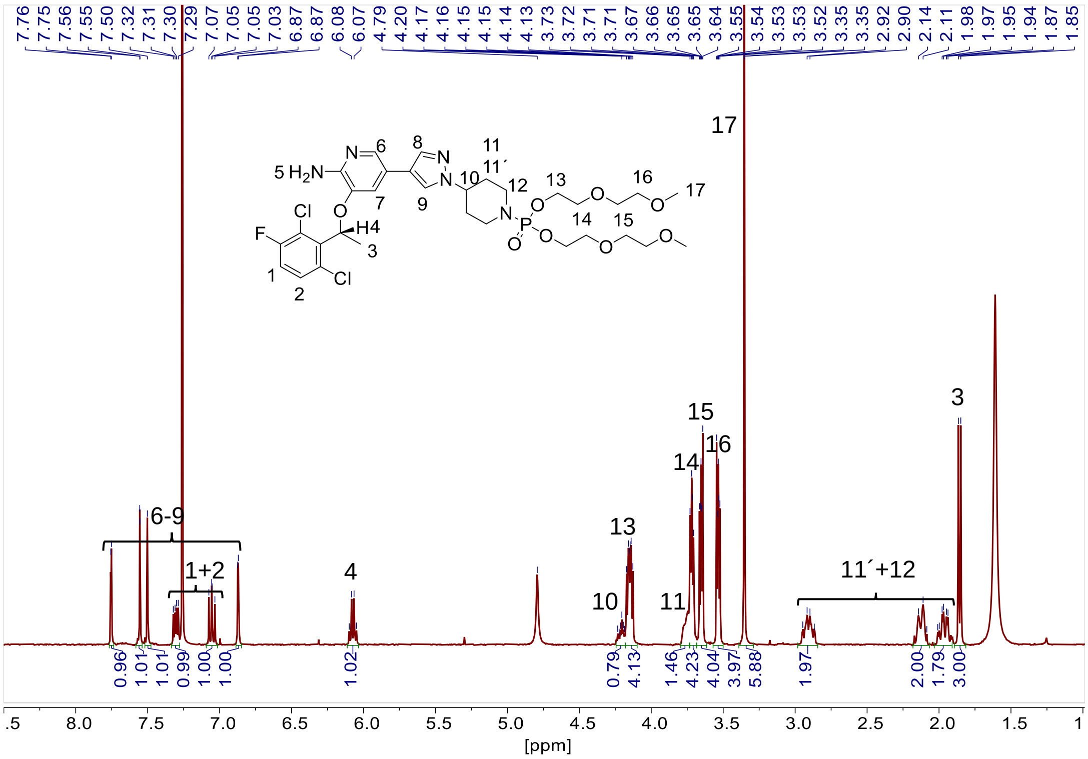

Figure S24: ^{1}H-NMR (400 MHz, $CDCl_3$) of **Cri-OEG$_2$** (bis(2-(2-methoxyethoxy)ethyl) (R)-(4-(4-(6-amino-5-(1-(2,6-dichloro-3-fluorophenyl)ethoxy)pyridin-3-yl)-1H-pyrazol-1-yl)piperidin-1-yl)phosphonate).

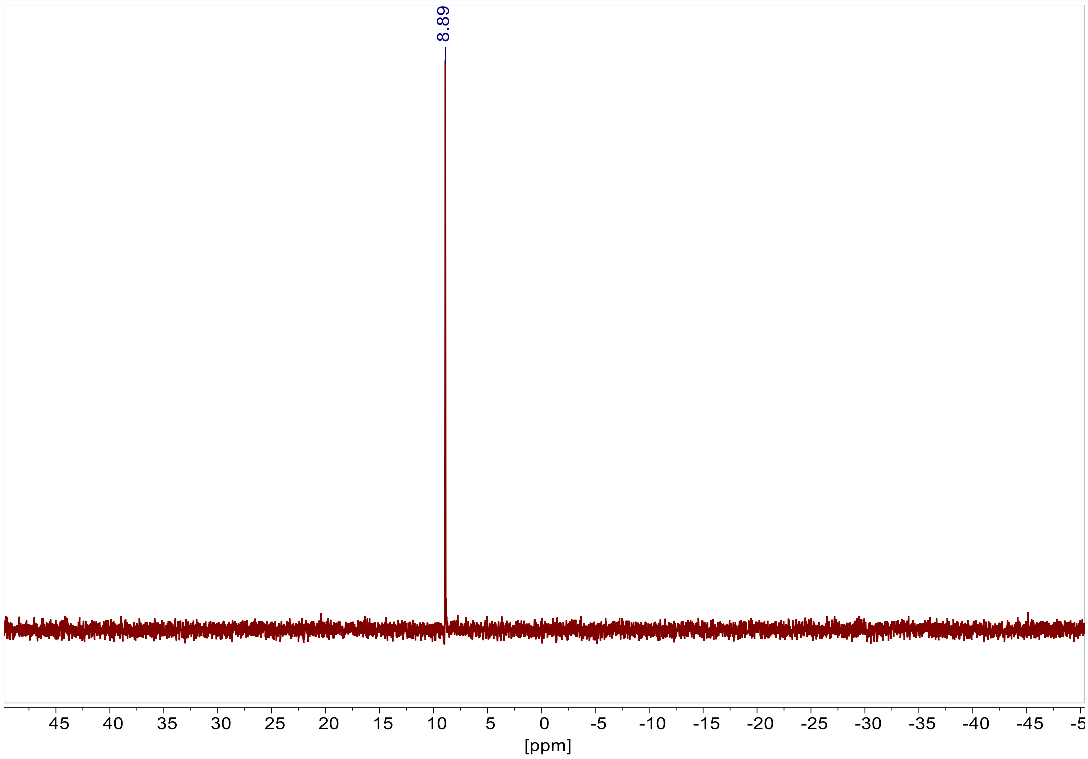

Figure S25: ^{31}P-NMR (162 MHz, $CDCl_3$) of **Cri-OEG$_2$** (bis(2-(2-methoxyethoxy)ethyl) (R)-(4-(4-(6-amino-5-(1-(2,6-dichloro-3-fluorophenyl)ethoxy)pyridin-3-yl)-1H-pyrazol-1-yl)piperidin-1-yl)phosphonate).

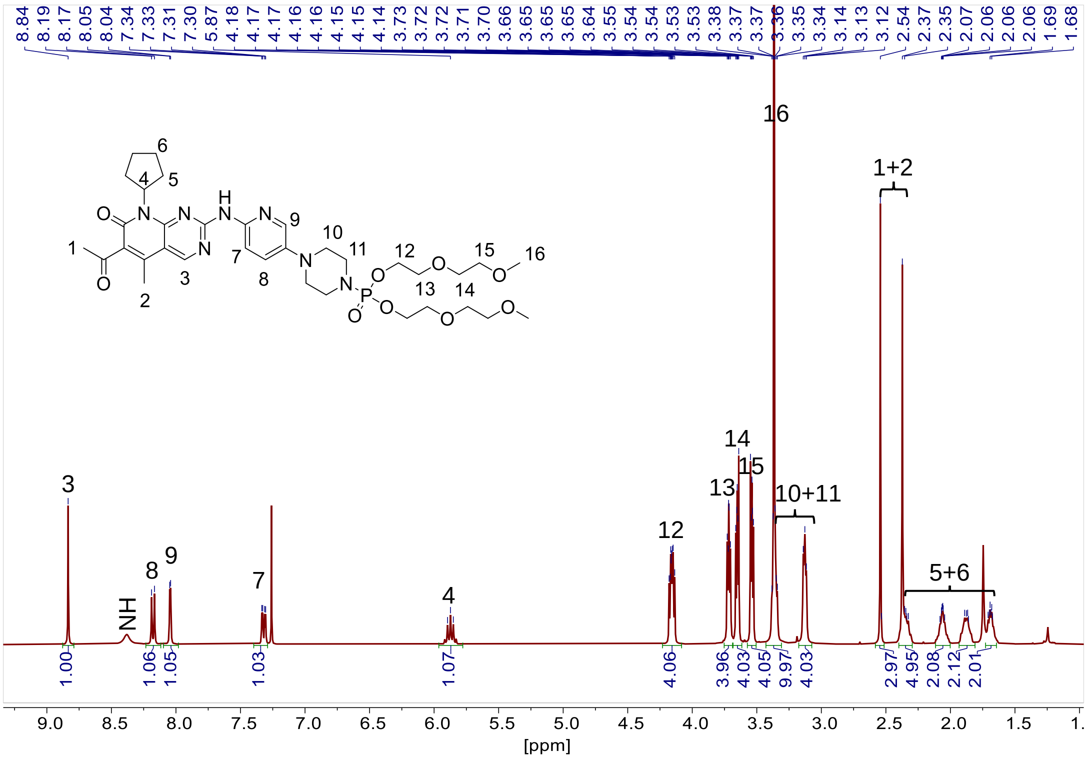

Figure S26: ^{1}H-NMR (400 MHz, $CDCl_3$) of **Pal-OEG$_2$** (bis(2-(2-methoxyethoxy)ethyl) (4-(6-((6-acetyl-8-cyclopentyl-5-methyl-7-oxo-7,8-dihydropyrido[2,3-d]pyrimidin-2-yl)amino)pyridin-3-yl)piperazin-1-yl)phosphonate).

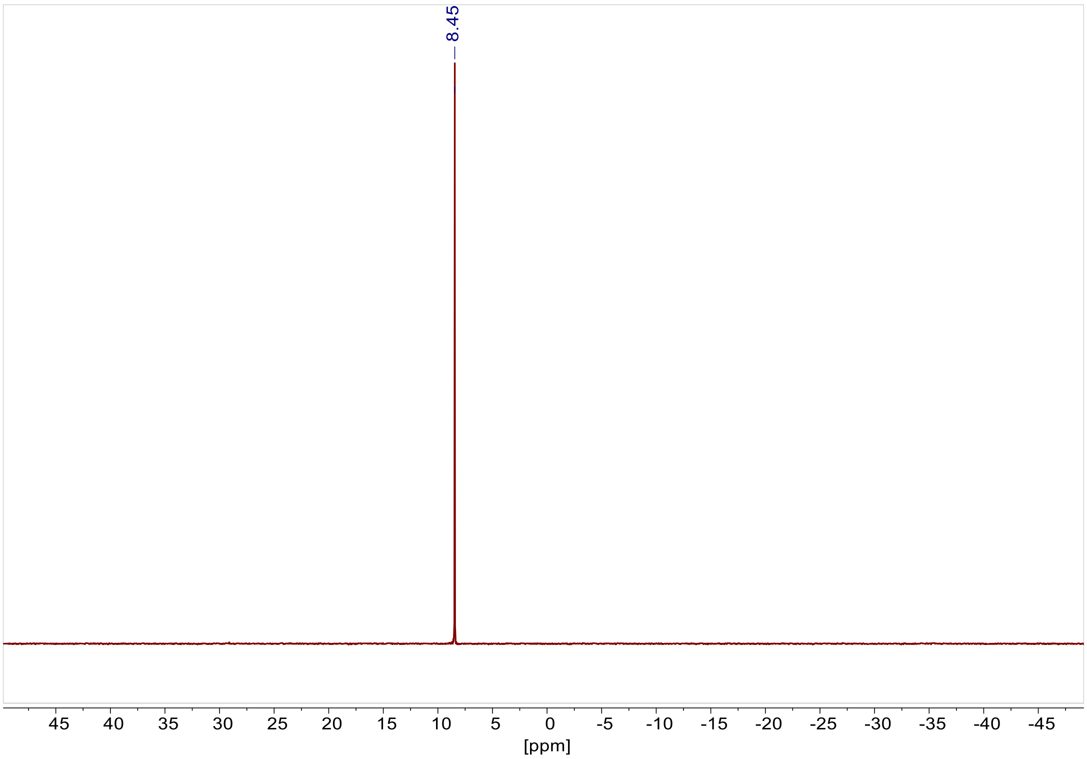

Figure S27: ^{31}P-NMR (162 MHz, $CDCl_3$) of **Pal-OEG$_2$** (bis(2-(2-methoxyethoxy)ethyl) (4-(6-((6-acetyl-8-cyclopentyl-5-methyl-7-oxo-7,8-dihydropyrido[2,3-d]pyrimidin-2-yl)amino)pyridin-3-yl)piperazin-1-yl)phosphonate).

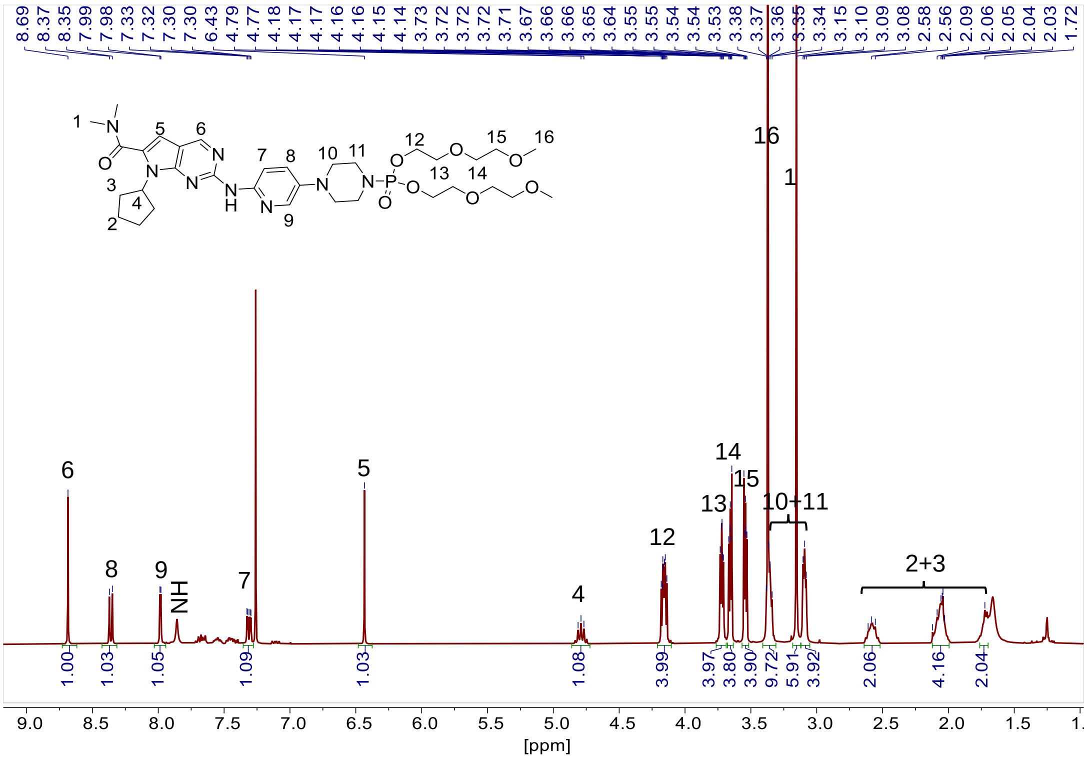

Figure S28: ^{1}H-NMR (400 MHz, $CDCl_3$) of **Rib-OEG$_2$** (bis(2-(2-methoxyethoxy)ethyl) (4-(6-((7-cyclopentyl-6-(dimethylcarbamoyl)-7H-pyrrolo[2,3-d]pyrimidin-2-yl)amino)pyridin-3-yl)piperazin-1-yl)phosphonate).

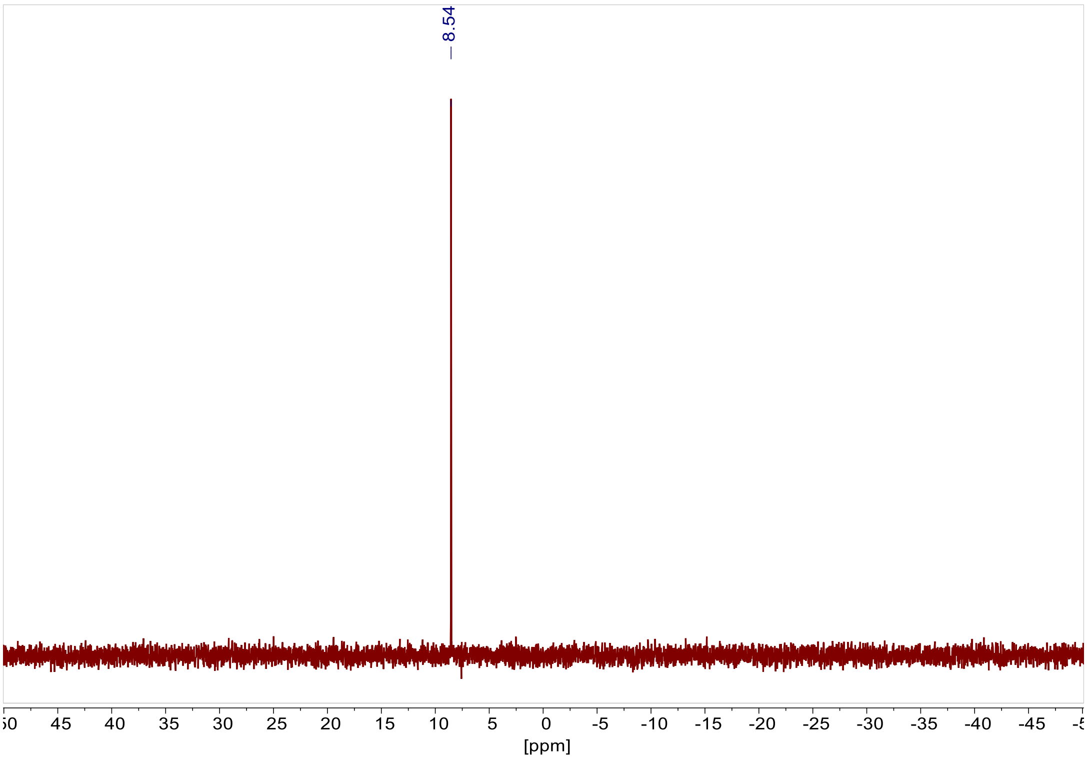

Figure S29: ^{31}P-NMR (162 MHz, $CDCl_3$) of **Rib-OEG$_2$** (bis(2-(2-methoxyethoxy)ethyl) (4-(6-((7-cyclopentyl-6-(dimethylcarbamoyl)-7H-pyrrolo[2,3-d]pyrimidin-2-yl)amino)pyridin-3-yl)piperazin-1-yl)phosphonate).

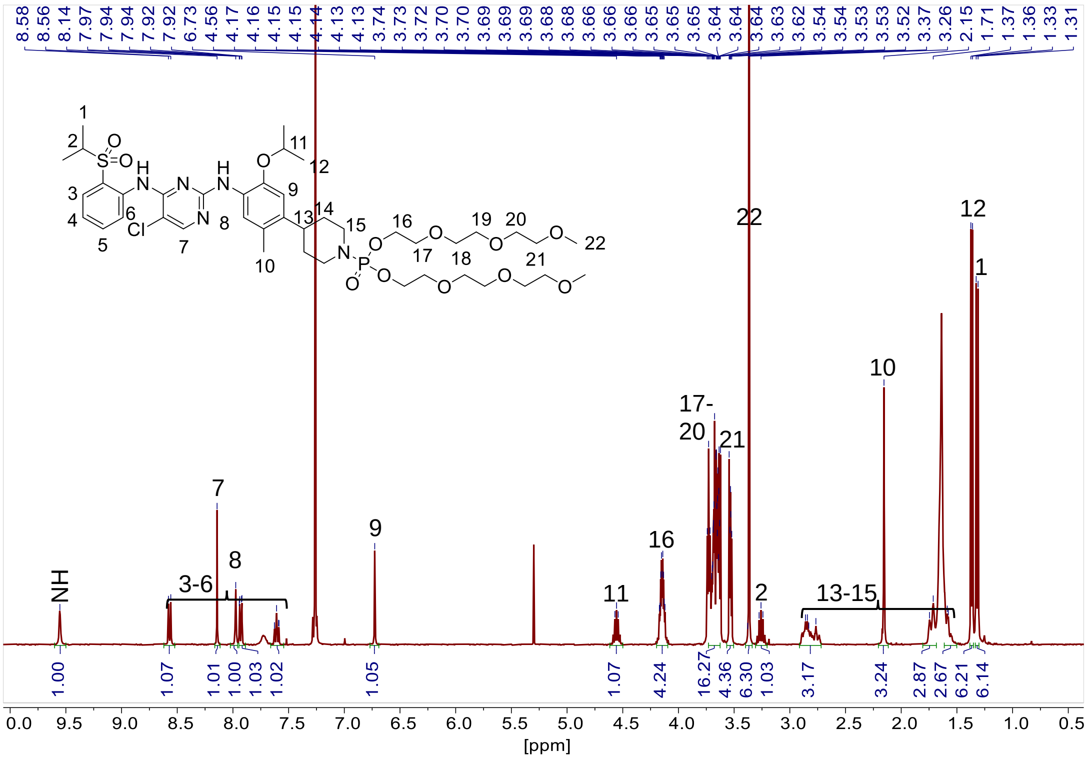

Figure S30: ^{1}H-NMR (400 MHz, $CDCl_3$) of **Cer-OEG$_3$** (bis(2-(2-(2-methoxyethoxy)ethoxy)ethyl) (4-(4-((5-chloro-4-((2-(isopropylsulfonyl)phenyl)amino)pyrimidin-2-yl)amino)-5-isopropoxy-2-methylphenyl)piperidin-1-yl)phosphonate).

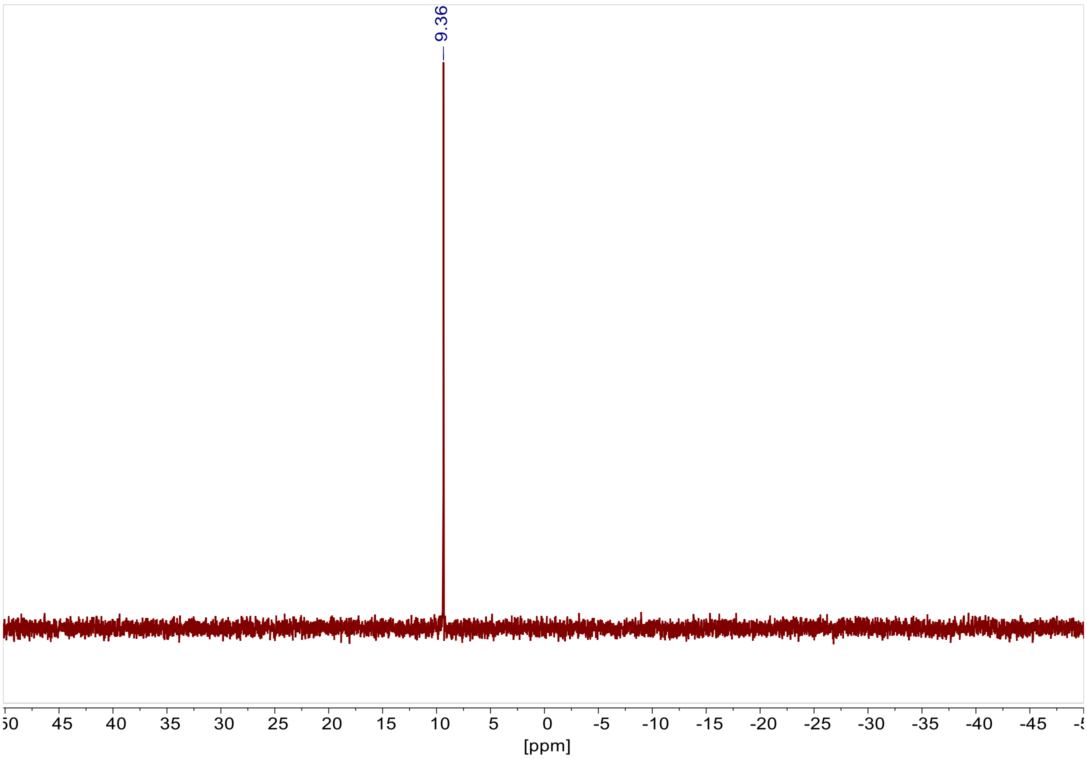

Figure S31: ^{31}P-NMR (162 MHz, $CDCl_3$) of **Cer-OEG$_3$** (bis(2-(2-(2-methoxyethoxy)ethoxy)ethyl) (4-(4-((5-chloro-4-((2-(isopropylsulfonyl)phenyl)amino)pyrimidin-2-yl)amino)-5-isopropoxy-2-methylphenyl)piperidin-1-yl)phosphonate).

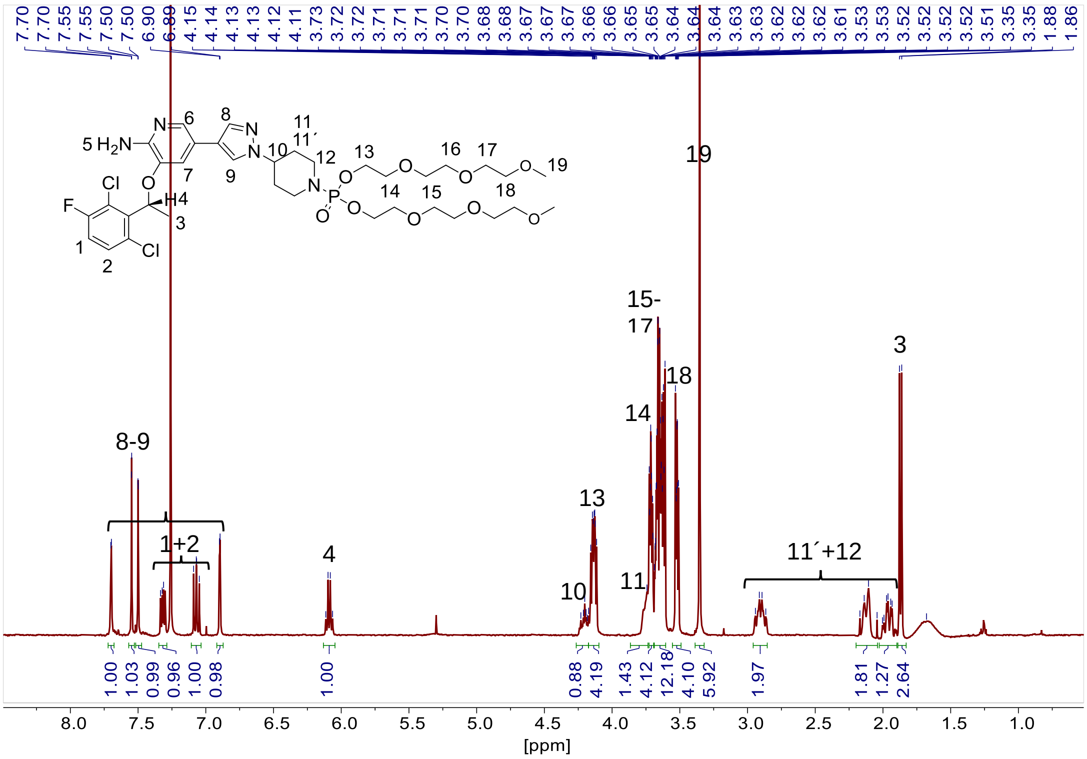

Figure S32: ^{1}H-NMR (400 MHz, $CDCl_3$) of **Cri-OEG$_3$** (bis(2-(2-(2-methoxyethoxy)ethoxy)ethyl) (R)-(4-(4-(6-amino-5-(1-(2,6-dichloro-3-fluorophenyl)ethoxy)pyridin-3-yl)-1H-pyrazol-1-yl)piperidin-1-yl)phosphonate).

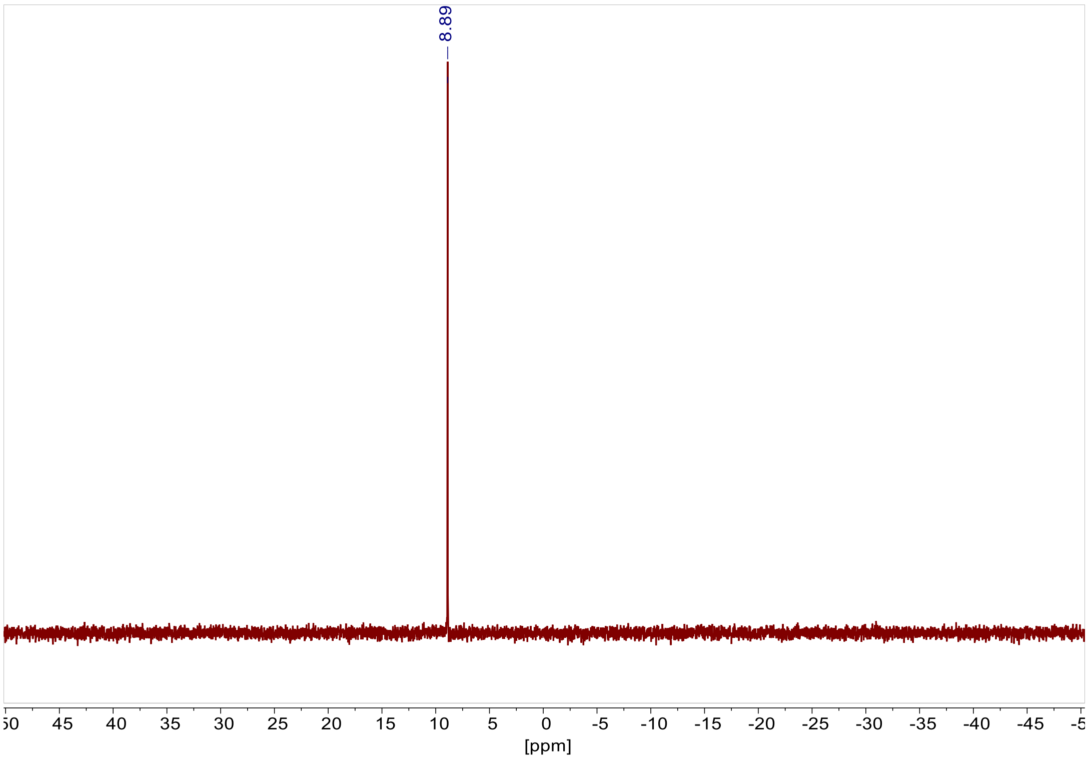

Figure S33: ^{31}P-NMR (162 MHz, $CDCl_3$) of **Cri-OEG$_3$** (bis(2-(2-(2-methoxyethoxy)ethoxy)ethyl) (R)-(4-(4-(6-amino-5-(1-(2,6-dichloro-3-fluorophenyl)ethoxy)pyridin-3-yl)-1H-pyrazol-1-yl)piperidin-1-yl)phosphonate).

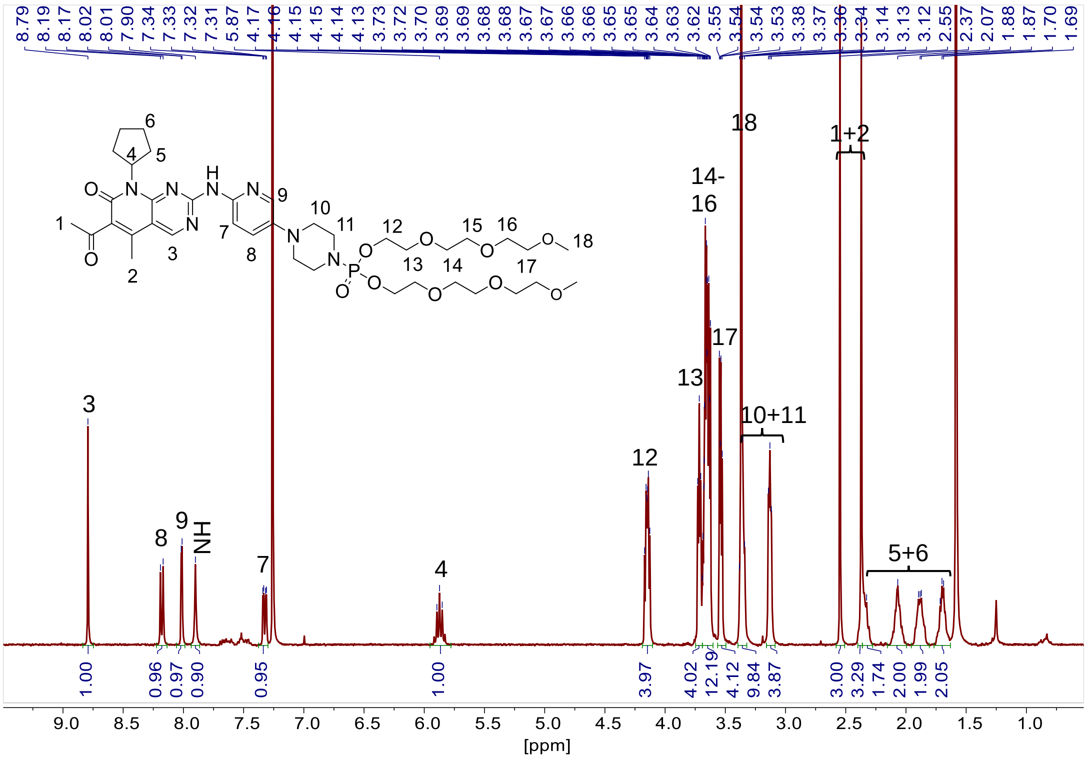

Figure S34: ^{1}H-NMR (400 MHz, $CDCl_3$) of **Pal-OEG$_3$** (bis(2-(2-(2-methoxyethoxy)ethoxy)ethyl) (4-(6-((6-acetyl-8-cyclopentyl-5-methyl-7-oxo-7,8-dihydropyrido[2,3-d]pyrimidin-2-yl)amino)pyridin-3-yl)piperazin-1-yl)phosphonate).

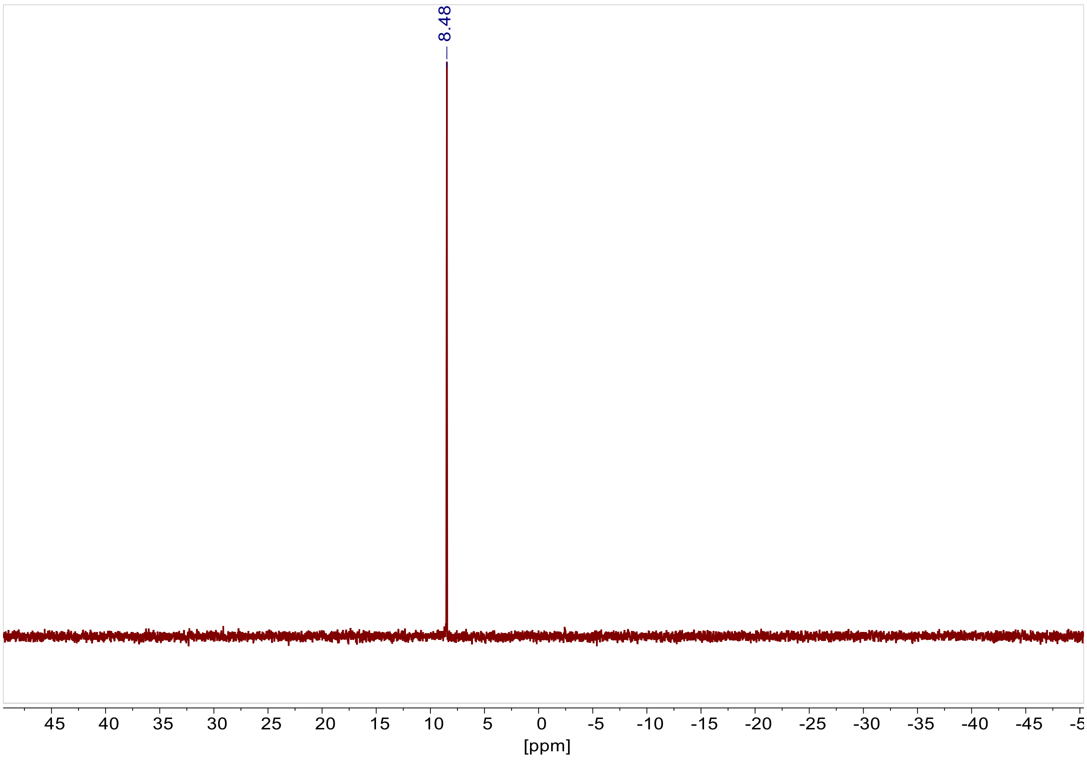

Figure S35: ^{31}P-NMR (162 MHz, $CDCl_3$) of **Pal-OEG$_3$** (bis(2-(2-(2-methoxyethoxy)ethoxy)ethyl) (4-(6-((6-acetyl-8-cyclopentyl-5-methyl-7-oxo-7,8-dihydropyrido[2,3-d]pyrimidin-2-yl)amino)pyridin-3-yl)piperazin-1-yl)phosphonate).

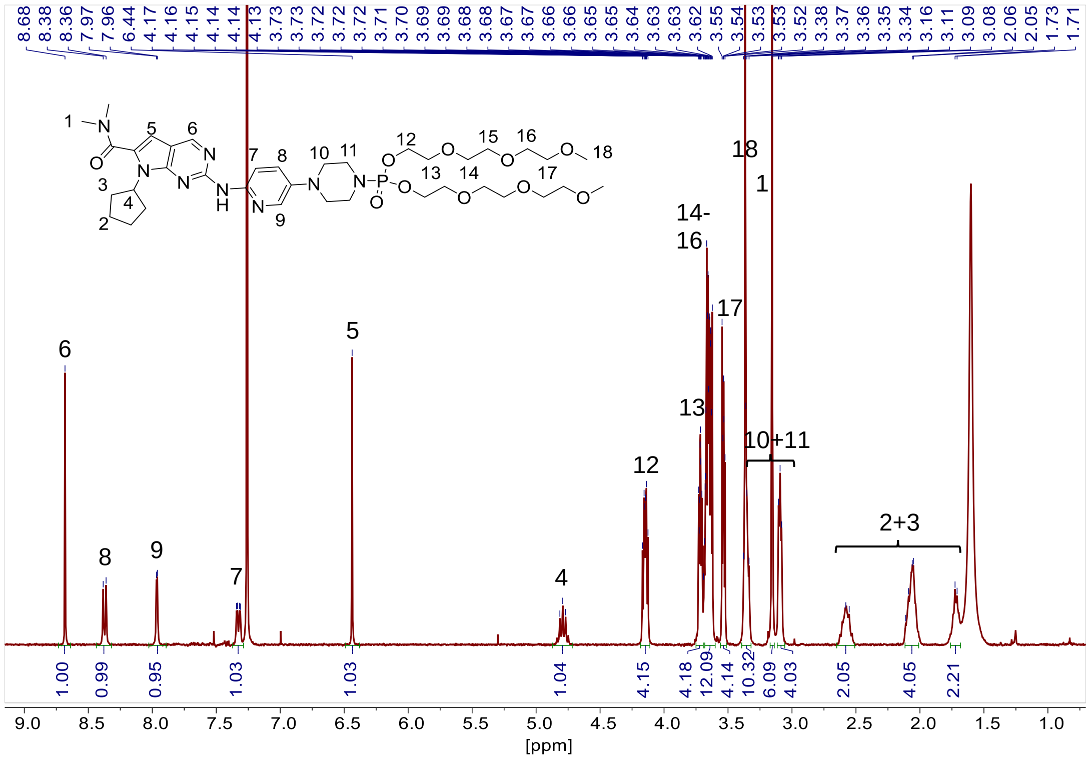

Figure S36: ^{1}H-NMR (400 MHz, $CDCl_3$) of **Rib-OEG$_3$** (bis(2-(2-(2-methoxyethoxy)ethoxy)ethyl) (4-(6-((7-cyclopentyl-6-(dimethylcarbamoyl)-7H-pyrrolo[2,3-d]pyrimidin-2-yl)amino)pyridin-3-yl)piperazin-1-yl)phosphonate).

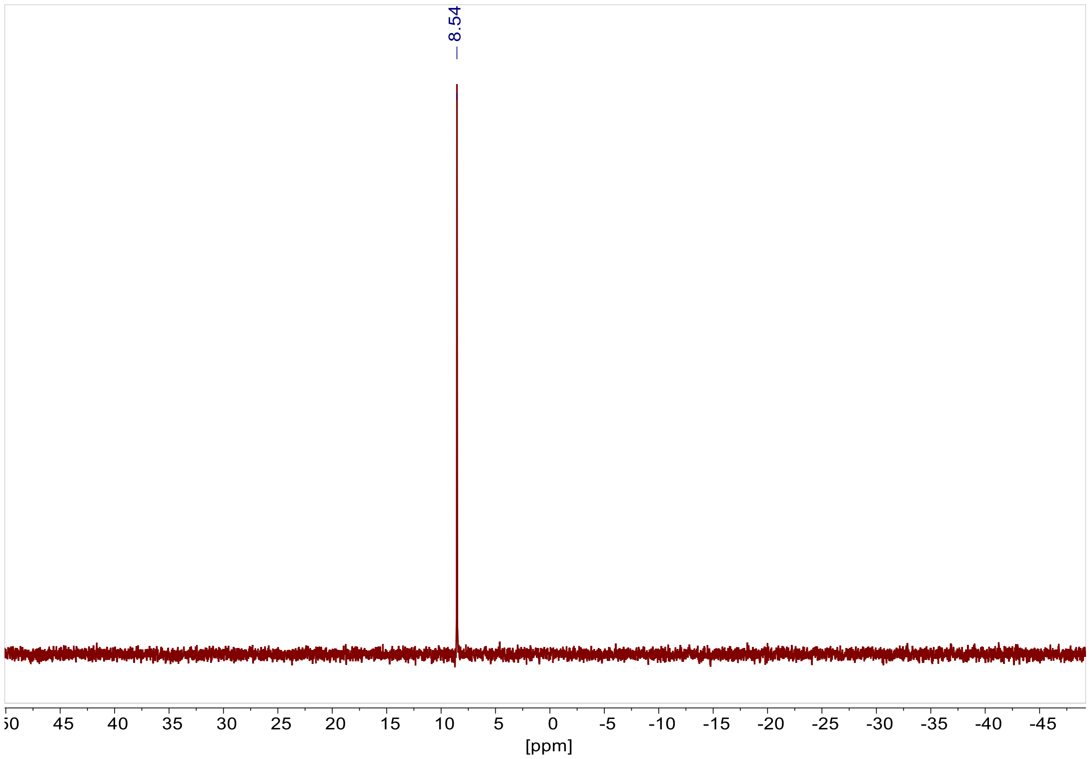

Figure S37: ^{31}P-NMR (162 MHz, $CDCl_3$) of **Rib-OEG$_3$** (bis(2-(2-(2-methoxyethoxy)ethoxy)ethyl) (4-(6-((7-cyclopentyl-6-(dimethylcarbamoyl)-7H-pyrrolo[2,3-d]pyrimidin-2-yl)amino)pyridin-3-yl)piperazin-1-yl)phosphonate).

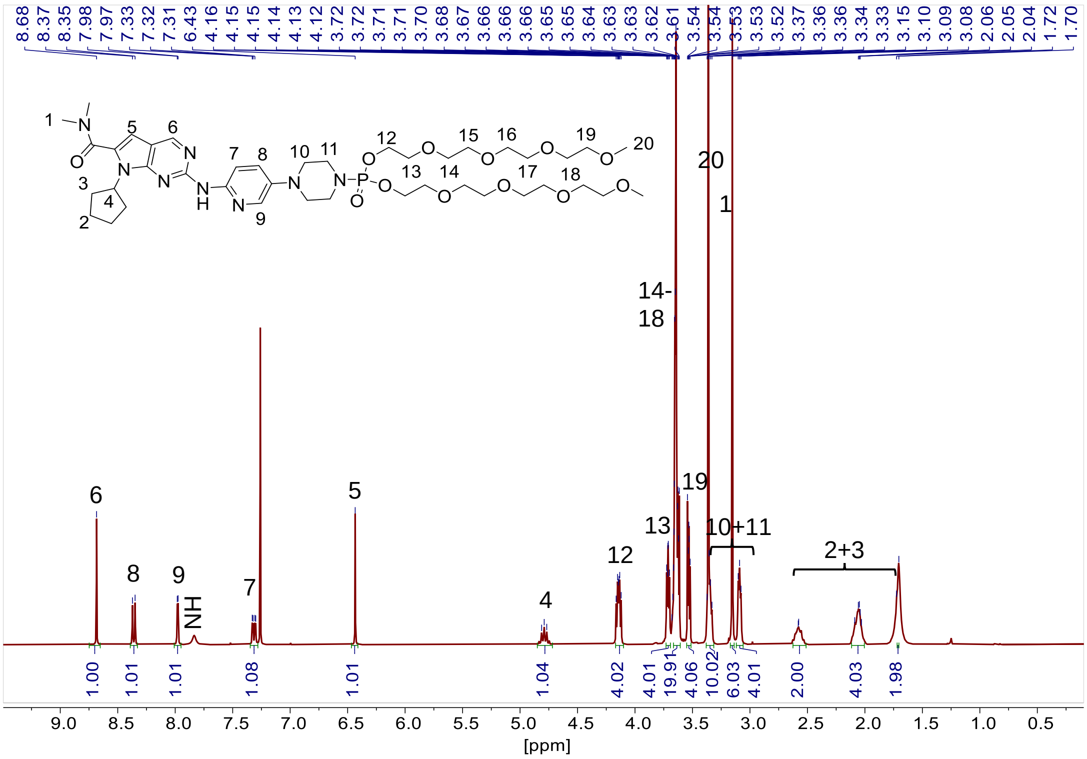

Figure S38: ^{1}H-NMR (400 MHz, $CDCl_3$) of **Rib-OEG$_4$** (di(2,5,8,11-tetraoxatridecan-13-yl) (4-(6-((7-cyclopentyl-6-(dimethylcarbamoyl)-7H-pyrrolo[2,3-d]pyrimidin-2-yl)amino)pyridin-3-yl)piperazin-1-yl)phosphonate).

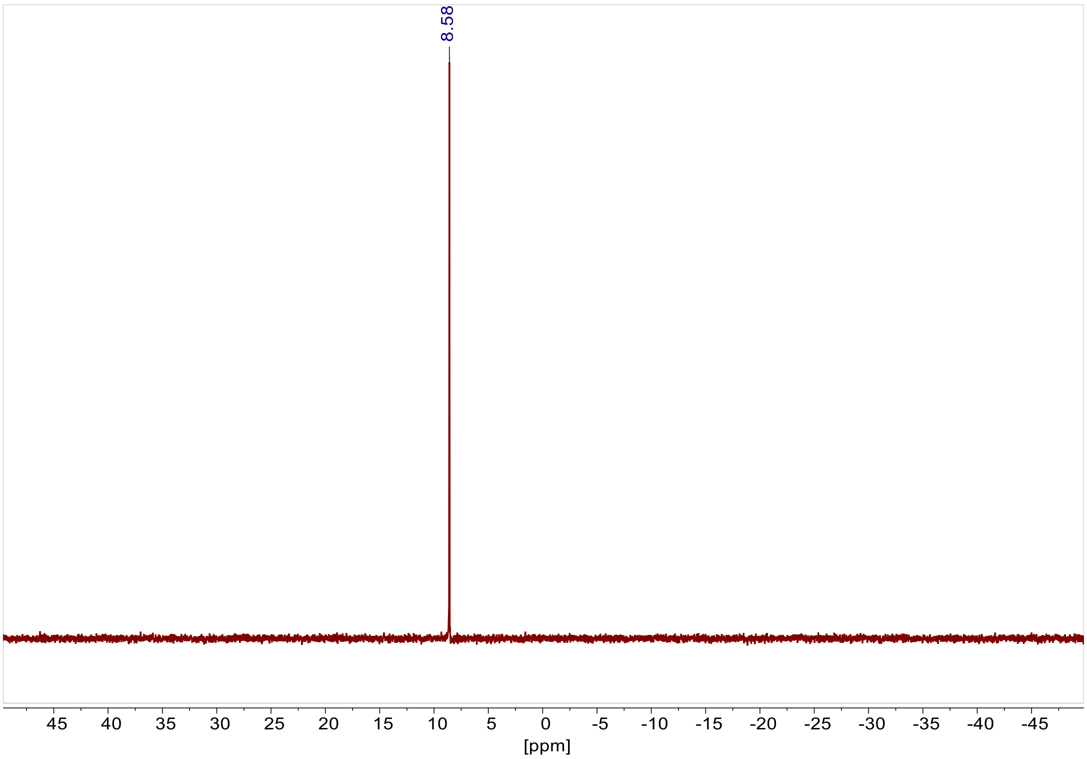

Figure S39: ^{31}P-NMR (162 MHz, $CDCl_3$) of **Rib-OEG$_4$** (di(2,5,8,11-tetraoxatridecan-13-yl) (4-(6-((7-cyclopentyl-6-(dimethylcarbamoyl)-7H-pyrrolo[2,3-d]pyrimidin-2-yl)amino)pyridin-3-yl)piperazin-1-yl)phosphonate).

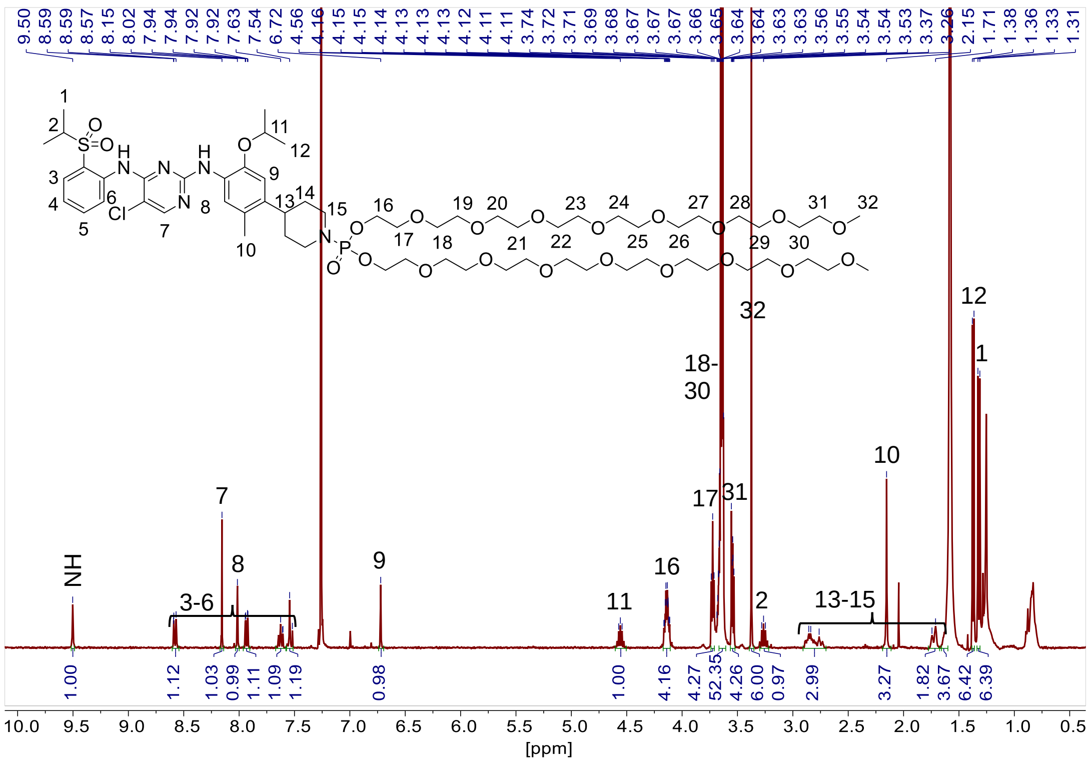

Figure S40: ^{1}H-NMR (400 MHz, $CDCl_3$) of **Cer-OEG$_8$** (di(2,5,8,11,14,17,20,23-octaoxapentacosan-25-yl) (4-(4-((5-chloro-4-((2-(isopropylsulfonyl)phenyl)amino)pyrimidin-2-yl)amino)-5-isopropoxy-2-methylphenyl)piperidin-1-yl)phosphonate).

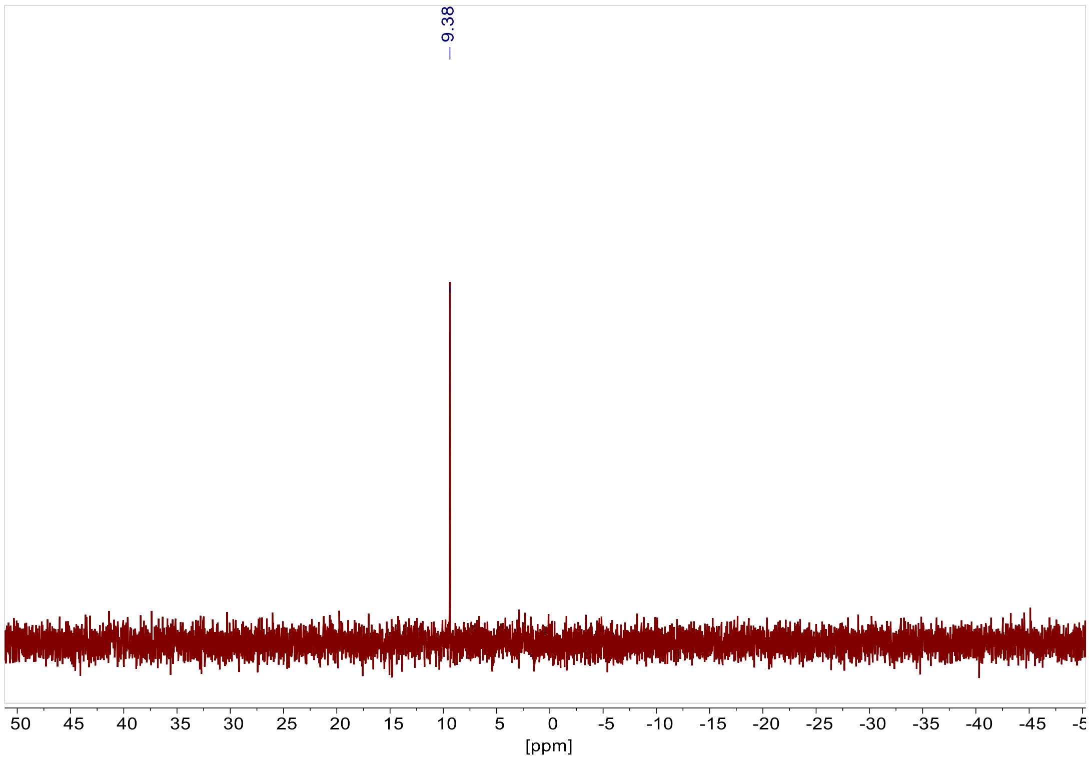

Figure S41: ^{31}P-NMR (243 MHz, $CDCl_3$) of **Cer-OEG$_8$** (di(2,5,8,11,14,17,20,23-octaoxapentacosan-25-yl) (4-(4-((5-chloro-4-((2-(isopropylsulfonyl)phenyl)amino)pyrimidin-2-yl)amino)-5-isopropoxy-2-methylphenyl)piperidin-1-yl)phosphonate).

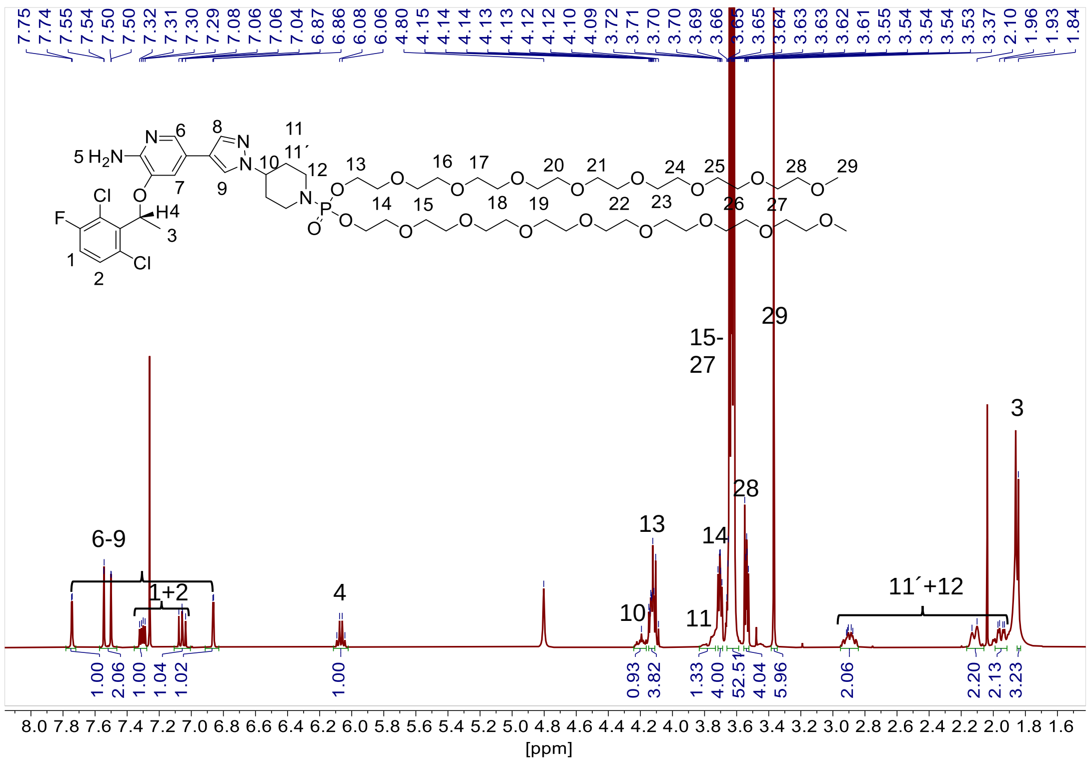

Figure S42: ^{1}H-NMR (400 MHz, $CDCl_3$) of **Cri-OEG$_8$** (di(2,5,8,11,14,17,20,23-octaoxapentacosan-25-yl) (R)-(4-(4-(6-amino-5-(1-(2,6-dichloro-3-fluorophenyl)ethoxy)pyridin-3-yl)-1H-pyrazol-1-yl)piperidin-1-yl)phosphonate).

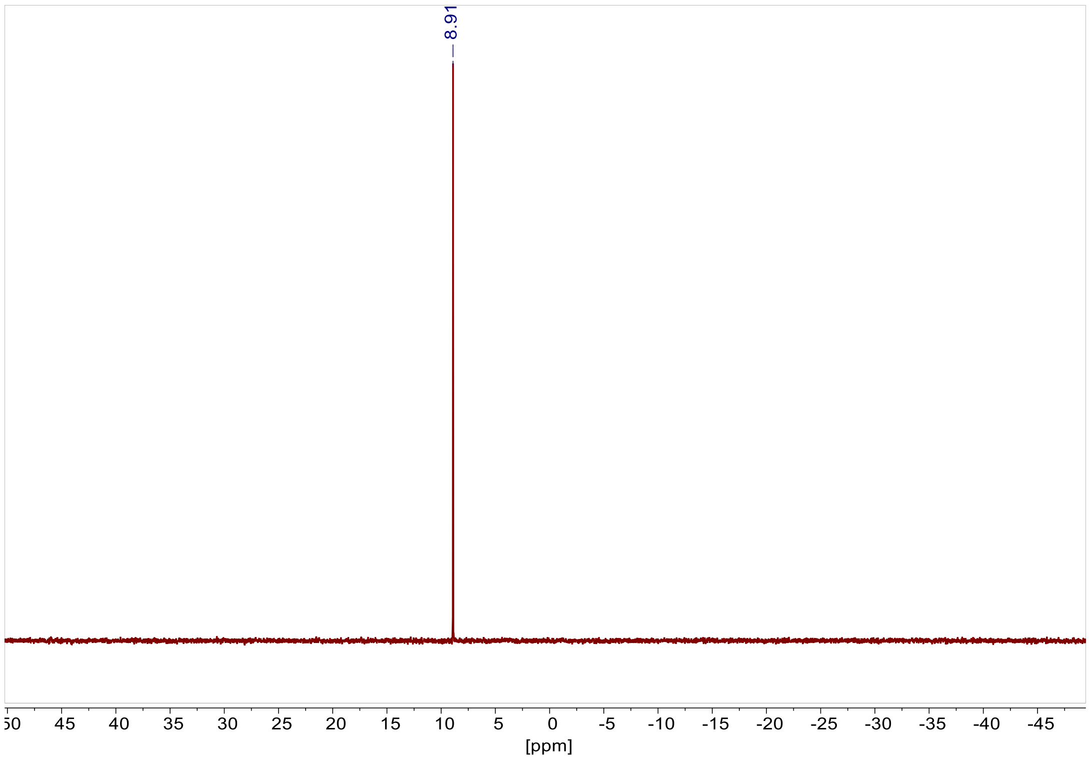

Figure S43: ^{31}P-NMR (243 MHz, $CDCl_3$) of **Cri-OEG$_8$** (di(2,5,8,11,14,17,20,23-octaoxapentacosan-25-yl) (R)-(4-(4-(6-amino-5-(1-(2,6-dichloro-3-fluorophenyl)ethoxy)pyridin-3-yl)-1H-pyrazol-1-yl)piperidin-1-yl)phosphonate).

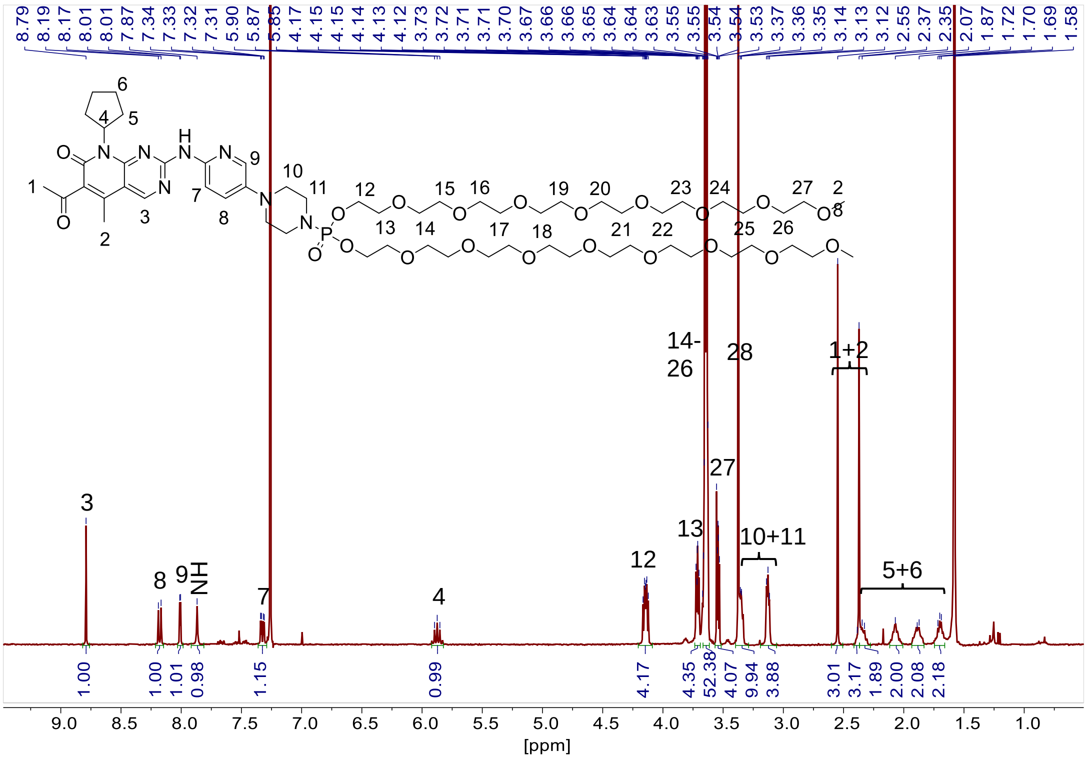

Figure S44: ^{1}H-NMR (400 MHz, $CDCl_3$) of **Pal-OEG$_8$** (di(2,5,8,11,14,17,20,23-octaoxapentacosan-25-yl) (4-(6-((6-acetyl-8-cyclopentyl-5-methyl-7-oxo-7,8-dihydropyrido[2,3-d]pyrimidin-2-yl)amino)pyridin-3-yl)piperazin-1-yl)phosphonate).

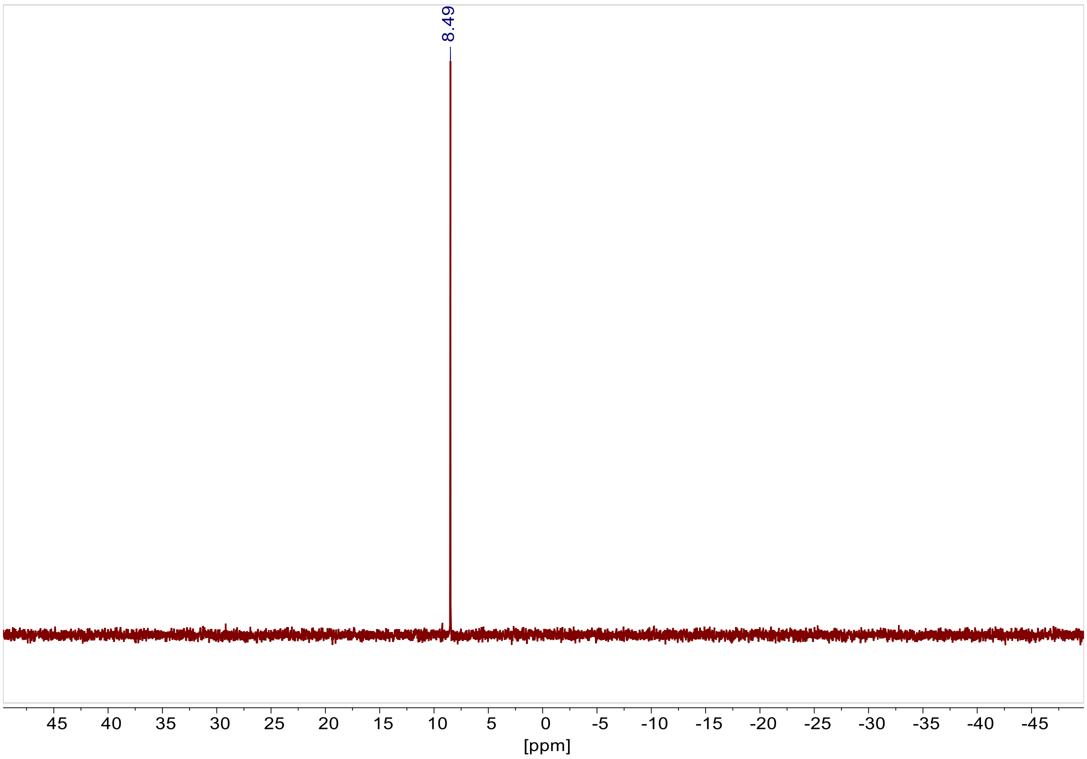

Figure S45: ^{31}P-NMR (243 MHz, $CDCl_3$) of **Pal-OEG$_8$** (di(2,5,8,11,14,17,20,23-octaoxapentacosan-25-yl) (4-(6-((6-acetyl-8-cyclopentyl-5-methyl-7-oxo-7,8-dihydropyrido[2,3-d]pyrimidin-2-yl)amino)pyridin-3-yl)piperazin-1-yl)phosphonate).

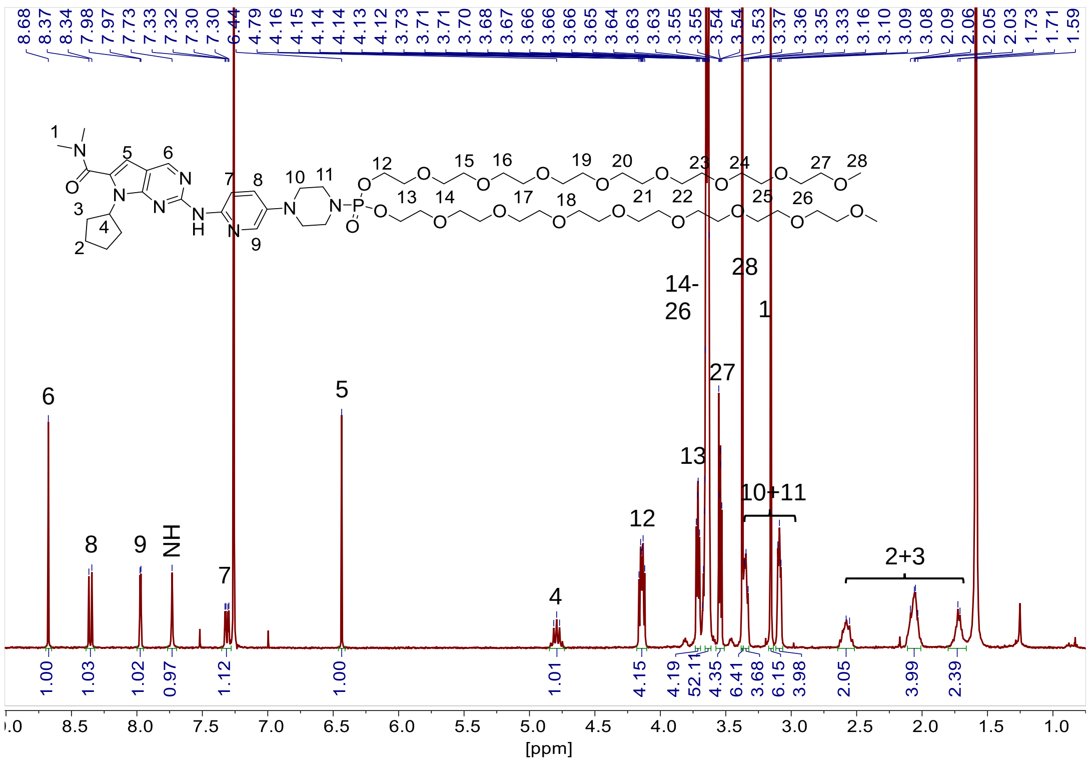

Figure S46: ^{1}H-NMR (400 MHz, $CDCl_3$) of **Rib-OEG$_8$** (di(2,5,8,11,14,17,20,23-octaoxapentacosan-25-yl) (4-(6-((7-cyclopentyl-6-(dimethylcarbamoyl)-7H-pyrrolo[2,3-d]pyrimidin-2-yl)amino)pyridin-3-yl)piperazin-1-yl)phosphonate).

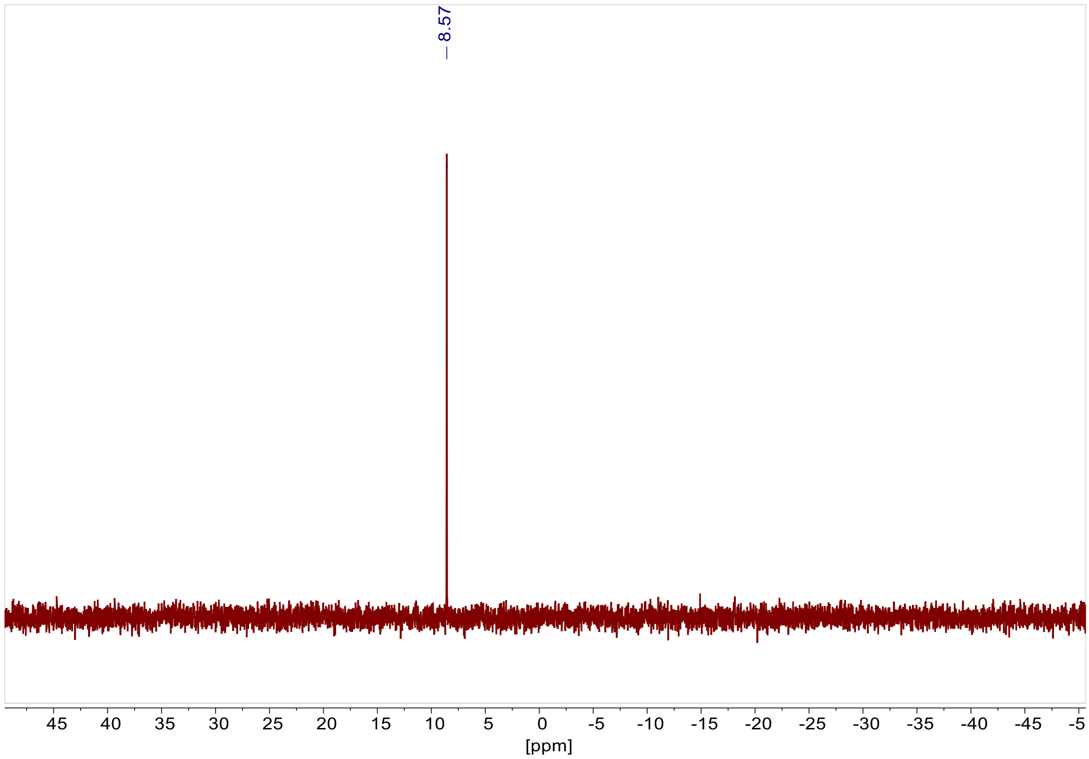

Figure S47: ^{31}P-NMR (243 MHz, $CDCl_3$) of **Rib-OEG$_8$** (di(2,5,8,11,14,17,20,23-octaoxapentacosan-25-yl) (4-(6-((7-cyclopentyl-6-(dimethylcarbamoyl)-7H-pyrrolo[2,3-d]pyrimidin-2-yl)amino)pyridin-3-yl)piperazin-1-yl)phosphonate).

Mass Spectra

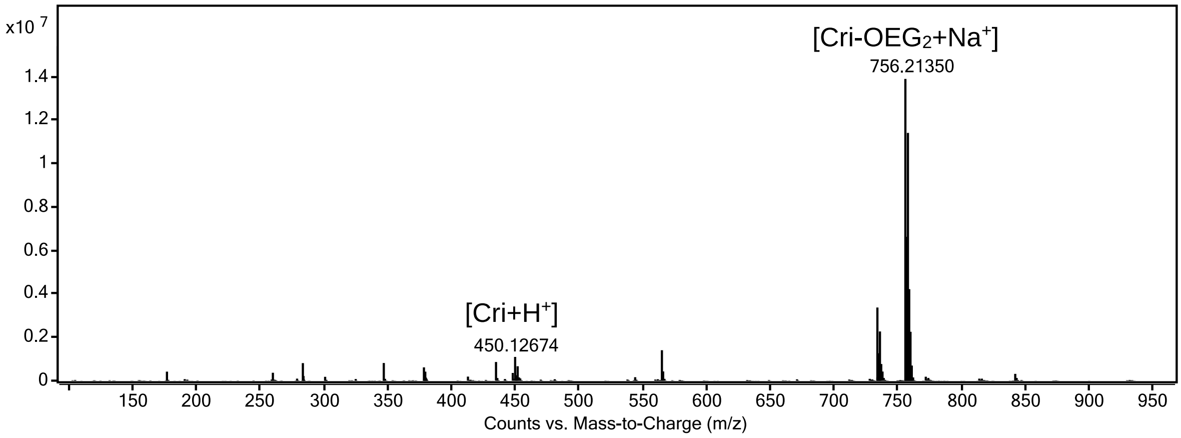

Figure S48: ESI(+) of **Cri-OEG₂** at pH 3 after 48 h.

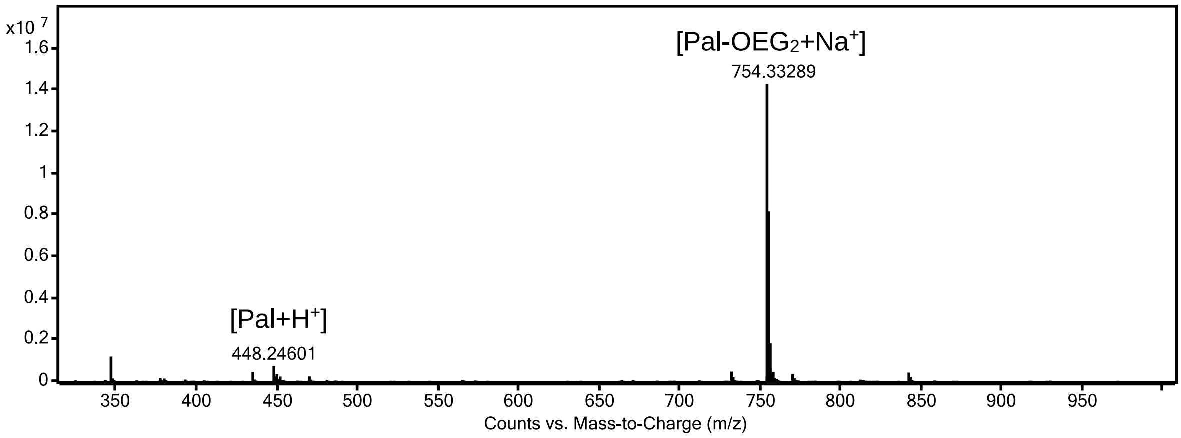

Figure S49: ESI(+) of **Pal-OEG₂** at pH 3 after 48 h.

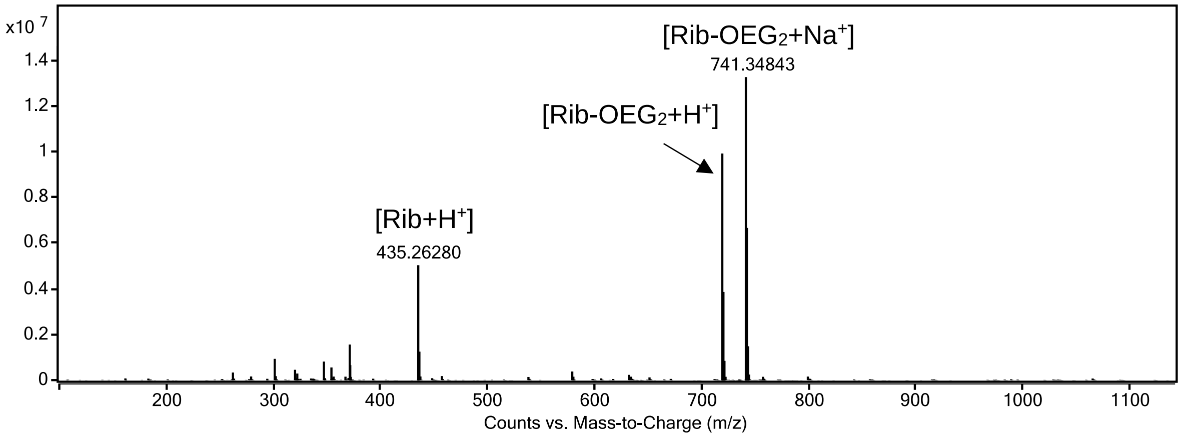

Figure S50: ESI(+) of **Rib-OEG₂** at pH 3 after 48 h.

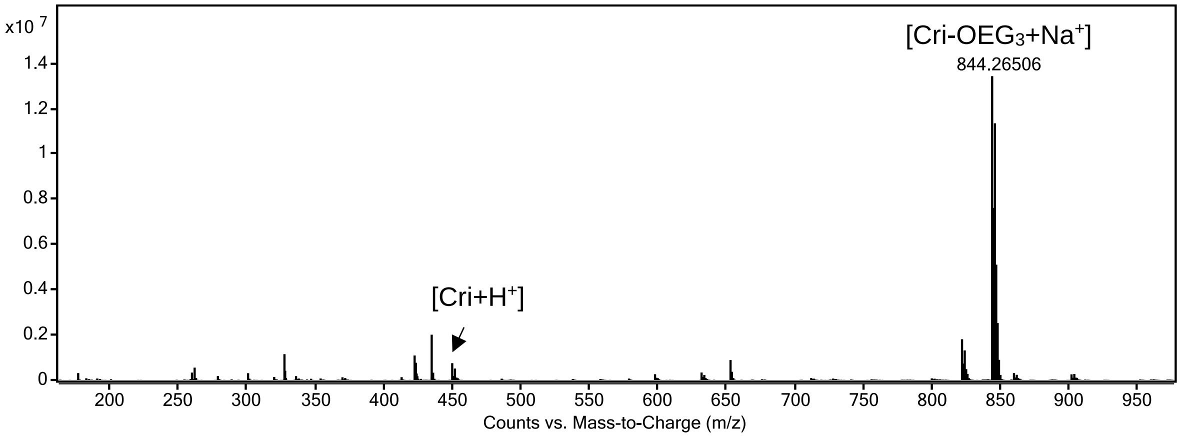

Figure S51: ESI(+) of **Cri-OEG$_3$** at pH 3 after 48 h.

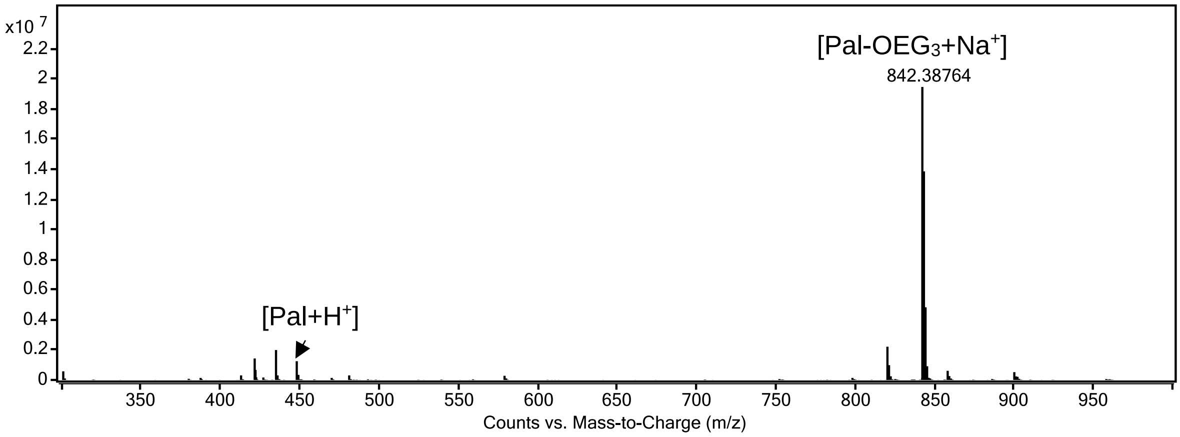

Figure S52: ESI(+) of **Pal-OEG$_3$** at pH 3 after 48 h.

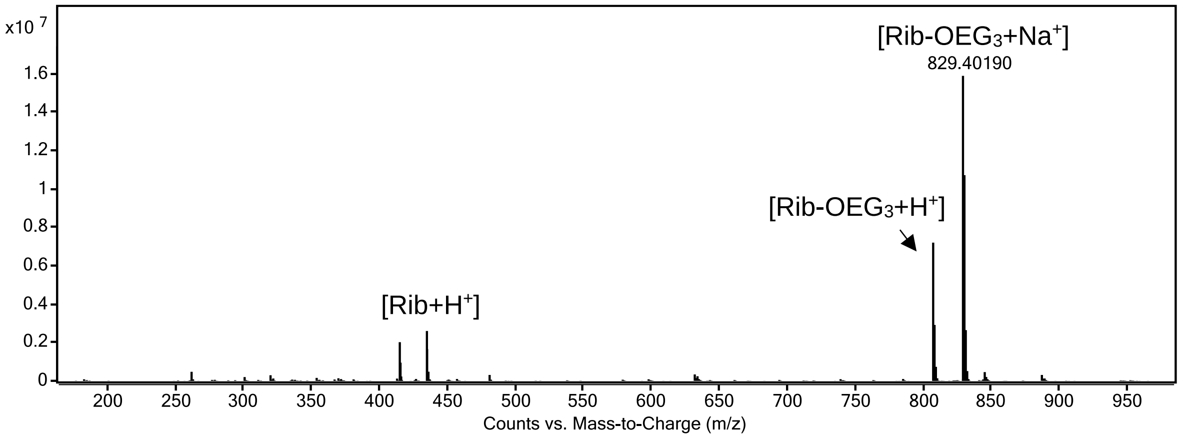

Figure S53: ESI(+) of **Rib-OEG$_3$** at pH 3 after 48 h.

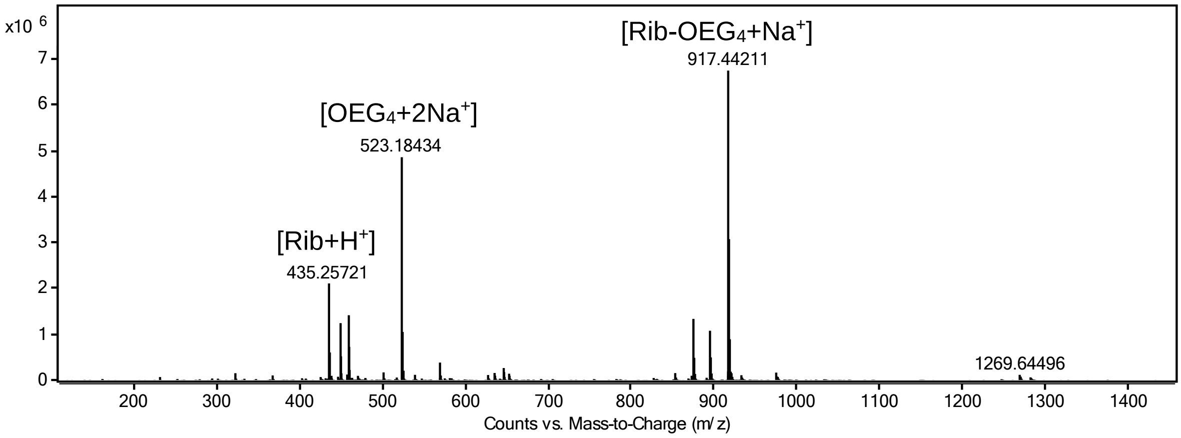

Figure S54: ESI(+) of **Rib-OEG$_4$** at pH 3 after 48 h. OEG$_4$ is the abbreviation for the OEG$_4$-Phosphate.

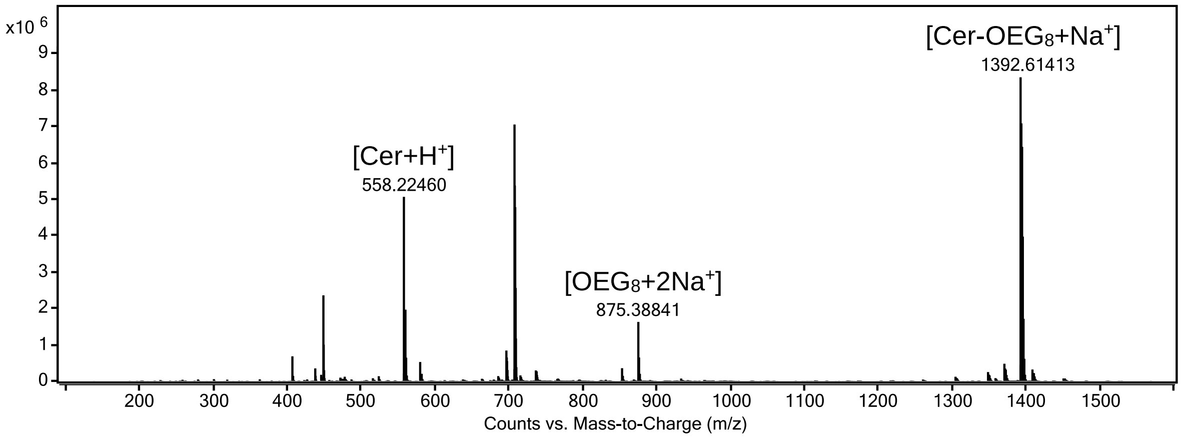

Figure S55: ESI(+) of **Cer-OEG$_8$** at pH 3 after 48 h. OEG$_8$ is the abbreviation for the OEG$_8$-Phosphate.

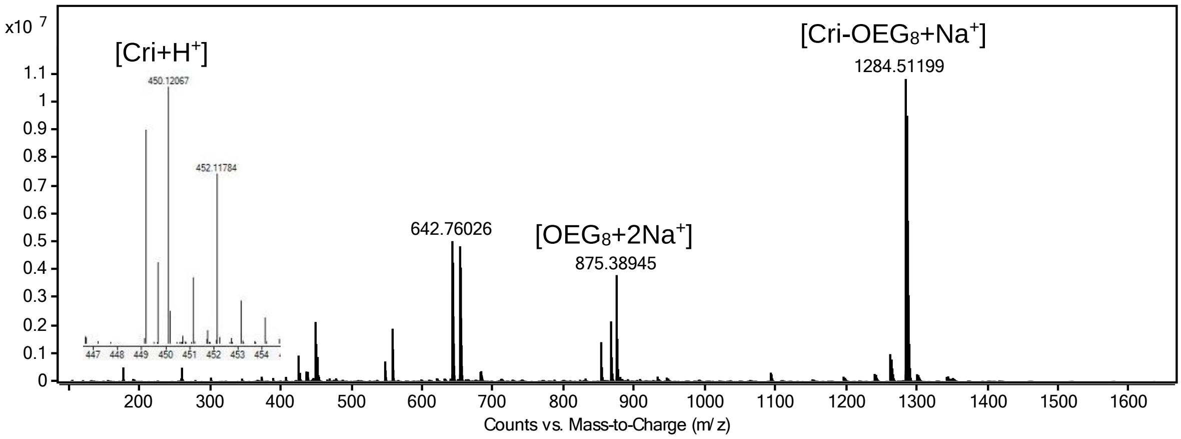

Figure S56: ESI(+) of **Cri-OEG$_8$** at pH 3 after 48 h. OEG$_8$ is the abbreviation for the OEG$_8$-Phosphate.

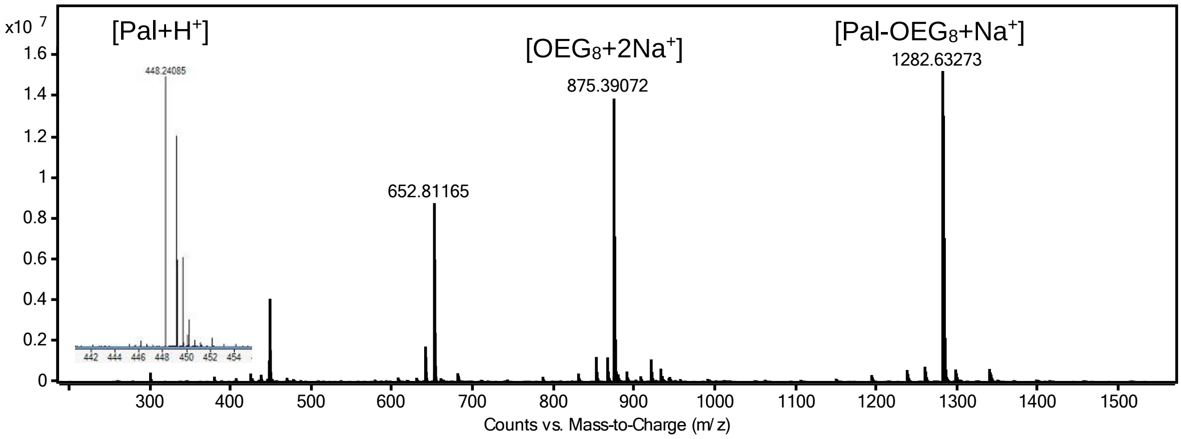

Figure S57: ESI(+) of **Pal-OEG$_8$** at pH 3 after 48 h. OEG$_8$ is the abbreviation for the OEG$_8$-Phosphate.

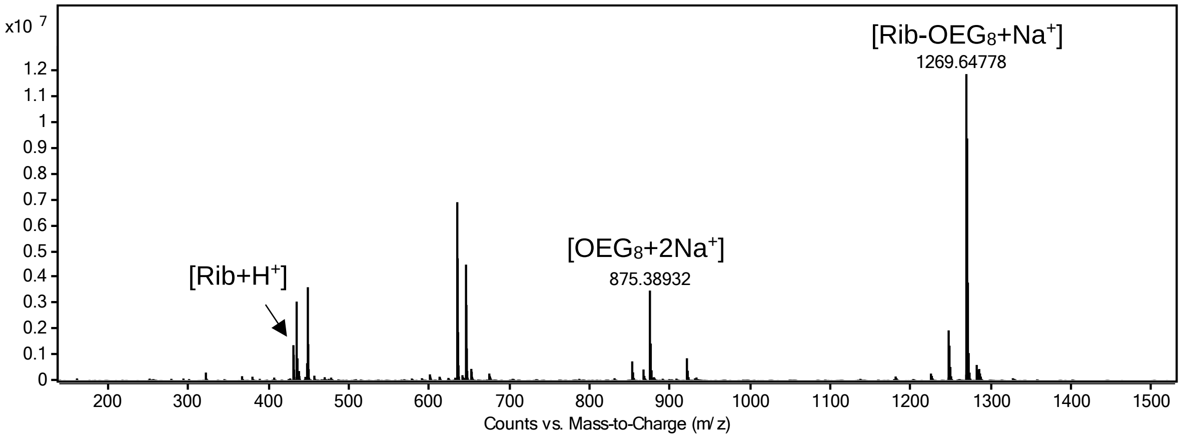

Figure S58: ESI(+) of **Rib-OEG$_8$** at pH 3 after 48 h. OEG$_8$ is the abbreviation for the OEG$_8$-Phosphate.
